# Supplementary material for: Isotype Switching Converts Anti-CD40 Antagonism to Agonism to Elicit Potent Antitumor Activity
Source: Cancer Cell. 2020 Jun 8;37(6):850–866.e7. doi: 10.1016/j.ccell.2020.04.013 (PMC7280789; doi:10.1016/j.ccell.2020.04.013)
Supplement: Document S1. Figures S1–S8 and Table S1 [file mmc1.pdf]

Cancer Cell, Volume *37*

Supplemental Information

Isotype Switching Converts Anti-CD40 Antagonism to Agonism to Elicit Potent Antitumor Activity

Xiaojie Yu, H.T. Claude Chan, Hayden Fisher, Christine A. Penfold, Jinny Kim, Tatyana Inzhelevskaya, C. Ian Mockridge, Ruth R. French, Patrick J. Duriez, Leon R. Douglas, Vikki English, J. Sjef Verbeek, Ann L. White, Ivo Tews, Martin J. Glennie, and Mark S. Cragg

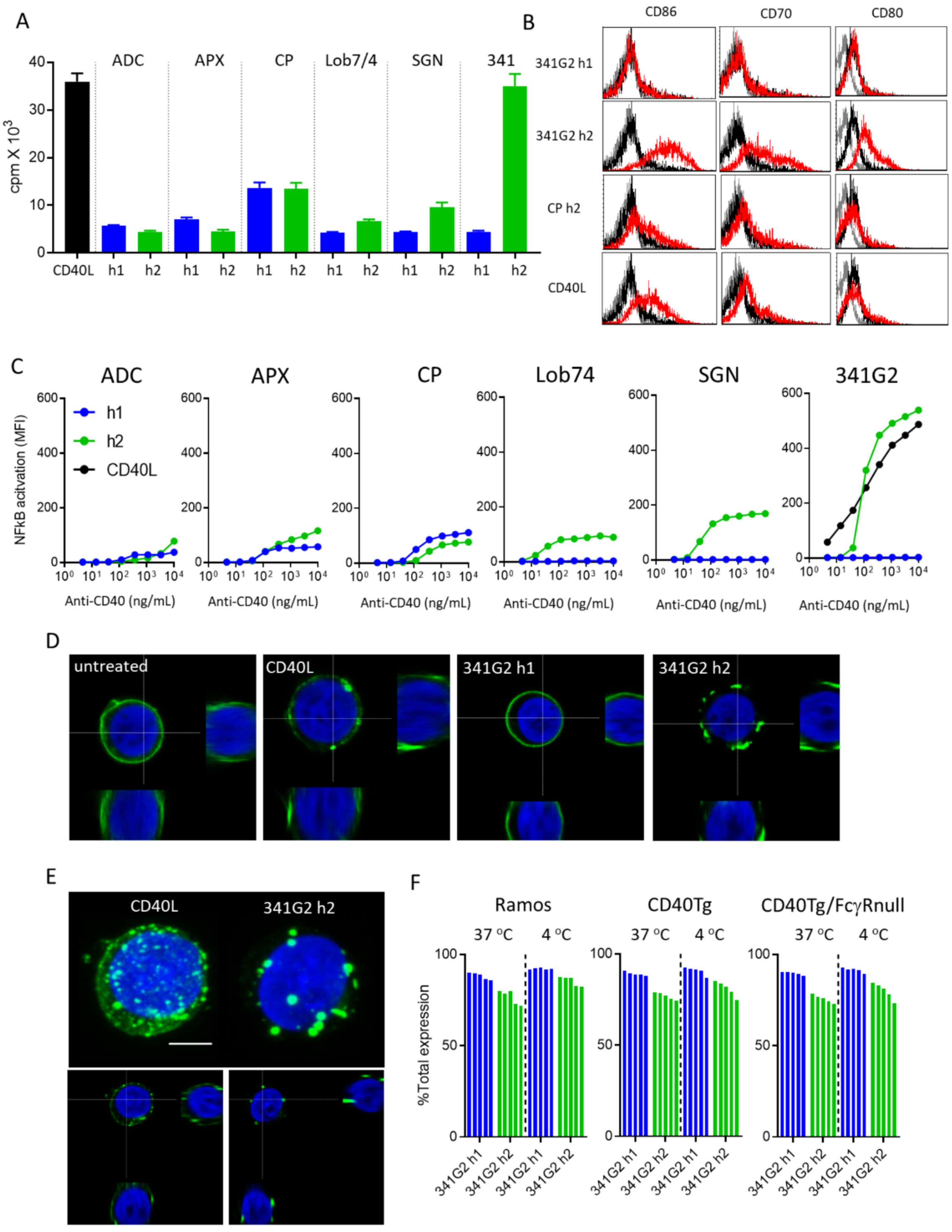
A
B
C
D
E
F
cpm X 10^3
CD40L
ADC
APX
CP
Lob7/4
SGN
341
h1
h2
CD86
CD70
CD80
341G2 h1
341G2 h2
CP h2
ADC
APX
Lob74
341G2
NFkB activation (MFI)
Anti-CD40 (ng/mL)
untreated
Ramos
CD40Tg
CD40Tg/FcγRnull
37 °C
4 °C
%Total expression

Figure S1, Related to Figure 3. Comparison of clinically-relevant anti-CD40 mAb in an NFκB activation assay

(A) Purified human B cells were incubated with 2 µg/mL of various clinical anti-CD40 mAb as indicated for 4 days. Proliferation was measured by ^{3}H thymidine incorporation. Means ± SEM, n = 4, data representative of three donors.

(B) Purified human B cells were incubated with 2 µg/mL of various clinical anti-CD40 mAb as indicated for 2 days and then analysed for surface expression of CD80, CD86 and CD70 by flow cytometry. Data representative of three donors.

(C) Jurkat NFκB GFP reporter cells stably transfected with hCD40 were incubated with various concentrations of different clinically-relevant anti-CD40 mAb for 8 hours and the level of NFκB activation was assessed by GFP expression using flow cytometry. Means ± SEM, n = 3, data representative of two experiments.

(D) Jurkat cells stably transfected with hCD40EC-GFP were treated with 10 µg/mL anti-CD40 mAb as indicated for 1 hour or (E) 24 hours at 37°C. Cells were then fixed, nuclear-stained using DAPI and imaged using a Leica SP8 confocal microscope. Z-stack and orthogonal images shown. Blue: nucleus; Green: hCD40EC-GFP. Scale bar: 4 µm. Image representative of at least ten images taken.

(F) Ramos cells and purified splenic B cells from hCD40Tg or hCD40Tg/FcγRnull mice were treated with AF488-labelled anti-CD40 mAb for 10, 30, 60, 120 or 180 minutes (left to right) at 4°C or 37°C as indicated. Cells were then washed and half the cells were treated with anti-AF488 mAb at 4°C to quench cell surface-associated AF488 fluorescence. Remaining cell surface-bound CD40 was expressed as % Total expression. Data representative of 2-3 experiments.

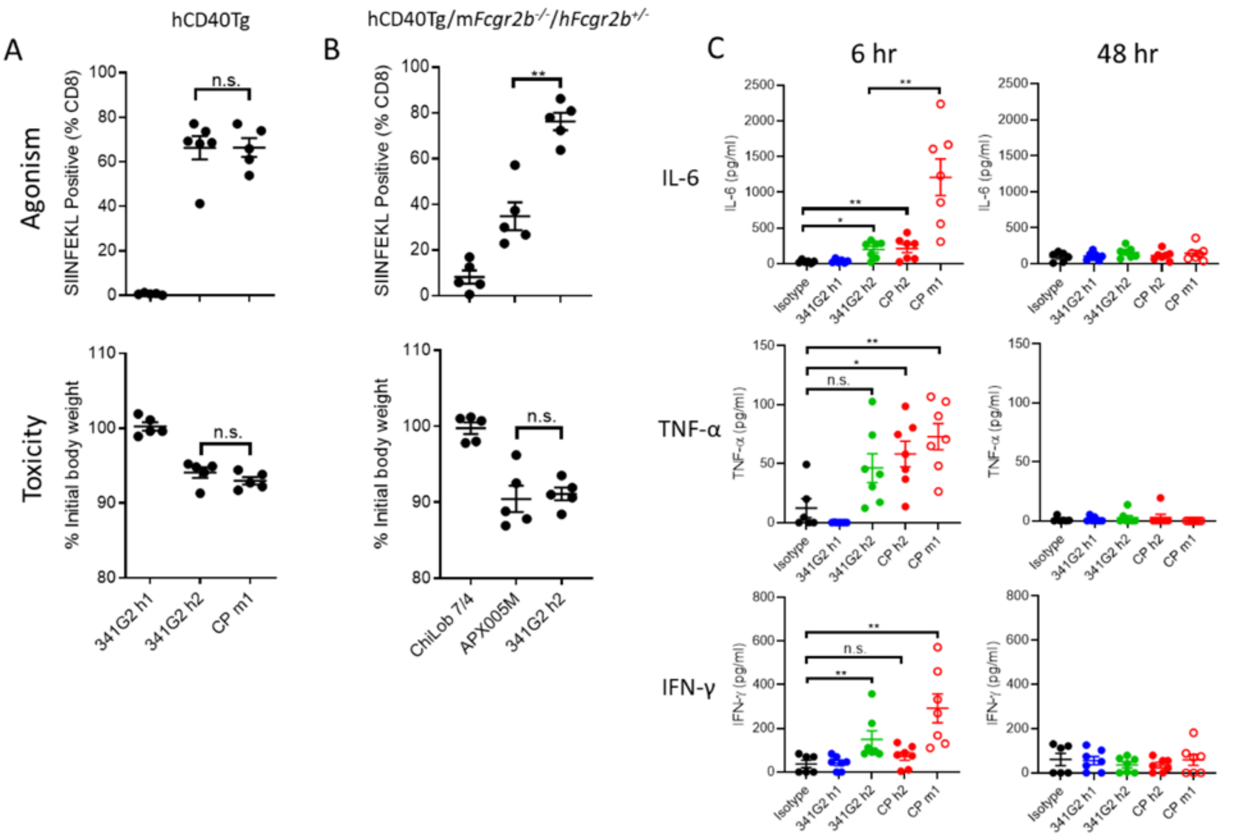

Figure S2, Related to Figure 4. 341G2 h2 induces similar levels of toxicity to other clinically-relevant anti-CD40 agonists

1x10^5 OTI cells were adoptively transferred into (A) hCD40Tg or (B) hCD40Tg/*mFcgr2b$^{-/-}$*/*hFcgr2b$^{+/-}$* mice 1 day before treatment with 30 µg anti-CD40 mAb as indicated. Mice were bled on day 5 and SIINFEKL$^+$ cells were expressed as a percentage of total CD8$^+$ T cells. Toxicity was assessed by loss of body weight after treatment; mice were weighed before treatment and two days after anti-CD40 mAb treatment when weight loss reaches its maximum before recovery. Means ± SEM, n = 5-6. Each dot represents one mouse. Two-tailed, non-paired Student t test, *p < 0.05, **p < 0.01, ***p < 0.001. n.s. not significant.

(C) hCD40Tg mice were administered 30 µg anti-CD40 mAb intravenously and bled 6 hours and 48 hours post-injection. Serum levels of IL-6, TNF-α and IFN-γ were quantified by ELISA. Means ± SEM, n = 6-7. Each dot represents one mouse. Two-tailed, non-paired Student t test, *p < 0.05, **p < 0.01, ***p < 0.001. n.s. not significant.

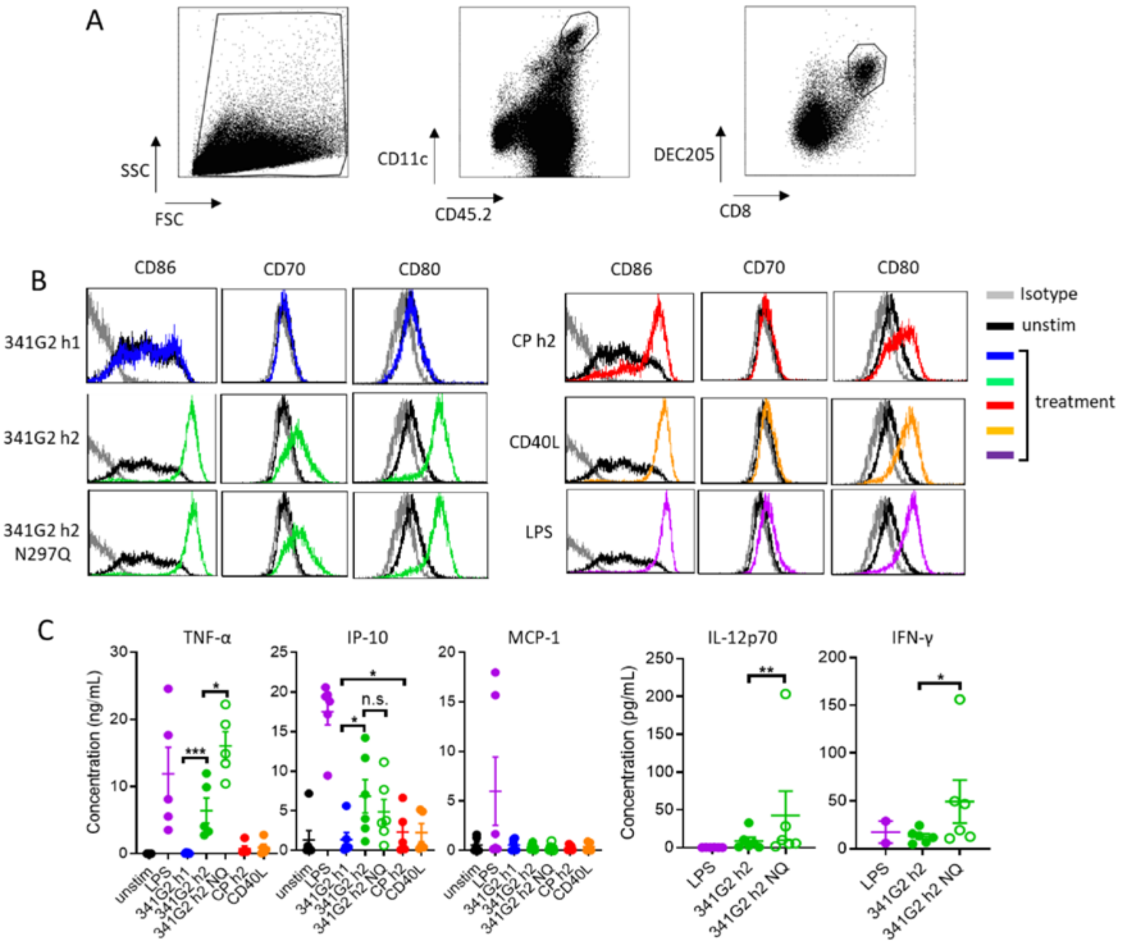

Figure S3, Related to Figure 5. 341G2 h2 activates dendritic cells

(A) Gating strategy for hCD40Tg and hCD40Tg/FcγRnull splenic CD11c^{+}CD8^{+}DEC205^{+} DC subset.

(B) Human monocyte-derived DCs were stimulated with various anti-CD40 mAb, CD40L or LPS as indicated for 48 hours and then stained for CD86, CD70 and CD80. Data representative of six donors.

(C) Experiment the same as (B). Cell culture supernatant was harvested after 48 hours and analysed for the level of TNF-α, IP-10, MCP-1, IL-12p70 and IFN-γ using Luminex. Means ± SEM, data pooled from five-six donors. Each dot represents one donor. Two-tailed, paired Student t test, *p < 0.05, **p < 0.01, ***p < 0.001. n.s. not significant.

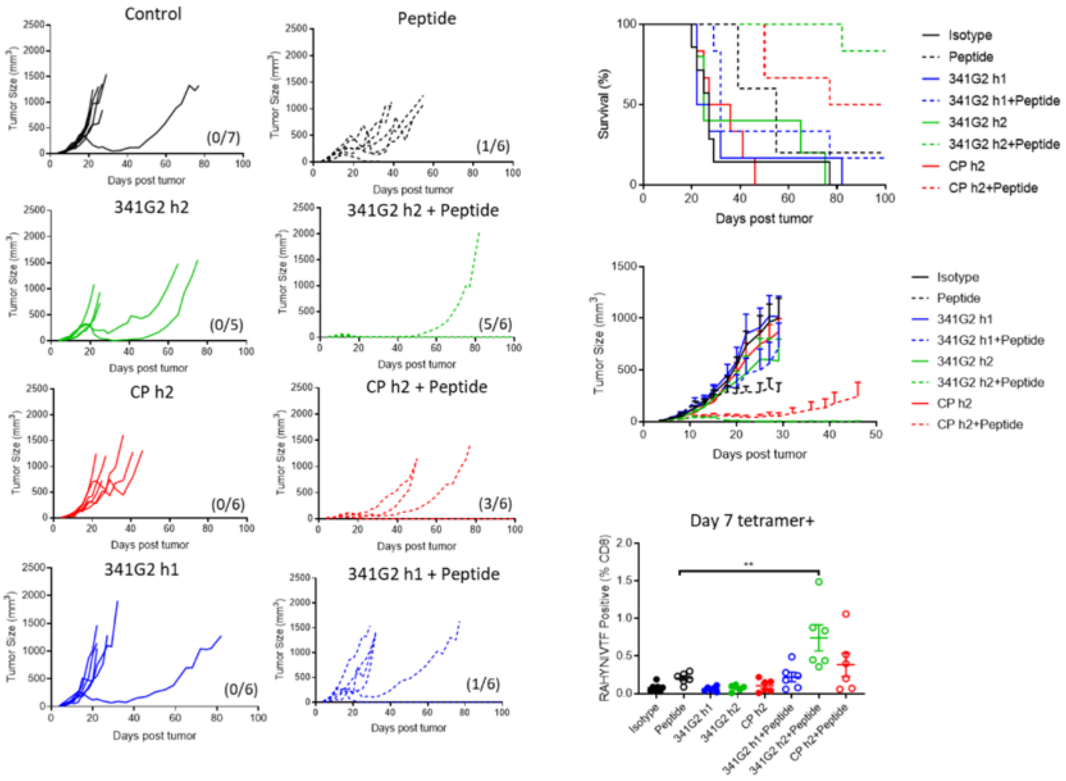

Figure S4, Related to Figure 7. Antitumor efficacy of anti-CD40 mAb in combination with peptide vaccination

hCD40Tg mice were inoculated with $1x10^5$ TC1 tumor cells subcutaneously on day 0 and then were treated with 3 µg peptide in combination with 30 µg anti-CD40 mAb on day 5, or treated with 30 µg anti-CD40 mAb alone on day 5, 8 and 11. n = 5-7, each dot represents one mouse. Two-tailed, non-paired Student t test. The fractions in parenthesis indicate the number of tumor-free mice (numerator) out of total mice (denominator) in that group at the end of the study. Survival curves were compared by Log-rank test. *p < 0.05, **p < 0.01, ***p < 0.001.

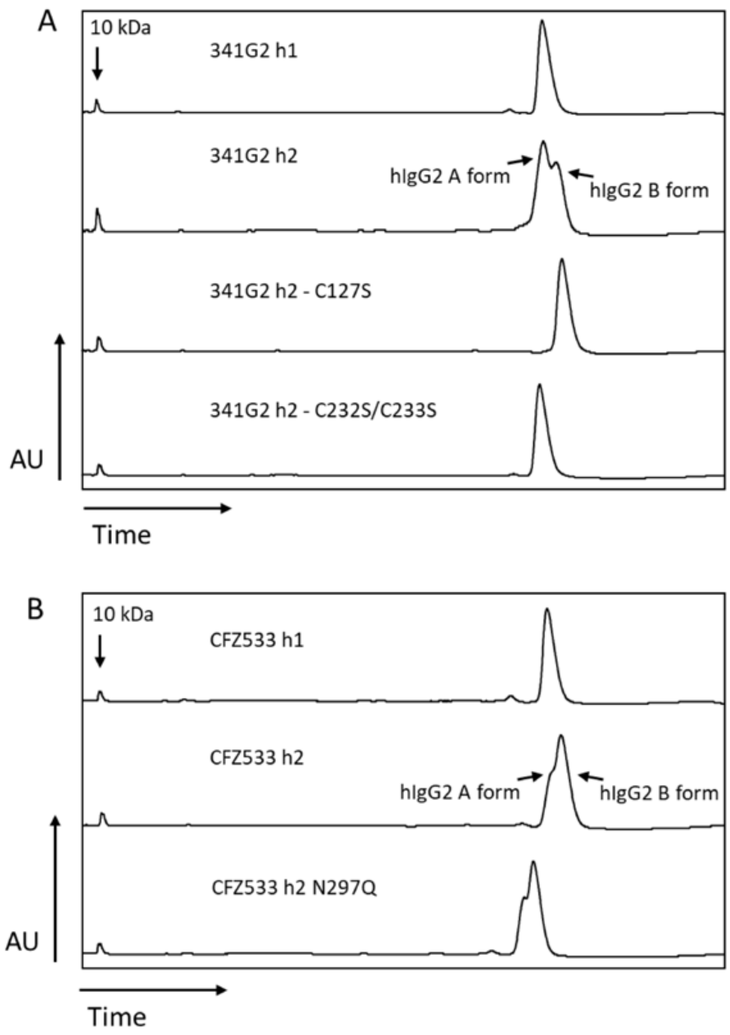

Figure S5, Related to Figure 8. CE-SDS profiles of anti-CD40 mAb

Non-reducing CE-SDS was carried out for (A) 341G2 Fc variants and (B) CFZ533 Fc variants. Data representative of at least three experiments.

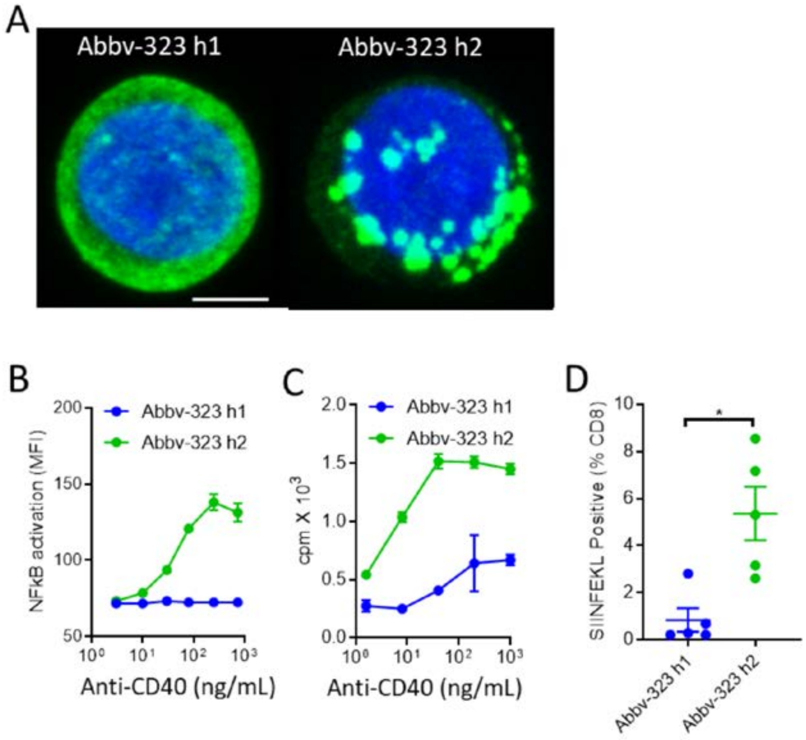

Figure S6, Related to Figure 8. Antagonist Abbv-323 is converted into an agonist by isotype switching to hIgG2

(A) Jurkat cells stably transfected with human CD40EC-GFP were treated with 10 µg/mL Abbv-323 h1 or Abbv-323 h2 for one hour at 37°C. Cells were then fixed, nuclear-stained using DAPI and imaged using a Leica SP8 confocal microscope. Z-stack images shown. Blue: nucleus; Green: human CD40-GFP. Scale bar: 4 µm. Image representative of at least ten images taken.

(B) Jurkat NFκB GFP reporter cells stably transfected with hCD40 were incubated with various concentrations of anti-CD40 mAb for 8 hours and the level of NFκB activation assessed by GFP expression using flow cytometry. n = 3, data representative of 2 experiments.

(C) Purified splenic B cells from hCD40Tg mice were incubated with various concentrations of anti-CD40 mAb for 4 days. Proliferation was measured by ^{3}H thymidine incorporation. Means ± SEM, n = 3, data representative of 3 experiments.

(D) $1x10^5$ OTI cells were adoptively transferred into hCD40Tg mice 1 day before treatment with 100 µg anti-CD40 mAb as indicated. Mice were bled on day 5 and SIINFEKL$^+$ cells were expressed as the percentage of total CD8$^+$ T cells. n = 5, each dot represents one mouse, data representative of two experiments. Two-tailed, non-paired Student t test, *p < 0.05, **p < 0.01, ***p < 0.001.

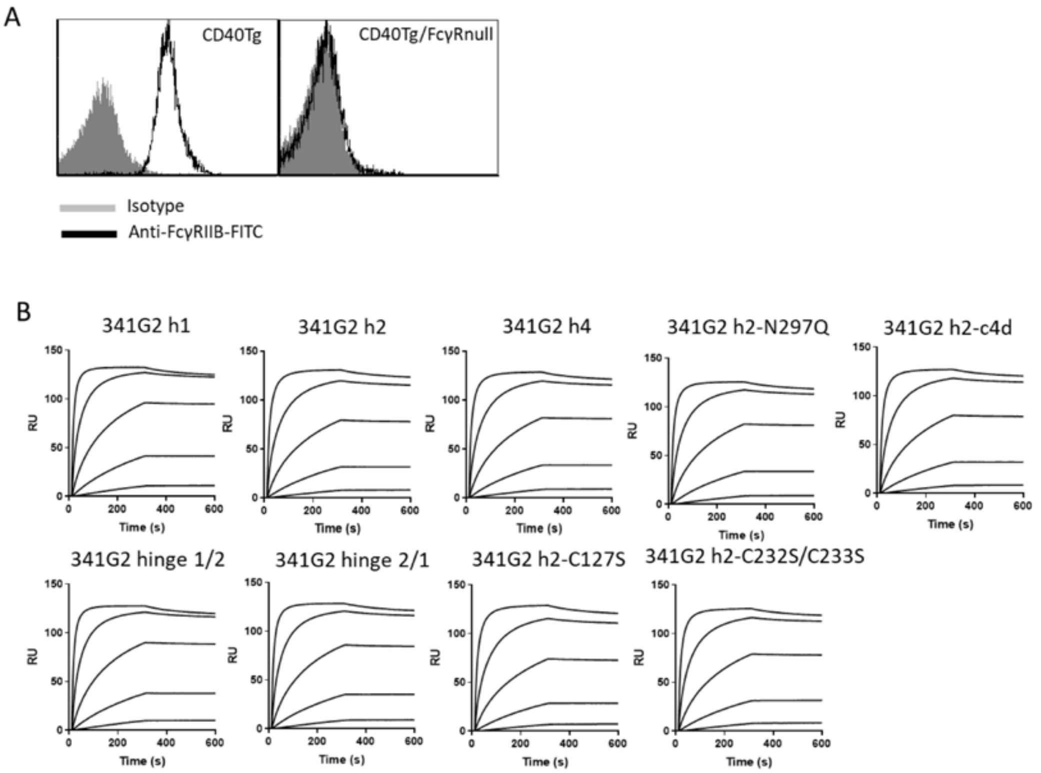

Figure S7, Related to STAR Methods. Binding analysis of 341G2 variants to hCD40 by SPR

(A) Expression of FcγRIIB on B cells from peripheral blood of hCD40Tg or hCD40Tg/FcγRnull mice analysed by anti-CD19-APC and anti-FcγRIIB-FITC, demonstrating the absence of FcγRIIB on the hCD40Tg/FcγRnull B cells.

(B) hCD40 was immobilized onto CM5 chip and various 341G2 variants were injected at 250, 50, 10, 2, 0.4, and 0 nM using Biacore T100 instrument. The association and dissociation phases lasted 300 seconds, respectively.

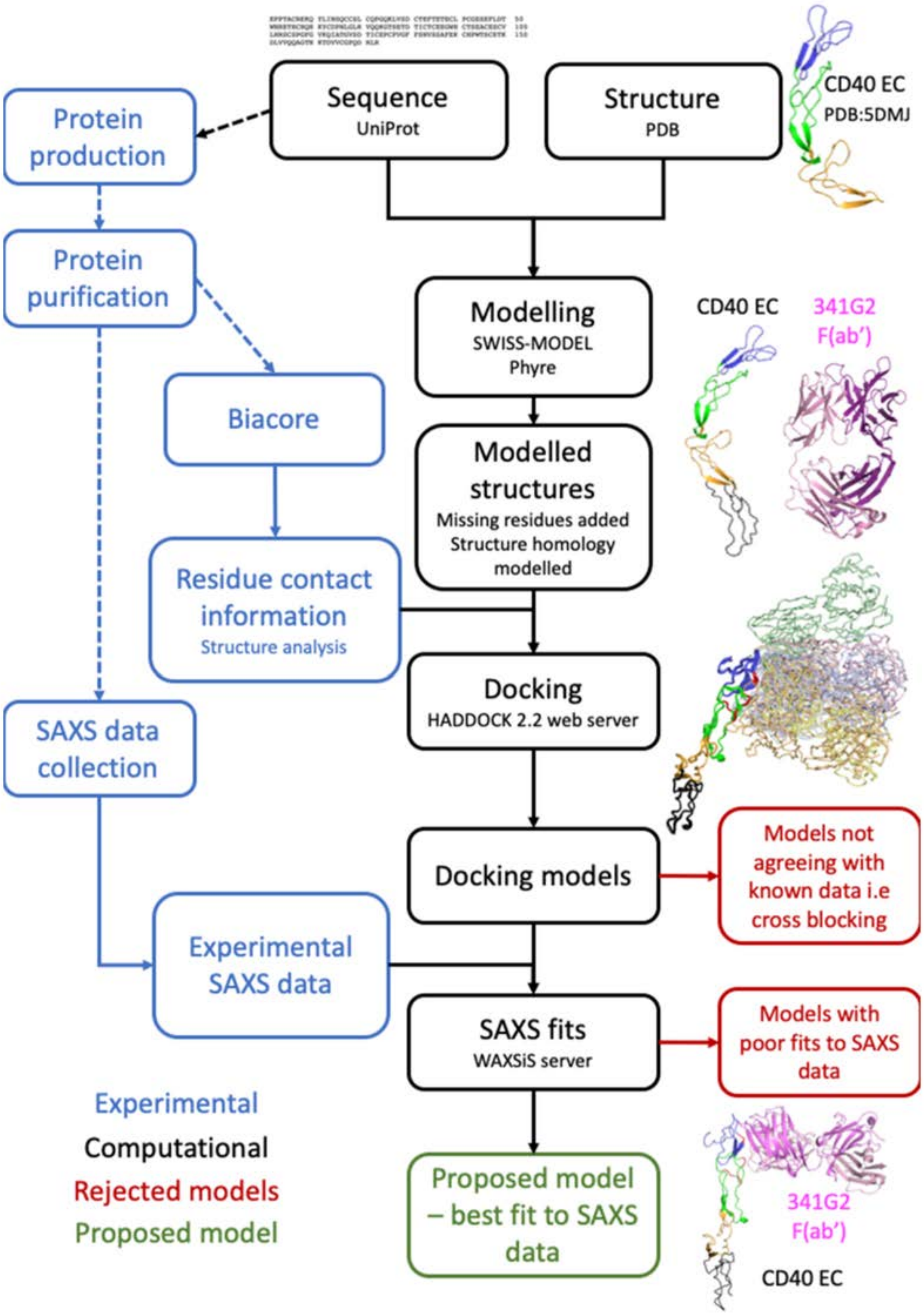
Sequence
UniProt
Structure
PDB
CD40 EC
PDB:5DMJ
Protein production
Protein purification
Biacore
Residue contact information
Structure analysis
SAXS data collection
Experimental SAXS data
Modelling
SWISS-MODEL
Phyre
CD40 EC
341G2 F(ab')
Modelled structures
Missing residues added
Structure homology modelled
Docking
HADDOCK 2.2 web server
Docking models
Models not agreeing with known data i.e cross blocking
SAXS fits
WAXSiS server
Models with poor fits to SAXS data
Proposed model – best fit to SAXS data
341G2 F(ab')
CD40 EC
Experimental
Computational
Rejected models
Proposed model

Figure S8, Related to STAR Methods. Generation of proposed binding model of 341G2 F(ab) and CD40 EC domain.

The workflow details the steps taken to generate the proposed binding model between CD40 EC and 341G2 F(ab). Initially, sequence information was examined and that used for the modelling compared with that used to generate the protein for experimental data collection, to ensure reliable SAXS fitting. Available structures within the PDB database were then examined to identify structures containing residues to cover the sequences of interest. Where no structure was available (341G2), the sequence was submitted to the SWISS-MODEL server to generate a homology model. The model was generated based on the 56.a.09 antibody (PDB: 5K9J) with which 341G2 Fab shares 90.4% sequence identity and 0.57 sequence similarity score. For CD40 - Phyre2 was used to generate a model of the CD40EC. The CD40EC sequence (Uniprot: P25942) was submitted for modelling, returning a model based on PDB: 5DMJ that had a confidence score of 100% with 97% coverage indicating a suitable model for further analysis. Docking of 341G2 F(ab) and CD40 was performed using the HADDOCK 2.2 webserver. Residues on CD40 known to affect binding (identified through experimental data collection using alanine scanning mutagenesis experiments and Biacore), and the CDR loop residues of 341G2 were provided to the docking server as restraints in the docking following the HV-Epi9 protocol. For CD40 these residues were S49, D50, F67, L68, T75, H76, H78 and Q79. For the 341G2 model, the CDR loop residues were 26 to 34, 53 to 59, 104 to 112, 260 to 268, 284 to 288 and 325 to 331. The CDR loops were defined as active residues, while the CD40 residues were defined as active with a 9Å radius of passive residues. Docking produced 10 clusters comprising 40 representative structures. Based on the knowledge that 341G2 and ChiLob 7/4 do not cross-block, a large number of the representative models were able to be excluded due to clashing with the Chilob 7/4 Fab from the crystal structure PDB: 6FAX when aligned on CD40. This left a total of 11 models from 3 clusters that were taken forward for further analysis. The remaining structures were validated against the SAXS data using the WAXSiS server to compare the structures to the experimental SAXS data. The models that best fitted the SAXS data were selected by their $\chi2$ score. A clear trend was observed between better and worse fitting models when looking at the SAXS fits ($\chi2$ range 2.21±0.37 to 4.50±0.47), which was characterised by a movement of the F(ab) around the CD40 surface when comparing multiple models. It is also known that 341G2 cannot bind when CD40L is present, allowing comparison with PDB: 3QD6, to ensure that either the CD40 binding interface for the binding models and CD40L are shared or steric clashes occur between CD40L and the F(ab). In this case all models showed clashes with CD40L. The model showing the best fit to the SAXS data ($\chi2$ = 2.21±0.37) was therefore selected as the proposed binding model, as it showed no cross blocking with ChiLob7/4, clashed with CD40L and made contact with residues shown to be critical for binding from the biacore mutagenesis data.

Table S1, Related to STAR Methods. Affinity of 341G2 variants for hCD40

	k_{on} (s^{-1} M^{-1})	k_{off} (s^{-1})	KD (M)	Standard Deviation
**341G2 hg1**	6.17×10^{-5}	2.38×10^{-4}	3.87×10^{-10}	1.78×10^{-10}
**341G2 hg2**	4.39×10^{-5}	2.47×10^{-4}	5.58×10^{-10}	2.24×10^{-10}
**341G2 hg4**	4.77×10^{-5}	2.21×10^{-4}	4.60×10^{-10}	1.90×10^{-10}
**341G2 hg2-NQ**	4.95×10^{-5}	2.37×10^{-4}	4.77×10^{-10}	2.17×10^{-10}
**341G2 hg2-c4d**	4.66×10^{-5}	2.33×10^{-4}	5.04×10^{-10}	2.43×10^{-10}
**341G2 hinge 1/2**	6.34×10^{-5}	2.47×10^{-4}	3.80×10^{-10}	1.25×10^{-10}
**341G2 hinge 2/1**	5.37×10^{-5}	2.45×10^{-4}	4.47×10^{-10}	1.79×10^{-10}
**341G2 h2-C127S**	4.18×10^{-5}	2.57×10^{-4}	6.17×10^{-10}	2.14×10^{-10}
**341G2 h2-C232S/C233S**	4.58×10^{-5}	2.21×10^{-4}	4.77×10^{-10}	1.87×10^{-10}
