# Supplementary material for: Isotype Switching Converts Anti-CD40 Antagonism to Agonism to Elicit Potent Antitumor Activity
Source: Cancer Cell. 2020 Jun 8;37(6):850–866.e7. doi: 10.1016/j.ccell.2020.04.013 (PMC7280789; doi:10.1016/j.ccell.2020.04.013)
Supplement: Document S2. Article plus Supplemental Information [file mmc2.pdf]

Cancer Cell

Article

Isotype Switching Converts Anti-CD40 Antagonism to Agonism to Elicit Potent Antitumor Activity

Graphical Abstract

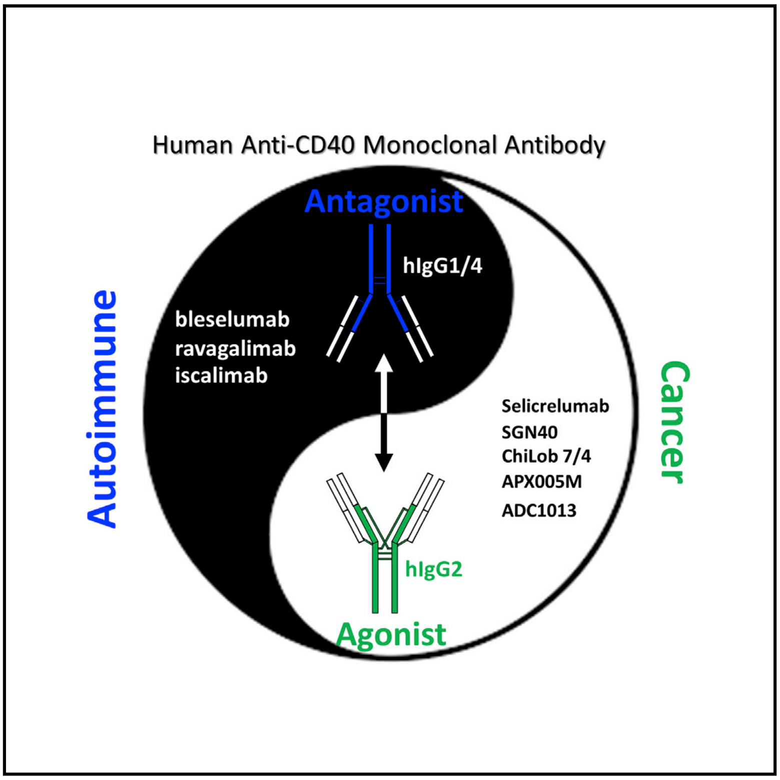

Highlights

- Antagonistic anti-CD40 mAbs can be converted into agonists by isotype switching to hIgG2
- Transformation is based upon the hIgG2 hinge
- Transforms an antagonist to an agonist four times more potent than existing anti-CD40 mAbs
- This converted antagonist exhibits antitumor synergy with cell therapy and vaccination

Authors

Xiaojie Yu, H.T. Claude Chan, Hayden Fisher, ..., Ivo Tews, Martin J. Glennie, Mark S. Cragg

Correspondence

x.yu@soton.ac.uk (X.Y.), msc@soton.ac.uk (M.S.C.)

In Brief

Yu et al. show that isotype switching can convert clinically relevant anti-CD40 antagonistic antibodies to potent FcγR-independent agonists. The converted antibodies can elicit strong antitumor responses in mouse models.

Yu et al., 2020, Cancer Cell *37*, 850–866
June 8, 2020 © 2020 The Author(s). Published by Elsevier Inc.
https://doi.org/10.1016/j.ccell.2020.04.013

CellPress

CellPress
OPEN ACCESS

Cancer Cell

Article

Isotype Switching Converts Anti-CD40 Antagonism to Agonism to Elicit Potent Antitumor Activity

Xiaojie Yu,[1,*] H.T. Claude Chan,[1] Hayden Fisher,[1,2,3] Christine A. Penfold,[1] Jinny Kim,[1] Tatyana Inzhelevskaya,[1] C. Ian Mockridge,[1] Ruth R. French,[1] Patrick J. Duriez,[4] Leon R. Douglas,[4] Vikki English,[6] J. Sjef Verbeek,[5] Ann L. White,[1,7] Ivo Tews,[2,3] Martin J. Glennie,[1] and Mark S. Cragg[1,2,8,*]

[1]Antibody and Vaccine Group, Cancer Sciences Unit, University of Southampton Faculty of Medicine, Southampton, UK
[2]Institute for Life Sciences, University of Southampton, Southampton, UK
[3]Biological Sciences, University of Southampton, Highfield Campus, Southampton SO17 1BJ, UK
[4]CRUK Protein Core Facility, University of Southampton Faculty of Medicine, Southampton, UK
[5]Department of Human Genetics, Leiden University Medical Centre, Leiden, the Netherlands
[6]Pre-clinical Unit, University of Southampton Faculty of Medicine, Southampton, UK
[7]Present address: UCB-Celltech, 216 Bath Road, Slough SL1 3WE, UK
[8]Lead Contact
*Correspondence: x.yu@soton.ac.uk (X.Y.), msc@soton.ac.uk (M.S.C.)
https://doi.org/10.1016/j.ccell.2020.04.013

SUMMARY

Anti-CD40 monoclonal antibodies (mAbs) comprise agonists and antagonists, which display promising therapeutic activities in cancer and autoimmunity, respectively. We previously showed that epitope and isotype interact to deliver optimal agonistic anti-CD40 mAbs. The impact of Fc engineering on antagonists, however, remains largely unexplored. Here, we show that clinically relevant antagonists used for treating autoimmune conditions can be converted into potent FcγR-independent agonists with remarkable antitumor activity by isotype switching to hIgG2. One antagonist is converted to a super-agonist with greater potency than previously reported highly agonistic anti-CD40 mAbs. Such conversion is dependent on the unique disulfide bonding properties of the hIgG2 hinge. This investigation highlights the transformative capacity of the hIgG2 isotype for converting antagonists to agonists to treat cancer.

INTRODUCTION

CD40 is a costimulatory tumor necrosis factor (TNF) receptor widely expressed on immune and non-immune cell types (Elgueta et al., 2009; Lievens et al., 2009). The interaction between CD40 and its endogenous ligand CD40L is critical for mounting an effective immune response against exogenous pathogens and naturally arising tumors. Consequently, a breakdown in the homeostasis of the CD40/CD40L axis leads to both immunodeficiency and autoimmunity (Karnell et al., 2019; Senhaji et al., 2015). For example, patients with CD40 deficiency exhibit hyper IgM syndrome and are more susceptible to infections; while CD40 over-stimulation is implicated in various autoimmune syndromes, such as lupus and colitis (Banchereau et al., 1994; Peters et al., 2009). Moreover, CD40-mediated allogeneic T cell responses constitute a major mechanism of transplant rejection (Larsen and Pearson, 1997; Pinelli and Ford, 2015). These opposing immune pathologies have led to the development of two distinct classes of anti-CD40 antibodies that selectively modulate the CD40/CD40L axis.

Agonistic anti-CD40 mAbs mimic signals from CD40L-expressing helper $CD4^+$ T cells to activate antigen-presenting cells, such as dendritic cells (DC), to provide signals for the licencing and expansion of $CD8^+$ CTL (Bennett et al., 1998; Ridge et al.,

Significance

Immunomodulatory monoclonal antibodies (mAbs) are providing powerful treatments for human disease. CD40 is a key regulator of adaptive immunity. mAbs binding the receptor can either drive agonism for liberating anti-cancer immunity or antagonize its activities to limit autoimmunity. Here, we demonstrate that clinically relevant antagonistic mAbs can be converted to super-agonistic reagents capable of potent tumor control through isotype switching to hIgG2. This knowledge will help guide the development of the next generation of anti-CD40 reagents for the clinic.

 © 2020 The Author(s). Published by Elsevier Inc.
This is an open access article under the CC BY license (http://creativecommons.org/licenses/by/4.0/).

1998; Schoenberger et al., 1998). Following impressive results in mouse models (French et al., 1999; Todryk et al., 2001; Tutt et al., 2002; van Mierlo et al., 2002), at least six mAbs have entered clinical trials for various cancer indications (Vonderheide, 2019; Vonderheide and Glennie, 2013). We have previously shown that the antibody epitope drives the agonistic nature of anti-CD40 mAbs, with mAbs that target the membrane distal cysteine-rich domain 1 (CRD1) exhibiting agonism, while those that target CRD2-4 block CD40L binding and display antagonistic activity (Yu et al., 2018). In addition to epitope, the selection of isotype, which differentially modulates Fc-FcγR interaction, also significantly influences agonistic activity (White et al., 2015). Among the agonistic anti-CD40 mAbs in clinical trials, all but CP870,893 and CDX-1140 are human IgG1 (hIgG1) and require their Fc domain for full activity in preclinical models, consistent with the paradigm that most anti-CD40 mAbs require the inhibitory FcγRIIB for agonistic activity (Li and Ravetch, 2011; White et al., 2011).

Recently, however, such Fc-dependent activity was found to be dispensable and could be supplanted by isotype switching to human IgG2 (hIgG2), which imparts superior Fc-independent agonistic activity (White et al., 2015). Indeed, both CP870,893 and CDX-1140 entered clinical trials as hIgG2 and were found to retain at least a proportion of their activity in the absence of FcγR interaction (Richman and Vonderheide, 2014; Vitale et al., 2019). hIgG2 contains two additional cysteine residues in the CH1 and hinge regions compared with hIgG1, which enables differential disulfide bonding and leads to the generation of a heterogeneous population of isoforms that differ in their conformational rigidity (Dillon et al., 2008; Martinez et al., 2008; Wypych et al., 2008). Functional characterization showed that the so-called "B" form retained agonistic activity, while the "A" form was relatively inactive (White et al., 2015). Selective mutation of cysteine residues within the hIgG2 CH1 and hinge domains allows recombinant production of homogeneous forms locked into either configuration (White et al., 2015).

In contrast to agonists, antagonistic anti-CD40 mAbs block CD40/CD40L interaction to abrogate downstream signaling and suppress unwanted immune responses (Karnell et al., 2019; Lai et al., 2019). To date, at least five antagonistic anti-CD40 mAbs have entered clinical trials for various autoimmune diseases, including Graves' hyperthyroidism (Kahaly et al., 2019), primary Sjogren's syndrome (Fisher et al., 2017), rheumatoid arthritis (Visvanathan et al., 2016), plaque psoriasis (Anil Kumar et al., 2018), Crohn disease (Kasran et al., 2005), and ulcerative colitis (NCT03695185), as well as for transplant rejection (Farkash et al., 2019). Contrary to the varied isotype selection for agonists, antagonistic anti-CD40 mAbs have been unanimously engineered to exhibit minimal Fc effector function. As such, all clinical antagonists are either human IgG4 (hIgG4) or hIgG1 engineered for minimal FcγR and complement engagement, which likely correlates with their positive safety profiles in clinical trials (Karnell et al., 2019; Lai et al., 2019). Such safety profiles also support the notion that the toxicity observed with agonists in patients is mediated through FcγR engagement, as was recently demonstrated for another TNF receptor, 4-1BB (Claus et al., 2019; Segal et al., 2017).

While epitope divides anti-CD40 mAbs into agonists and antagonists based on their intrinsic ability to block CD40/CD40L engagement, the effect of isotype and particularly the differential disulfide bonding pattern conferred by hIgG2 on antagonists remains unknown. Therefore, here we aimed to address these issues with respect to a series of clinically relevant antagonist mAbs.

RESULTS

Epitope Characterization of Antagonistic Anti-CD40 mAb 341G2

341G2 (bleselumab) is an hIgG4 antagonistic anti-CD40 mAb in clinical trials for plaque psoriasis and kidney transplant rejection (Anil Kumar et al., 2018; Harland et al., 2017). Extensive preclinical work in non-human primates demonstrates its safety and efficacy in prolonging survival of renal, pancreatic islet, and hepatic allografts (Imai et al., 2007; Oura et al., 2012; Watanabe et al., 2013). To characterize its binding epitope, we generated CHO-k1 cells expressing either the full-length CD40 molecule or truncated variants comprising one, two, or three of its CRDs. Consistent with previous reports, ChiLob 7/4, whose epitope was defined within CRD1 by X-ray crystallography (Yu et al., 2018), bound to cells expressing the full-length CD40 but not to variants lacking CRD1 (Figure 1A), while Lob 7/6 bound to all variants containing the CRD3 domain. Similar to ChiLob 7/4, 341G2 only bound to cells expressing the full-length CD40 molecule, indicating an epitope within CRD1 (Figure 1A). This was further supported by western blotting of similar soluble CD40 variants showing 341G2 only bound to the full-length CD40 protein (Figure 1B). As domain truncation might destabilize the protein structure, we performed alanine-scanning mutagenesis in which two consecutive residues were mutated to alanines to minimize structural disruption. Alanine-scanning mutagenesis confirmed that 341G2 binds to CRD1 but also indicated interaction with CRD2, with the majority of indicated contact residues located within CRD2 (Figures 1C and 1D). To better visualize the binding epitope, a 341G2 Fab-CD40 complex was generated, purified by size-exclusion chromatography (SEC) and crystallization trials performed alongside small-angle X-ray scattering (SAXS). No diffractable crystals were generated and so we performed homology modeling of 341G2 coupled to docking analysis, using the available knowledge of binding criteria as constraints: the surface residues of CD40 CRD1/2 identified through alanine-scanning mutagenesis to be in contact with 341G2 and the 341G2 CDR sequence information. Docking produced 40 representative models, which were refined to 11 models based upon further known constraints, such as lack of cross-blocking with Lob 7/4 (see the STAR Methods). These remaining structures were validated against the SAXS data using the WAXSiS server (Knight and Hub, 2015). The best fitting model gave a $\chi 2$ score of 2.21 ± 0.37 indicating a good fit to the experimental data. Consistent with results from alanine-scanning mutagenesis, the derived model indicates that 341G2 engages both CRD1 and CRD2, overlapping with the CD40L binding interface, and diametrically opposite the ChiLob 7/4 binding site (Figure 1E). Thus, 341G2 is distinct among previously characterized anti-CD40 mAbs in that binding is not limited to any single CRD but rather spreads between CRD1 and CRD2.

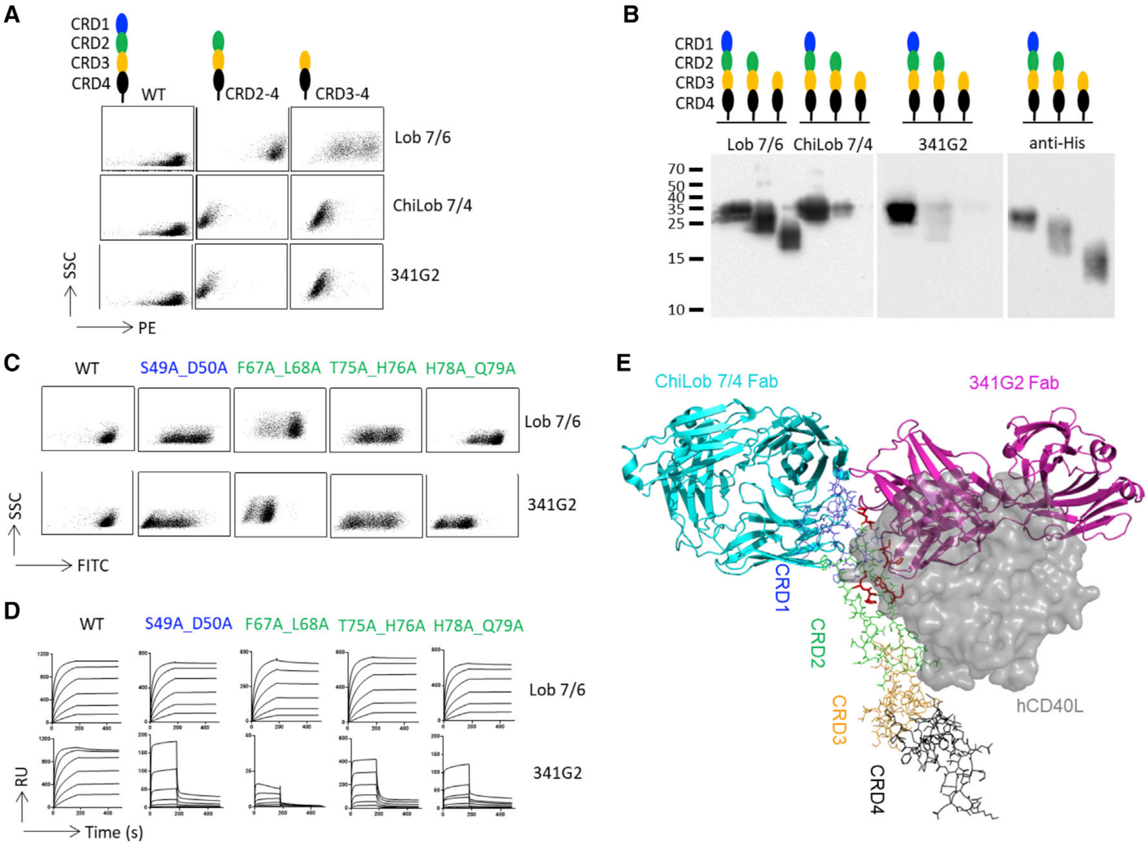

Figure 1. Antagonist Anti-CD40 mAb 341G2 Binds CRD1 and CRD2 Domains at the CD40/CD40L Interface

(A) CHO-k1 cells expressing full-length hCD40 or domain-truncated variants encompassing CRD2-4 or CRD3-4 were incubated with 10 μg/mL anti-CD40 mAb Lob 7/6, ChiLob 7/4, or 341G2. Bound anti-CD40 mAb was detected by anti-human Fc-PE.

(B) His-tagged recombinant soluble proteins corresponding to the full-length extracellular domain (EC) of hCD40 or its truncated variants were analyzed by western blotting with the mAb indicated above each panel used for detection. Image represents a composite of multiple blots.

(C) CHO-k1 cells expressing different hCD40 mutants were probed with anti-CD40 mAbs. Bound mAbs was detected by anti-mouse IgG-FITC.

(D) His-tagged soluble hCD40 mutants were captured for 30 s using anti-His mAbs immobilized on a CM5 chip, and Lob 7/6 and 341G2 were injected at 1,000, 333, 111, 37, 12.4, and 4.1 nM using a Biacore T100 instrument. The association and dissociation phases lasted 180 and 300 s, respectively.

(E) Structural prediction of 341G2 Fab-hCD40 by SEC-SAXS followed by homology modeling and docking analysis. 341G2 Fab homology modeled as described in the methods shown in magenta cartoon representation. CD40 EC represented as CRD colored sticks. ChiLob 7/4 shown as cyan cartoon overlaid with CD40 (PDB: 6FAX). CD40L shown as a gray surface (PDB: 3QD6) shows overlap with proposed 341G2 Fab position. CD40 residues involved in 341G2 interaction highlighted as red sticks.

Antagonist Anti-CD40 mAbs Inhibit CD40L-Mediated Functions

To characterize the antagonistic nature of 341G2, we studied its ability to inhibit CD40L-mediated activities. Both the parental 341G2 hIgG4 and its hIgG1 variant inhibited the binding of CD40L to CD40-expressing Ramos cells in a dose-dependent but isotype-independent manner (Figure 2A), consistent with the fact that steric blockade of CD40/CD40L interaction by antagonists is independent of the Fc domain. Moreover, both 341G2 hIgG4 and 341G2 hIgG1 were able to inhibit CD40L-mediated hCD40Tg mouse splenic B cell and human B cell proliferation and homotypic cell-cell adhesion (Figures 2B and 2C). To confirm the immunosuppressive effect of 341G2 *in vivo*, mice were immunized with ovalbumin (OVA) protein and the generation of anti-OVA IgG quantified. We found that both 341G2 hIgG4 and 341G2 hIgG1 significantly reduced the level of anti-OVA IgG in serum (Figure 2D); alongside a concomitant decrease in the level of circulating B cells (Figure 2E), a phenomenon observed in clinical trials of anti-CD40 mAbs possessing an intact Fc domain (Vonderheide, 2019). To confirm that the reduction in anti-OVA IgG in serum was not due to peripheral B cell depletion, we generated deglycosylated 341G2 hIgG1, lacking Fc effector functions (Lux et al., 2013; Nimmerjahn and Ravetch, 2008), which effectively suppressed the anti-OVA IgG response but did not result in B cell depletion (Figures 2D and 2E).

Isotype Switching to hIgG2 Converts Antagonist to Super-agonist

We previously showed that isotype switching to hIgG2 could significantly enhance the activity of agonistic anti-CD40 mAbs; however, the effect of hIgG2 on anti-CD40 antagonists has not been investigated. Therefore, we explored the impact of switching 341G2 to the hIgG2 isotype. *In vitro* functional assays showed that both 341G2 hIgG1 and 341G2 hIgG4 failed to

CellPress
OPEN ACCESS

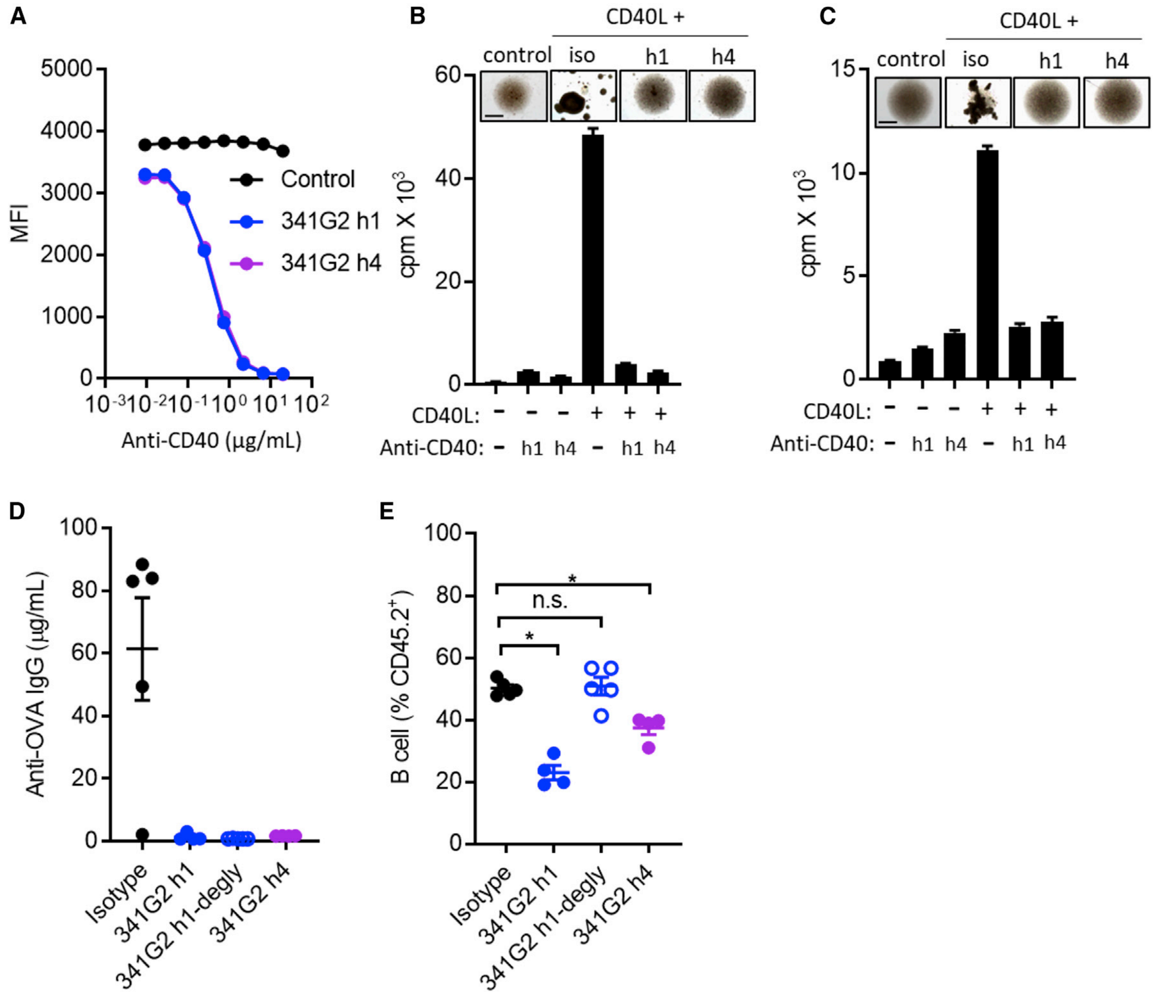

Figure 2. 341G2 hIgG1 (h1) and hIgG4 (h4) Suppress Immune Function *In Vitro* and *In Vivo*

(A) Ramos cells were incubated with fixed concentration of CD40L and various concentrations of anti-CD40 mAbs. Remaining bound CD40L was detected by anti-FLAG-APC. Means ± SEM, n = 3, data representative of three experiments.

(B) Purified hCD40Tg mouse splenic B cells were incubated with 2 μg/mL CD40L in the presence or absence of 5 μg/mL 341G2 h1 and h4 for 2 days. Cell culture images were taken on day 2. Proliferation was measured by ^{3}H-thymidine incorporation. Means ± SEM, n = 5, data representative of three experiments. Scale bar, 0.5 mm.

(C) Purified human B cells were incubated with 2 μg/mL CD40L in the presence or absence of 5 μg/mL 341G2 h1 or h4 for 2 days. Cell culture images were taken on day 2. Proliferation was measured by ^{3}H-thymidine incorporation. Means ± SEM, n = 3–5, data representative of three donors. Scale bar, 0.5 mm.

(D) hCD40Tg mice received 500 μg OVA and 100 μg anti-CD40 mAbs on day 0 and another dose of 100 μg anti-CD40 mAbs on day 3. Mice were bled on day 18 and serum levels of anti-OVA IgG were quantified by ELISA as described in the STAR Methods. Means ± SEM, n = 4–5, data representative of two experiments. Each dot represents one mouse.

(E) Mice received the same treatment as in (D). The level of circulating $CD19^+$ B cells in blood on day 2 was quantified by anti-mouse CD19-APC and expressed as the percentage of $CD45.2^+$ cells. Means ± SEM, n = 4–5, data representative of two experiments. Each dot represents one mouse. Two-tailed, non-paired Student's t test, *p < 0.05, **p < 0.01, ***p < 0.001. n.s., not significant.

induce B cell proliferation at a range of concentrations, consistent with its antagonistic epitope; however, isotype switching to hIgG2 led to profound proliferation and homotypic cell-cell adhesion in hCD40Tg splenic B cells and purified human B cells (Figures 3A and 3B). A time course showed that 341G2 hIgG2-mediated proliferation was extremely rapid, with proliferation detectable as soon as 1 day after treatment and reaching a maximum on day 2 (Figure 3C). In contrast, CP870,893 (also hIgG2), reached maximal activity on day 4 and induced significantly less proliferation (Figure 3C). To enable the comparison of 341G2 hIgG2 activity with other clinically relevant anti-CD40 agonists, we generated the hIgG1 and hIgG2 variants of ADC1013, APX005M, CP870,893, ChiLob 7/4, and SGN40, and showed that 341G2 hIgG2 induced by far the most proliferation, similar to a trivalent CD40L (Figures 3D, S1A, and S1B). Its powerful agonism was further supported by its ability to trigger strong nuclear factor κB (NF-κB) signaling (Figure S1C) in the absence of any FcγR interactions, which are lacking in this system. To further probe the underlying molecular mechanism of such hIgG2-mediated, FcγR-independent agonism, we

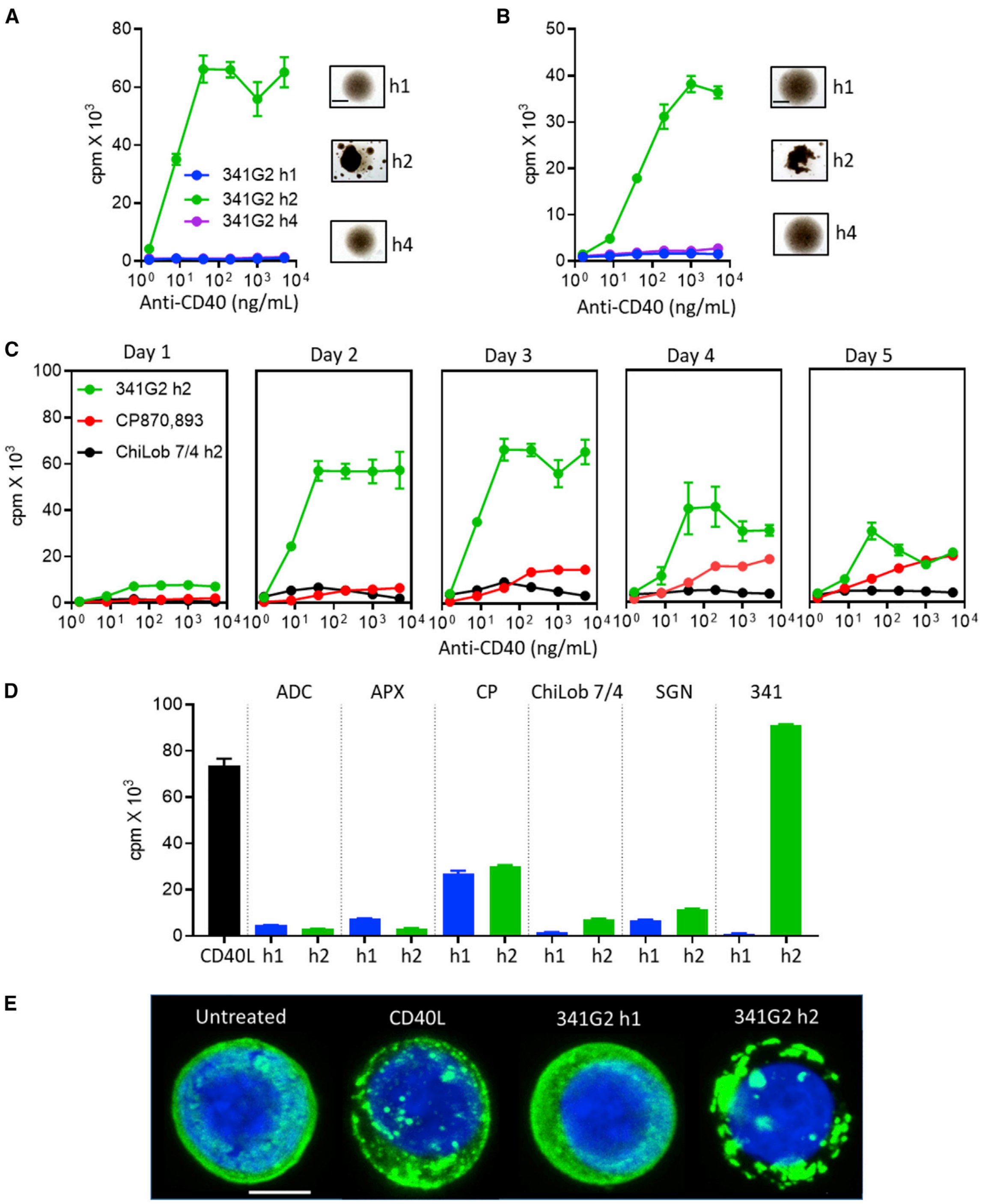

Figure 3. 341G2 hIgG2 (h2) Is a Super-agonist *In Vitro*

(A) Purified hCD40Tg mouse splenic B cells were incubated with various concentrations of 341G2 h1, h2, and h4 for 2 days. Cell culture images were taken on day 2. Proliferation was measured by ^{3}H-thymidine incorporation. Means ± SEM, n = 3, data representative of three experiments. Scale bar, 0.5 mm.

(B) Purified human B cell proliferation assay performed the same as in (A). Means ± SEM, n = 3, data representative of three donors. Scale bar, 0.5 mm.

(C) Purified hCD40Tg mouse splenic B cells were incubated with anti-CD40 mAbs for various periods of time as indicated above each plot. Proliferation was measured by ^{3}H-thymidine incorporation. Means ± SEM, n = 3, data representative of three experiments.

(D) Purified hCD40Tg mouse splenic B cells were incubated with 2 μg/mL clinical anti-CD40 mAbs for 3 days as indicated above each plot. Proliferation was measured by ^{3}H-thymidine incorporation. Means ± SEM, n = 4, data representative of three experiments.

(legend continued on next page)

examined mAb-mediated CD40 clustering of a cell line expressing GFP-conjugated CD40. As shown in Figure 3E, the antagonistic 341G2 hIgG1 caused no significant changes in CD40 clustering compared with the untreated control; in contrast, 341G2 hIgG2 induced significant clustering akin to that delivered by CD40L, indicating that hIgG2 converts antagonists to agonists by promoting receptor clustering. Furthermore, confocal analysis suggested that clusters remained proximal to the plasma membrane, even after extended periods of incubation (Figures S1D and S1E). The lack of apparent internalization was supported by *in-vitro*-quenching assays using both human and hCD40Tg mouse B cells, which demonstrate minimal differences in the level of CD40 detected at 37°C and 4°C, a condition known to reduce receptor internalization (Figure S1F).

To assess *in vivo* activity, we used an OTI $CD8^+$ T cell expansion assay (White et al., 2011). Consistent with *in vitro* data, 341G2 hIgG1 was unable to expand OTI cells *in vivo*, whereas 341G2 hIgG2 led to significant expansion of the adoptively transferred cells, approximately 4-fold higher than CP870,893 (Figure 4A). Importantly, mice tolerated the delivery of this more active mAb. To assess potential toxicity, we assessed weight loss of mice after mAb treatment. We found that both 341G2 hIgG2 and CP870,893-mIgG1, the isotype which is the most agonistic in the mouse (Yu et al., 2018), induced similar levels of agonistic activity and toxicity (Figure S2A). To better recapitulate the human FcγR system, we also investigated the toxicity of 341G2 hIgG2 in hCD40Tg/m*Fcgr2b*$^{-/-}$/h*Fcgr2b*$^{+/-}$ mice that express both hCD40 and hFcγRIIB. Using these mice, we compared the toxicity of 341G2 hIgG2 with APX005M, another strong anti-CD40 agonist observed in the clinic (O'Hara et al., 2019). 341G2 hIgG2 mediated stronger agonism than APX005M but induced no greater toxicity, demonstrating the possibility to separate agonism and toxicity and the potential therapeutic utility of 341G2 hIgG2 (Figure S2B). To evaluate potential cytokine release syndrome (CRS) effects we assayed for typical cytokine markers after anti-CD40 treatment. Consistent with clinical experience (Irenaeus et al., 2019; Vonderheide et al., 2007), agonistic anti-CD40 treatment transiently increased serum interleukin-6 (IL-6), TNF-α, and interferon γ (IFN-γ) levels which returned to baseline after 48 h (Figure S2C). Interestingly, CP870,893-mIgG1 induced higher levels of inflammatory cytokines than 341G2 and CP870,893 hIgG2 at these times, demonstrating the impact of isotype on CRS-based toxicity.

To assess the requirement of FcγR for *in vivo* function, we generated hCD40Tg mice selectively deficient in FcγRIIB (hCD40Tg/*Fcgr2b*$^{-/-}$) or lacking all FcγRs (hCD40Tg/FcγRnull) (Fransen et al., 2018). The levels of OTI expansion induced by 341G2 hIgG2 were similar between hCD40Tg, hCD40Tg/*Fcgr2b*$^{-/-}$, and hCD40Tg/FcγRnull mice (Figure 4B), supporting the notion that the agonistic activity of 341G2 hIgG2 *in vivo* was independent of FcγR. Such FcγR-independent activity was further supported by the ability of 341G2 hIgG2-N297Q, an aglycosylated variant that exhibits significantly reduced affinity for all FcγR (Lux et al., 2013), and 341G2 hIgG2-V234A/G237A/P238S/H268A/V309L/A330S/P331S (c4d), an Fc mutant known to have almost no interaction for all FcγR (Vafa et al., 2014), to induce similar levels of B cell proliferation as the wild-type 341G2 hIgG2 *in vitro* (Figure 4C).

To further dissect the mechanism of this hIgG2-mediated, FcγR-independent, super-agonism, we examined the requirement for the hIgG2 hinge. The hIgG2 CH1 and hinge contain two additional cysteines that are absent in hIgG1 and crucial for the FcγR-independent activity of agonistic anti-CD40 mAbs via differential disulfide bonding (White et al., 2015). Consistent with previous reports, the ability of 341G2 hIgG2 to induce *in vitro* B cell proliferation and *in vivo* OTI expansion was lost when the CH1 and hinge domain of hIgG2 were replaced with those of hIgG1 (hinge 1/2) but not when the CH2 and CH3 domains in hIgG2 were replaced with those from hIgG1 (hinge 2/1) (Figures 4D and 4E). Differential disulfide bonding is also known to give rise to A and B isoforms which differ in their conformation (White et al., 2015). We generated recombinant locked A (C232S/C233S) and B (C127S) forms of 341G2 hIgG2 via selective mutagenesis of key cysteine residues and found that, consistent with our previous findings, only the B form retained significant agonistic activity (Figures 4F and 4G).

As the hIgG2 isotype has previously been shown to induce cytotoxicity *in vivo* (Lux et al., 2014), we assessed the impact of 341G2 hIgG2 on the B cell compartment, a major CD40-expressing immune cell population prone to antibody-mediated depletion. Both 341G2 hIgG2 and its locked B form evoked a significant reduction in the proportion of circulating B cells in blood and concomitant increase in splenic B cells in both hCD40Tg and hCD40Tg/FcγRnull mice (Figures 4H and 4I), indicating FcγR-independent, CD40 activation-mediated B cell re-localization (as opposed to deletion), supported by the upregulation of CD23 on splenic B cells *in vivo* (Figure 4I).

341G2 Human IgG2 Activates Dendritic Cells

Agonistic anti-CD40 mAbs are thought to enhance antigen-specific T cell responses via DC stimulation (Ma and Clark, 2009). To confirm that the 341G2 hIgG2-induced $CD8^+$ T cell expansion *in vivo* was driven by DC activation, we analyzed the splenic $CD11c^+CD8^+DEC205^+$ DC subset known to be crucial for antigen cross-presentation (den Haan et al., 2000; Pooley et al., 2001) (Figure S3A). Consistent with their ability to drive $CD8^+$ T cell expansion, both 341G2 hIgG2 and its locked B form induced significant upregulation of costimulatory molecules CD80 and CD86 in both hCD40Tg and hCD40Tg/FcγRnull mice, while hIgG1 and the locked hIgG2 A form were inert (Figures 5A and 5B). Moreover, anti-CD40 treatment did not significantly alter the frequency of this DC population (Figure 5B), indicating that they were not deleted. To evaluate the agonistic potential of 341G2 hIgG2 on human DCs, we generated monocyte-derived immature DCs and showed that treatment with 341G2 hIgG2 or 341G2 hIgG2-N297Q significantly upregulated costimulatory molecules CD86, CD70, and CD80, whereas 341G2 hIgG1 was inert (Figures 5C and S3B). Moreover,

(E) Jurkat cells stably transfected with human CD40EC-GFP were treated with 10 μg/mL anti-CD40 mAbs as indicated for 1 h at 37°C. Cells were then fixed, nuclear-stained using DAPI, and imaged using a Leica SP8 confocal microscope. z stack images shown. Blue, nucleus; green, human CD40-GFP. Scale bar, 4 μm. Image representative of at least ten images taken.
See also Figure S1.

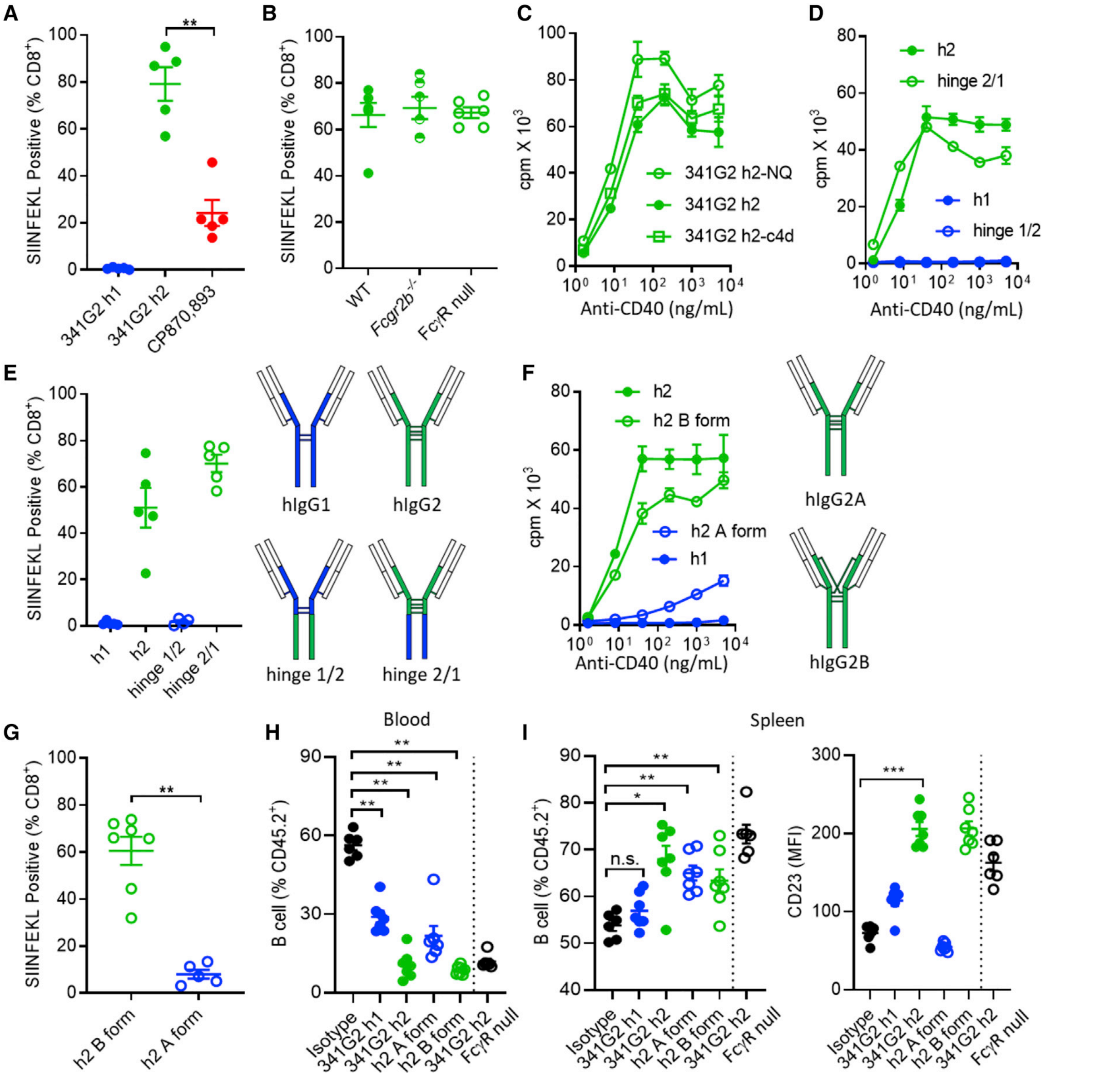

Figure 4. 341G2 h2 Mediates Super-agonistic Activity *In Vivo*

(A) OTI cells (1×10^5) were adoptively transferred into hCD40Tg mice 1 day before treatment with 30 μg anti-CD40 mAbs as indicated. Mice were bled on day 5 and SIINFEKL$^+$ cells were expressed as a percentage of total CD8$^+$ T cells. Means ± SEM, n = 5, data representative of three experiments. Each dot represents one mouse. Two-tailed, non-paired Student's t test, *p < 0.05, **p < 0.01, ***p < 0.001.

(B) OTI expansion assay performed the same as in (A) in hCD40Tg, hCD40Tg/*Fcgr2b*$^{-/-}$, and hCD40Tg/FcγRnull mice. Means ± SEM, n = 5, data representative of two experiments. Each dot represents one mouse.

(C) Purified hCD40Tg mouse splenic B cells were incubated with various concentrations of 341G2 h2 Fc mutants as indicated for 2 days. Proliferation was measured by ^{3}H-thymidine incorporation. Means ± SEM, n = 3, data representative of three experiments.

(D) Purified hCD40Tg mouse splenic B cells were incubated with various concentrations of 341G2 Fc hinge swapped variants as indicated for 2 days. Proliferation was measured by ^{3}H-thymidine incorporation. Means ± SEM, n = 3, data representative of three experiments.

(E) OTI expansion assay performed the same as in (A); mice were treated with 341G2 Fc hinge swapped variants. Means ± SEM, n = 5, data representative of two experiments. Each dot represents one mouse.

(F) Purified hCD40Tg mouse splenic B cells were incubated with various concentrations of locked 341G2 h2 A and B forms for 2 days. Proliferation was measured by ^{3}H-thymidine incorporation. Means ± SEM, n = 3, data representative of three experiments.

(G) OTI expansion assay performed the same as in (A); mice were treated with locked 341G2 h2 A and B forms. Means ± SEM, n = 5–7, data representative of two experiments. Each dot represents one mouse. Two-tailed, non-paired Student's t test, *p < 0.05, **p < 0.01, ***p < 0.001.

(H) hCD40Tg and hCD40Tg/FcγRnull mice received 30 μg anti-CD40 mAbs on day 0 and were bled on day 2. The level of circulating CD19$^+$ B cells in peripheral blood on day 2 was quantified by anti-mouse CD19-APC and expressed as the percentage of CD45.2$^+$ cells. Means ± SEM, n = 6–7, data pooled from two experiments. Each dot represents one mouse. Two-tailed, non-paired Student's t test, *p < 0.05, **p < 0.01, ***p < 0.001.

(legend continued on next page)

341G2 hIgG2 induced a significant production of pro-inflammatory TNF-α and IP-10 (Figure S3C). Interestingly, a lack of Fc effector function conferred by N297Q mutation led to higher levels of TNF-α, IL-12p70, and IFN-γ (Figure S3C). Furthermore, mixed leukocyte reaction assays showed that DCs treated with 341G2 hIgG2 mediated robust allogeneic T cell proliferation akin to the TLR4 agonist LPS (Figure 5D).

Antagonist-Turned Super-agonist Exhibits Antitumor Activity

Next, we examined the antitumor activity of the antagonist-turned super-agonist in three different solid tumor models: MC38 colon carcinoma, EG7 thymoma, and TC1 lung carcinoma. Mice with established MC38 tumors were treated with either the antagonistic 341G2 hIgG1 or agonistic 341G2 hIgG2. Consistent with its antagonistic nature, 341G2 hIgG1 conferred no therapeutic benefit compared with the control, whereas 341G2 hIgG2 significantly improved tumor control (Figure 6A), although it did not lead to appreciable numbers of long-term survivors (Figure 6A).

Given the limited efficacy of single-agent anti-CD40 mAbs in both preclinical models and clinical trials (Hoves et al., 2018; Perry et al., 2018; Vonderheide, 2019; Wiehagen et al., 2017), we explored different therapeutic strategies in an attempt to improve the long-term survival benefit. 341G2 hIgG2 was combined with anti-PD-L1 to remove inhibitory T cell signaling or with the immune-stimulatory mAb anti-CD27 to augment activatory T cell signaling. As expected, anti-PD-L1 monotherapy imparted some modest antitumor activity; however, combination with 341G2 hIgG2 did not result in any synergy (Figure 6B). More encouragingly, while anti-CD27 mAb monotherapy conferred modest survival benefit, combination with 341G2 hIgG2 demonstrated a trend toward improved long-term survival (Figure 6C).

To further explore anti-CD40 mAb therapy for effective cancer treatment, we investigated the potentiating effect of 341G2 hIgG2 on adoptive T cell transfer that has demonstrated clinical efficacy in some difficult-to-treat cancers (Maude et al., 2018; Neelapu et al., 2017). Mice with established OVA-expressing EG7 tumors received adoptively transferred OTI cells and were then treated with anti-CD40 mAbs and OVA. The robust OTI expansion in response to 341G2 hIgG2 treatment was retained in those tumor-bearing mice (Figure 6D). Consistently, while 341G2 hIgG1 did not impart increased therapeutic efficacy to this modality, 341G2 hIgG2 significantly delayed tumor growth and led to long-term survival in almost 100% of mice (Figure 6E).

As adoptive cell transfer may not be amenable for large-scale treatment, we also explored the efficacy of 341G2 hIgG2 in a vaccination setting. The TC1 tumor cell line expresses the HPV-16 E6 and E7 oncogenes (Lin et al., 1996), and a long HPV-16 peptide vaccine has led some vulvar intraepithelial neoplasia patients to exhibit complete responses in the clinic (Kenter et al., 2009). Mice were inoculated with TC1 tumor cells and then vaccinated with 341G2 hIgG2 and the peptide on day 5. Similar to the EG7 model, 341G2 hIgG2 led to a significant reduction in tumor size and 100% long-term survival, superior to CP870,893 (Figure 7A). Importantly, the same potent antitumor activity was recapitulated in hCD40Tg/FcγRnull mice, supporting the Fc-independent activity of 341G2 hIgG2 (Figure 7B). The potent antitumor activity was supported by the induction of a robust endogenous tumor-specific CD8 T cell response (Figure 7C). The same therapeutic efficacy was recapitulated when a lower dose of the peptide vaccine was used (Figure S4).

hIgG2-Mediated Antagonist-to-Agonist Conversion Is Generally Applicable

As all clinical anti-CD40 antagonists are either hIgG1 or hIgG4, we investigated whether the ability to convert antagonists into agonists by isotype switching to hIgG2 is a general phenomenon. We chose CFZ533 (iscalimab, HCD122), currently being investigated clinically as an Fc-silent hIgG1 for Graves' hyperthyroidism, primary Sjogren's syndrome, and prolonging kidney allograft survival. Alanine-scanning mutagenesis mapped the epitope of CFZ533 toward the N terminus of CRD2, overlapping with the CD40L binding site (Figures 8A and 8B), consistent with its antagonistic properties. Similar to 341G2, CFZ533 hIgG2, but not CFZ533 hIgG1, was able to induce CD40 clustering (Figure 8C) and significantly activate the NF-κB signaling pathway (Figure 8D). Both CFZ533 hIgG2 and its aglycosylated variant CFZ533 hIgG2-N297Q comprised A and B isoforms (Figures S5A and S5B) and induced robust proliferation in hCD40Tg and hCD40Tg/*Fcgr2b*$^{-/-}$ mouse splenic B cells, whereas CFZ533 hIgG1 was inactive (Figures 8E and 8F). Moreover, CFZ533 hIgG2 significantly expanded OTI cells *in vivo* (Figure 8G). In addition, we investigated another anti-CD40 antagonist Abbv-323 (ravagalimab) that has undergone clinical testing in Crohn's disease as an Fc-silent hIgG1. Similar to CFZ533, isotype switching to hIgG2 transformed Abbv-323 to an agonist able to trigger CD40 clustering, activate the NF-κB signaling pathway, induce hCD40Tg B cell proliferation, and expand OTI cells *in vivo* (Figures S6A–S6D).

DISCUSSION

The CD40/CD40L axis remains a prime target for immunotherapy. The CD40/CD40L pathway is central to both cellular and humoral adaptive immunity with implications for effective tumor surveillance/control, immune homeostasis, and autoimmunity. Anti-CD40 mAbs that either potentiate or inhibit this pathway therefore possess powerful immune effects and are being explored in clinical trials to treat cancer and autoimmune diseases, respectively. The molecular requirements for effective agonism and antagonism, especially in relation to clinically relevant mAbs, have not been addressed equally. We previously showed that for agonistic anti-CD40 mAbs, both epitope and isotype interplay to govern the level of agonism (Yu et al., 2018). mAbs that target the membrane distal CDR1 display agonistic activity while those that target CRD2 to CRD4 exhibit antagonism (Yu

(I) Mice received the same treatment as in (H). Spleens were harvested on day 2 and analyzed for the level of CD19^{+} B cells expressed as the percentage of CD45.2^{+} cells. CD23 level was quantified by anti-CD23-PE. Means ± SEM, n = 6–7, data pooled from two experiments. Each dot represents one mouse. Two-tailed, non-paired Student's t test, *p < 0.05, **p < 0.01, ***p < 0.001. n.s., not significant.
See also Figure S2.

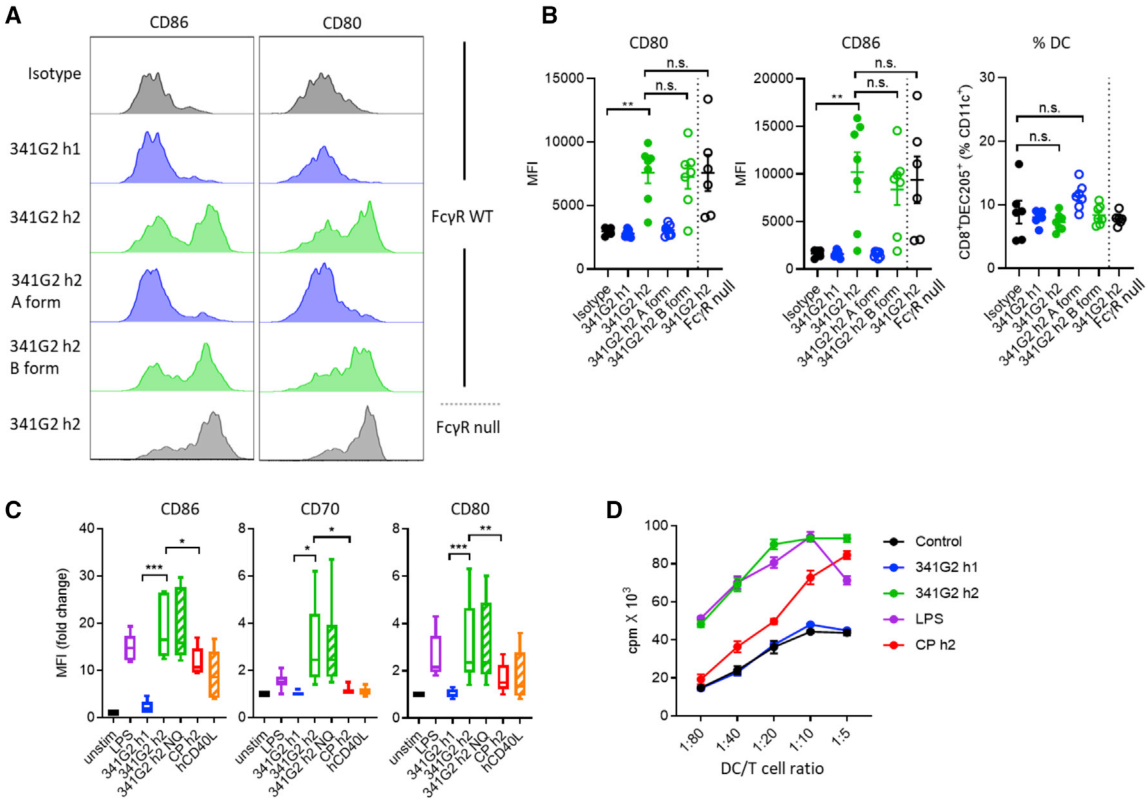

Figure 5. 341G2 h2 Potently Activates Dendritic Cells

(A) hCD40Tg and hCD40Tg/FcγRnull mice received 30 μg anti-CD40 mAbs intravenously and spleens were harvested on day 2. Expression levels of CD80 and CD86 on splenic $CD11c^{+}CD8^{+}DEC205^{+}$ DCs were analyzed by flow cytometry. Histograms representative of six to seven mice from two experiments.

(B) Experiments same as in (A). MFI values for CD80 and CD86 are quantified, and the frequency of $CD11c^{+}CD8^{+}DEC205^{+}$ DC expressed as the percentage of $CD11c^{+}$ cells. Means ± SEM, n = 6–7, data pooled from two experiments. Each dot represents one mouse. Two-tailed, non-paired Student's t test, *$p < 0.05$, **$p < 0.01$, ***$p < 0.001$. n.s., not significant.

(C) Human monocyte-derived DCs were stimulated with various anti-CD40 mAbs, CD40L or LPS as indicated for 48 h and then stained for CD86, CD70, and CD80. The MFI values for each marker in response to treatments were normalized against the MFI values corresponding to the unstimulated control. Means ± SEM, data pooled from six donors. Two-tailed, paired Student's t test, *$p < 0.05$, **$p < 0.01$, ***$p < 0.001$.

(D) Human monocyte-derived DCs pre-treated with anti-CD40 mAbs or LPS for 24 h were washed and then co-cultured with allogeneic $CD4^{+}$ T cells at different ratios as indicated for 5 days. $CD4^{+}$ T cell proliferation was measured by ^{3}H-thymidine incorporation. Means ± SEM, n = 3, data representative of three experiments involving multiple donors.

See also Figure S3.

et al., 2018). This activity is further modulated by both the fine epitope and isotype. Accordingly, binding of certain isotypes to the inhibitory FcγRIIB, expressed on B cells and various myeloid cell populations, was found to be indispensable for the activity of many agonistic anti-CD40 mAbs (Li and Ravetch, 2011; White et al., 2011). It is hypothesized that this activity is delivered through the *trans* engagement between the Fc domain and FcγRIIB stabilizing the Fab-CD40 complex to mimic the interaction between membrane-bound CD40L and CD40 (Beers et al., 2016; Li and Ravetch, 2013). More recently, however, we found that the requirement of the Fc domain for agonism could be obviated by isotype switching to hIgG2 (Yu et al., 2018). hIgG2 uniquely exhibits disulfide bond shuffling at its hinge region giving rise to isoforms that differ in their structural properties (Dillon et al., 2008; Martinez et al., 2008; Wypych et al., 2008). When engrafted onto agonistic anti-CD40 mAbs, hIgG2 was shown to impart enhanced agonistic activity independent of the Fc domain.

In the clinic, agonistic anti-CD40 mAbs possess either wild-type or engineered Fc for enhanced FcγR engagement, whereas antagonistic anti-CD40 mAbs are either hIgG4 or hIgG1 mutants with abrogated Fc effector function (Karnell et al., 2019; Vonderheide, 2019). Antagonists exhibit favorable safety profiles devoid of the CRS typically associated with their agonistic counterparts (Vonderheide, 2019). These clinical experiences affirm the notion that CD40 antagonism is predominantly epitope driven, while antibody-mediated CD40 agonism has both epitope and Fc requirements.

The effect of hIgG2, however, remained unexplored for clinically relevant CD40 antagonists. In this study, we found that isotype switching to hIgG2 converted three antagonists, 341G2, Abbv-323 and CFZ533, all in clinical trials for autoimmunity and transplant rejection, into potent Fc-independent agonists. Such conversion demonstrates that hIgG2 can override the inhibitory effect of antagonistic epitope. Interestingly, APX005M, an agonist in the clinic, was

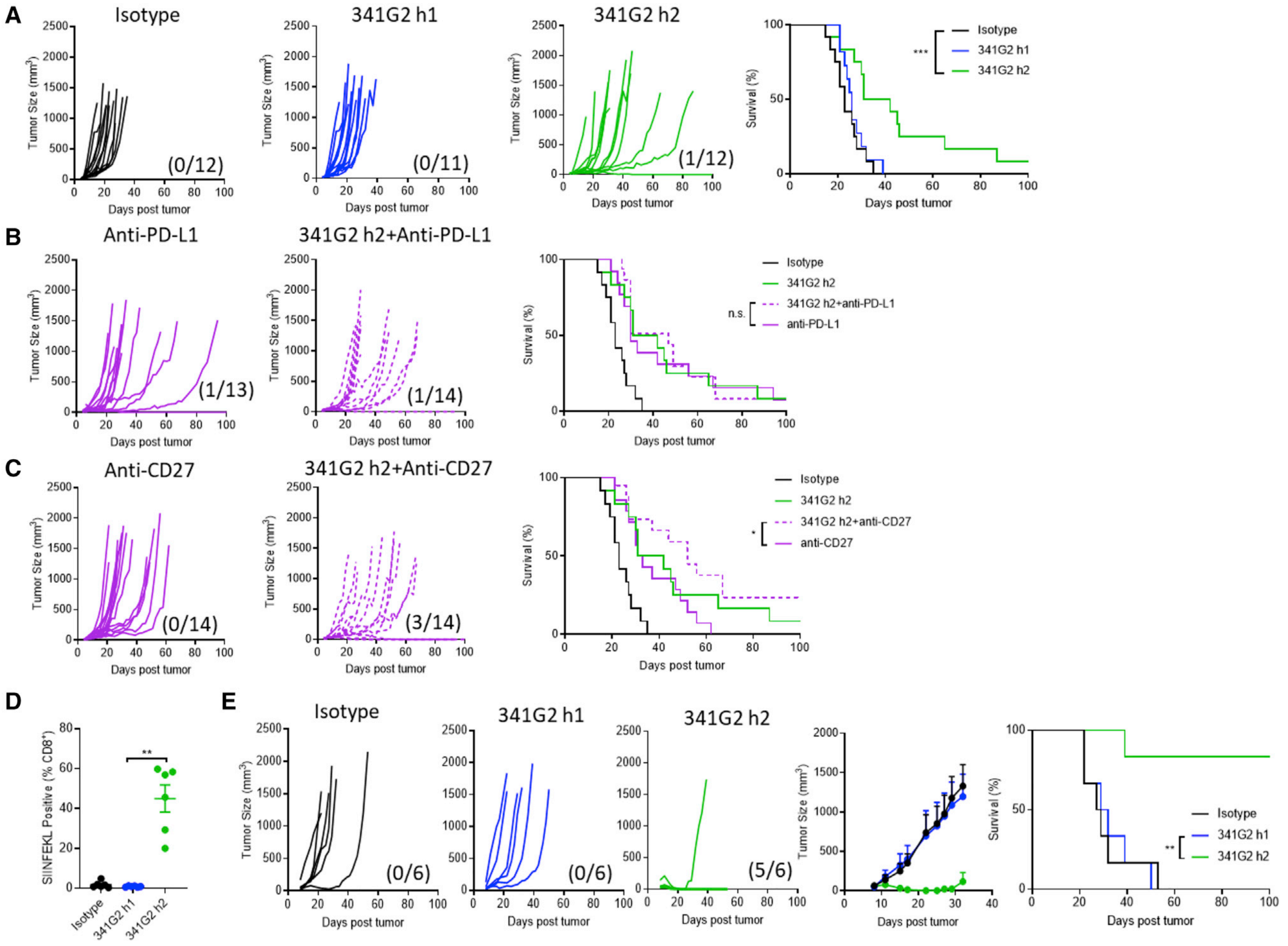

Figure 6. 341G2 h2 Exhibits Antitumor Efficacy and Potentiates Adoptive T Cell Therapy

(A) hCD40Tg mice were inoculated with 5×10^5 MC38 tumor cells subcutaneously. On day 6, when the tumor became palpable, mice were treated with 30 μg anti-CD40 mAbs and again 3 days later. Tumor size and survival were assessed, n = 11–14, data pooled from two experiments. The fractions in parentheses indicate the number of tumor-free mice (numerator) out of the total number of mice (denominator) in that group at the end of the study. Survival curves were compared by log rank test. *$p < 0.05$, **$p < 0.01$, ***$p < 0.001$.

(B) hCD40Tg mice with established MC38 tumors were treated with 30 μg anti-CD40 mAbs in combination with 100 μg anti-PD-L1 mAbs on day 6 and again 3 days later. Tumor size and survival were assessed, n = 11–14, data pooled from two experiments. The fractions in parentheses indicate the number of tumor-free mice (numerator) out of total mice (denominator) in that group at the end of the study. Survival curves were compared by log rank test. *$p < 0.05$, **$p < 0.01$, ***$p < 0.001$.

(C) hCD40Tg mice with established MC38 tumors were treated with 30 μg anti-CD40 in combination with 100 μg anti-CD27 mAbs on day 6 and again 3 days later. Tumor size and survival were assessed, n = 11–14, data pooled from two experiments. The fractions in parentheses indicate the number of tumor-free mice (numerator) out of total number of mice (denominator) in that group at the end of the study. Survival curves were compared by log rank test. *$p < 0.05$, **$p < 0.01$, ***$p < 0.001$.

(D) hCD40Tg mice were inoculated with 5×10^5 EG7 cells subcutaneously. On day 7, when the tumor became palpable, mice received 1×10^5 OTI cells via tail vein injection and 1 day later were treated with 30 μg anti-CD40 mAbs as indicated. Mice were bled 5 days after mAb treatment and SIINFEKL$^+$ cells quantified as a percentage of total CD8$^+$ T cells. Means ± SEM, n = 6, each dot represents one mouse. Data representative of two experiments. Two-tailed, non-paired Student's t test, *$p < 0.05$, **$p < 0.01$, ***$p < 0.001$.

(E) hCD40Tg mice were inoculated with 5×10^5 EG7 cells subcutaneously. On day 7 when the tumor became established, mice received 1×10^5 OTI cells via tail vein injection and 1 day later were treated with 30 μg anti-CD40 mAbs as indicated. Tumor size and survival were assessed, Means ± SEM, n = 6, data representative of two experiments. The fractions in parentheses indicate the number of tumor-free mice (numerator) out of the total number of mice (denominator) in that group at the end of the study. Survival curves were compared by log rank test. *$p < 0.05$, **$p < 0.01$, ***$p < 0.001$.

reported to bind an epitope overlapping with the CD40L binding site and require FcγR for activity (Björck et al., 2016), demonstrating that epitopes that block CD40/CD40L interaction do not necessarily lead to antagonism in the presence of FcγR engagement. Indeed, enhanced crosslinking delivered by FcγRIIB-expressing accessory cells can overcome antagonistic epitopes (Yu et al., 2018) and Fc mutations conferring higher FcγRIIB binding led to improved antitumor activity (Dahan et al., 2016).

As expected, antagonistic 341G2 hIgG1 and hIgG4 inhibited CD40L-mediated B cell activation and the humoral response to OVA immunization. However, both antagonistic

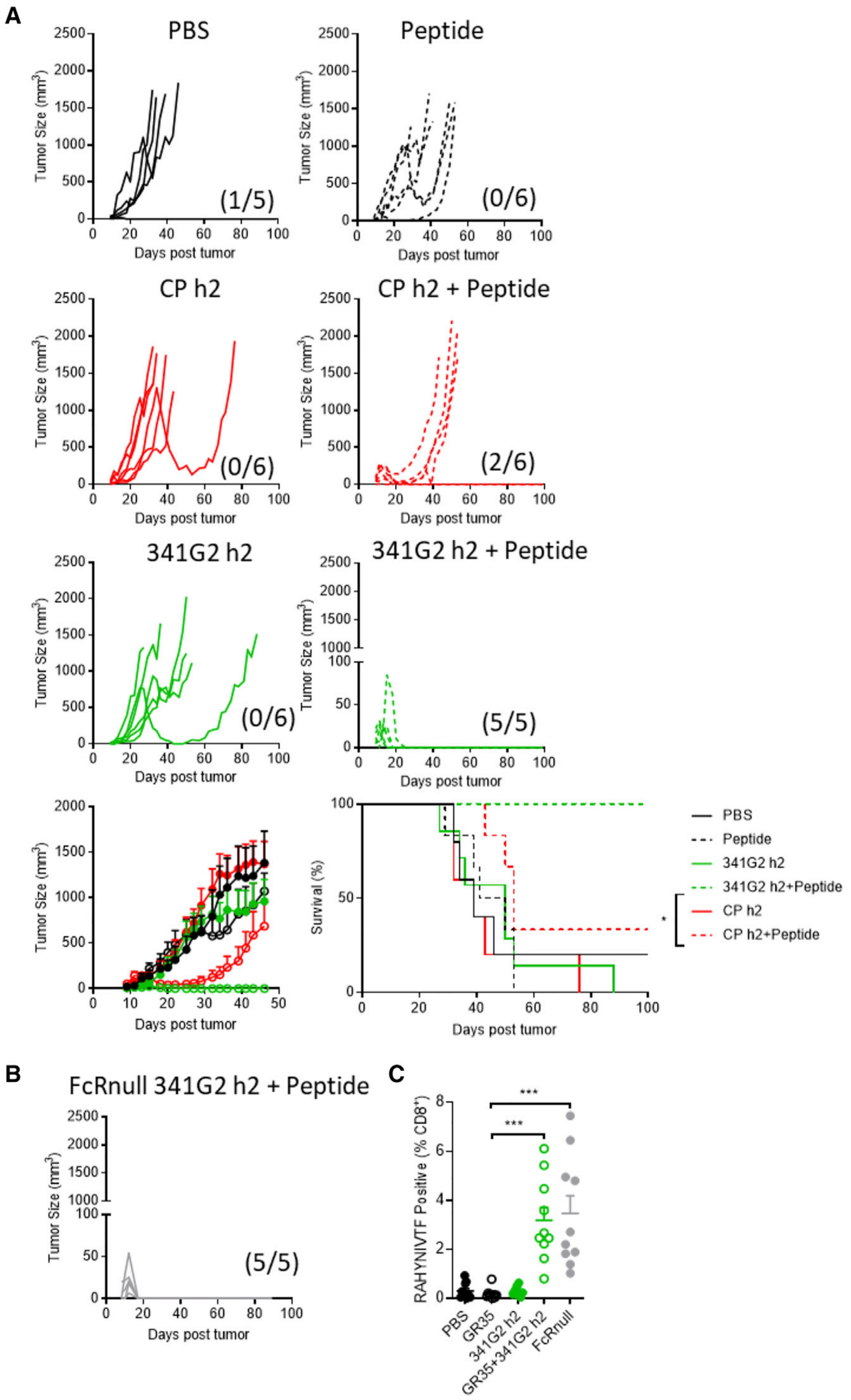

Figure 7. 341G2 h2 Exhibits Antitumor Efficacy in Combination with Peptide Vaccine

(A) hCD40Tg mice were inoculated with 1×10^5 TC1 tumor cells subcutaneously on day 0 and then were treated with 150 μg peptide in combination with 30 μg anti-CD40 on day 5, or treated with 30 μg anti-CD40 alone on days 5, 8, and 11. Tumor size and survival were assessed, Means ± SEM, n = 5–6, data representative of at least two experiments. The fractions in parentheses indicate the number of tumor-free mice (numerator) out of total number of mice (denominator) in that group at the end of the study. Survival curves were compared by log rank test. *p < 0.05, **p < 0.01, ***p < 0.001.

(B) hCD40Tg/FcγRnull mice were inoculated with 1×10^5 TC1 tumor cells subcutaneously on day 0 and then were treated with 150 μg peptide in combination with 30 μg anti-CD40 on day 5. The fraction in parentheses indicates the number of tumor-free mice (numerator) out of the total number of mice (denominator) in that group at the end of the study. Tumor size was quantified. Data representative of at least two experiments.

(C) Mice were treated as in (A) and bled on day 12. RAHYNIVTF$^+$ cells were determined by tetramer staining and expressed as a percentage of total CD8$^+$ T cells. Means ± SEM, n = 9–10, each dot represents one mouse, data pooled from two experiments. Two-tailed, non-paired Student's t test, *p < 0.05, **p < 0.01, ***p < 0.001.

See also Figure S4.

variants also significantly reduced the number of circulating B cells. Such reduction was likely dependent on Fc effector function since deglycosylation of the hIgG1 variant restored the B cell number. hIgG1 was shown previously to interact with all murine FcγRs, including FcγRI and FcγRIV, with higher affinity (Dekkers et al., 2017). As FcγRIV mediates efficient antibody-dependent cellular cytotoxicity (Nimmerjahn et al., 2010; Nimmerjahn and Ravetch, 2006), the opsonization of hCD40Tg B cells by 341G2 hIgG1 could lead to FcγRIV-mediated B cell depletion. In contrast to hIgG1, hIgG4 was shown to interact appreciably only with murine FcγRI (Dekkers et al., 2017), contributing to IgG-mediated cellular cytotoxicity in certain models (Bevaart et al., 2006; Biburger et al., 2011; Bruhns and Jonsson, 2015; Minard-Colin et al., 2008). Accordingly, 341G2 hIgG4 caused a smaller reduction in B cell number compared with hIgG1, presumably due to its interaction with fewer activatory FcγR. The pharmacodynamic effect of 341G2 hIgG4 on B cells in the clinic was not disclosed. In the context of human FcγR known to mediate cellular depletion, hIgG4 engages with the activatory FcγRIIA (131R allelic variant) expressed abundantly on phagocytes (Bruhns and Jonsson, 2015; Dekkers et al., 2017); thus, the parental 341G2 hIgG4 may have a depleting effect on B cells.

Remarkably, 341G2 hIgG2 was found to exhibit four times higher potency than CP870,893 which remains the most agonistic anti-CD40 mAb to be tested clinically to date, capable of delivering some objective partial responses in cancer patients (Vonderheide et al., 2007). However, 341G2 hIgG2 induced no

CellPress
OPEN ACCESS

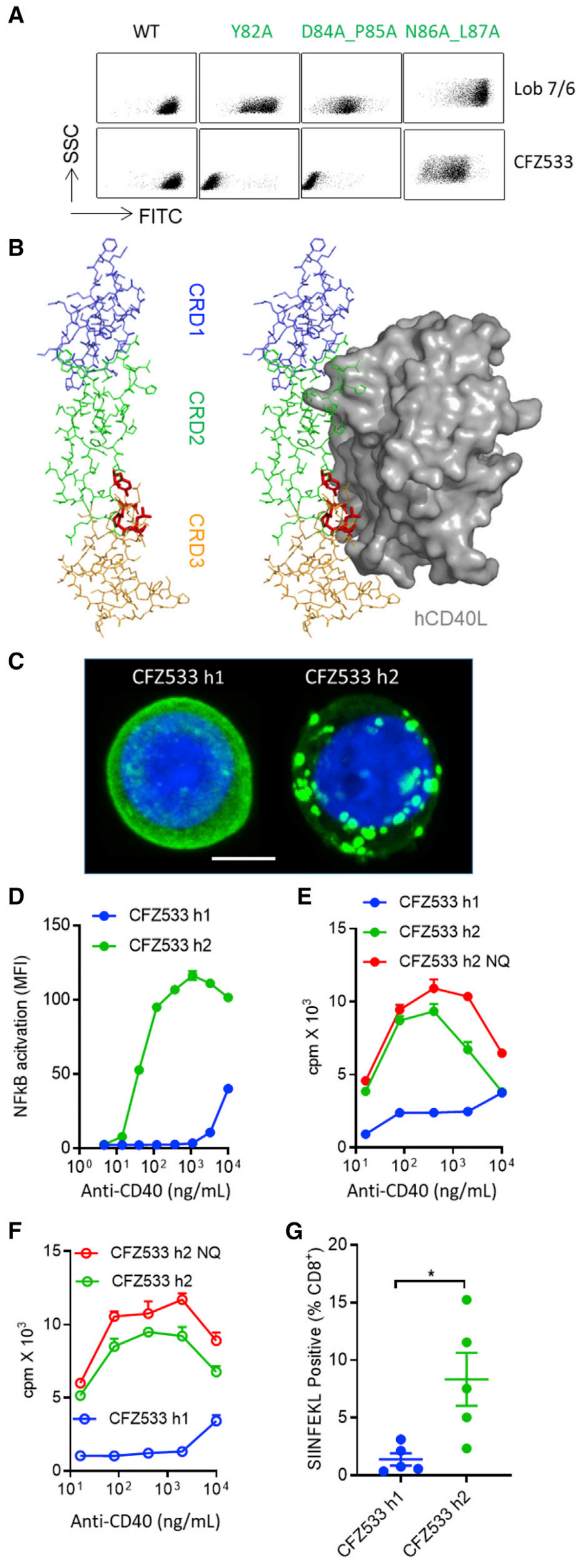

Figure 8. Antagonist CFZ533 Is Converted into an Agonist by Isotype Switching to hIgG2

(A) CHO-k1 cells expressing different hCD40 mutants were probed with anti-CD40 mAbs. Bound mAbs were detected by anti-mouse IgG-FITC.

(B) Deduced epitope of CFZ533 is shown in red on a color-coded CD40 molecular scaffold (left) and displayed relative to the CD40/CD40L binding interface (right). The structure model is based on PDB: 3QD6.

(C) Jurkat cells stably transfected with human CD40EC-GFP were treated with 10 μg/mL CFZ533 h1 or CFZ533 h2 for 1 h at 37°C. Cells were then fixed, nuclear-stained using DAPI, and imaged using a Leica SP8 confocal microscope. z stack images shown. Blue, nucleus; green, human CD40-GFP. Scale bar, 4 μm. Image representative of at least ten images taken.

(D) Jurkat NF-κB GFP reporter cells stably transfected with hCD40 were incubated with various concentrations of anti-CD40 mAbs for 8 h and the level of NF-κB activation was assessed by GFP expression using flow cytometry. Means ± SEM, n = 3, data representative of two experiments.

(E) Purified splenic B cells from hCD40Tg mice were incubated with various concentrations of anti-CD40 mAbs for 4 days. Proliferation was measured by ^{3}H-thymidine incorporation. Means ± SEM, n = 3, data representative of three experiments.

(F) Experiments the same as (E), splenic B cells from hCD40Tg/*Fcgr2b*$^{-/-}$ mice were used. Means ± SEM, n = 3, data representative of three experiments.

(G) 1 × 10^5 OTI cells were adoptively transferred into hCD40Tg mice 1 day before treatment with 100 μg anti-CD40 mAbs as indicated. Mice were bled on day 5 and SIINFEKL$^+$ cells were quantified as a percentage of total CD8$^+$ T cells. Means ± SEM, n = 5, each dot represents one mouse, data representative of two experiments. Two-tailed, non-paired Student's t test, *$p < 0.05$, **$p < 0.01$, ***$p < 0.001$.

See also Figures S5 and S6.

more weight loss than CP870,893 or APX005M, both strong clinically relevant agonists. Moreover, 341G2 hIgG2 induced similar levels of transient inflammatory cytokines as CP870,893-hIgG2 but significantly less than CP870,893-mIgG1, indicating the impact of isotype on toxicity. Our data thus demonstrate the possibility to separate agonism and toxicity and highlight the potential therapeutic utility of 341G2 hIgG2. In addition to CRS, clinical anti-CD40 agonists induce transient B cell depletion from circulation (Irenaeus et al., 2019; Johnson et al., 2015; Vonderheide et al., 2007). Our observation that 341G2 hIgG2 significantly reduced circulating B cells but increased splenic B cell activation and accumulation in both hCD40Tg and hCD40Tg/FcγRnull mice suggests the possibility that these CD40-activated B cells re-localize from systemic circulation to other tissues in an FcγR-independent manner.

While the mechanism behind the super-agonistic 341G2 hIgG2 remains unclear, alanine-scanning mutagenesis and SEC-SAXS followed by docking analysis revealed that 341G2 engages both CRD1 and CRD2 domains, which, to our knowledge is unique among anti-CD40 mAbs characterized to date that only engage a single CRD. It is possible that a rigid hIgG2 hinge conferred by differential disulfide bonding combined with this dual-CRD targeting induces greater CD40 conformational changes amenable to receptor clustering and activation. Indeed, our finding that 341G2 hIgG2 induced significant CD40 clustering in a system that lacks all FcγR supports this theory. Moreover, orthogonal viewing of our confocal images and quenching assays indicate that the majority of those clusters remained proximal to the cell surface as opposed to being internalized in those cells we studied. These data are consistent with the previous observation that antibody-mediated CD40 internalization is less efficient than other receptors, including DEC205 and the mannose receptor (Chatterjee et al., 2012) as well as CD20 (Vaughan et al., 2014). Such correlation between receptor clustering and agonistic activity is in accordance with the paradigm of TNF receptor activation and its prerequisite for downstream

receptor signaling (Ward-Kavanagh et al., 2016). Powerful epitope-driven agonism was previously reported for CP870,893, which binds to residues on the CRD1 domain facing away from the CD40L binding site and exhibits similarly high levels of agonistic activity independent of isotype (Yu et al., 2018). Interestingly, 341G2 hIgG2 was previously described to provide synergy with the TLR3 agonist poly(IC:LC) in a viral vaccination setting, although neither the rationale for using hIgG2 isotype nor the antagonistic nature of 341G2 were discussed (Thompson et al., 2015).

The agonistic activity of 341G2 hIgG2 was recapitulated in mice lacking FcγRIIB or all FcγRs, demonstrated by both *in vivo* DC activation and CD8$^+$ T cell expansion, consistent with the previous finding that hIgG2 imparts agonism in an FcγR-independent manner (White et al., 2015). Such FcγR-independent agonism may overcome the challenge of heterogeneous FcγR expression among different tumor microenvironment and inter-patient variability to deliver more consistent agonistic activity. Accordingly, the aglycosylated variant 341G2 hIgG2-N297Q also potently activated B cells and human DCs. Interestingly, at the cytokine level, the absence of Fc effector function conferred by the N297Q mutation induced significantly higher levels of TNF-α, IL-12p70, and IFN-γ in human DCs, which suggests that Fc-FcγR engagement on DCs may negatively regulate CD40-mediated activation. Human monocyte-derived DCs express predominantly FcγRIIA and FcγRIIB; moreover, FcγRIIB-mediated DC suppression has been described before (Boruchov et al., 2005; Dhodapkar et al., 2007). FcγRIIB present on DCs (in *cis* or in *trans*) could potentially influence CD40 activation through the ITIM signaling motif (Pauls and Marshall, 2017). Although hIgG2 does not engage FcγRIIB in solution, interactions could occur when the FcγRIIB density reaches a critical level, such as when FcγRIIB-expressing cells are used in assays to provide Fc crosslinking (Dudek et al., 2019; White et al., 2011).

In addition to the direct immunomodulatory effect, we also investigated the antitumor activity of the antagonist-turned agonist. 341G2 hIgG2 monotherapy significantly improved the survival rate of tumor-bearing mice compared with its antagonistic hIgG1 counterpart, confirming its agonistic conversion. However, there was a lack of long-term survivors despite treatment with the maximum tolerated dose. This mirrors clinical experiences of single-agent agonistic anti-CD40 mAbs, whereby the maximum tolerated dose induced significant adverse events in patients but failed to achieve numerous objective responses (Vonderheide, 2019). One hypothesis for the lack of clinical activity points to the low mAb dose which would fail to reach systemic penetrance and maximal receptor occupancy. In support of this argument, bispecific constructs that guide anti-CD40 to the tumor microenvironment were developed to minimize off-target toxicity and have showed some preclinical efficacy (Rigamonti et al., 2019; Ye et al., 2019). In the clinic, however, similar levels of antitumor response, albeit low, were observed across all anti-CD40 mAb doses tested (Vonderheide, 2019). Moreover, B cell and DC activation were consistently detected in differentially dosed patient groups across multiple independent clinical trials (Vonderheide, 2019). Overall, agonistic anti-CD40 mAbs in the clinic induced significant immune activation but failed to demonstrate meaningful antitumor activity, which could be due to endogenous compensatory mechanisms in place to prevent immune over-activation. For example, preclinical studies suggest that agonistic anti-CD40 mAbs induce an antigen-specific T cell response but fail to sustain it beyond 1 month and instead lead to accelerated T cell deletion (Kedl et al., 2001). Furthermore, immune cell activation by agonistic anti-CD40 mAbs was accompanied by significant upregulation of inhibitory receptors which could dampen the immune response (Ngiow et al., 2016; Wiehagen et al., 2017).

To overcome the limited single-agent efficacy, various anti-CD40 combination therapies, mainly with checkpoint inhibitors and chemotherapy, have entered the clinic. Encouragingly, in a recent trial, the agonistic anti-CD40 mAb, APX005M, in combination with chemotherapy and/or anti-PD-1 mAbs, achieved over 50% overall response rate in metastatic ductal pancreatic adenocarcinoma patients (O'Hara et al., 2019). In our solid tumor model, PD-L1 blockade alone exhibited some modest activity; however, combination with 341G2 hIgG2 did not lead to improved survival. One possible reason for the discrepancy is that the anti-PD-L1 mAb used in our study (10F.9G2) was a rat IgG2b isotype that is known to interact with all mouse FcγRs with a high A/I ratio (Dahan et al., 2016). As PD-L1 expression is upregulated on activated CD8$^+$ T cells upon anti-CD40 treatment *in vivo*, the engagement of anti-PD-L1 with FcγRs could lead to selective deletion of effector T cells, hence masking the true potential of this combination regimen. Encouragingly, we observed some synergy between 341G2 hIgG2 and an agonistic anti-CD27 mAbs in improving long-term survival, and future experiments will focus on mechanistic dissection of this synergy to inform on better clinical strategy (Buchan et al., 2018). In addition to combination therapy, the route of administration could influence the clinical utility of anti-CD40 mAbs. For example, it was demonstrated that intratumoral, rather than systemic, delivery of anti-CD40 mAbs led to enhanced antitumor activity with reduced toxicity (Knorr et al., 2018).

Besides antibody therapy, the CD40/CD40L axis has been exploited to improve the response rate of chimeric antigen receptor (CAR) T cell therapy in solid tumors. The ectopic expression of CD40L in CAR T cells has been shown to potentiate endogenous tumor-specific T cell responses (Kuhn et al., 2019). Moreover, the introduction of the CD40 signaling domain into CAR T cells led to superior T cell effector function (Mata et al., 2017). Consistent with these reports, our data show that 341G2 hIgG2 enabled adoptively transferred T cells to achieve long-term survival in a solid tumor model. A similarly impressive synergistic therapeutic effect was achieved in a peptide vaccination setting—with 341G2 hIgG2 exhibiting greater efficacy than CP870,893.

In summary, we demonstrate that antagonistic anti-CD40 mAbs can be converted into potent FcγR-independent agonists by isotype switching to hIgG2, in a manner that is dependent upon the hIgG2 hinge. The success of agonistic anti-CD40 mAbs in oncology will likely involve combination with other agents and we provide data in support of possible approaches for subsequent development. Further investigation into immunological function and dysfunction associated with anti-CD40 mAbs will help inform on the rational combinations to advance to the clinic.

Beyond CD40, isotype switching to hIgG2 has been demonstrated to enhance the agonistic activity of mAbs targeting other

TNF receptors, such as 41BB, as well as members of the B7-CD28 superfamily (White et al., 2015), and so similar antagonism to agonism conversion remains a possibility for these other specificities. The hIgG2 isotype has previously been used as a more Fc-"inert" IgG molecule with reduced effector functions due to its restricted C1q and FcγR-binding properties (Vidarsson et al., 2014). However, the transformative property of the hIgG2 isotype described in this study, converting antagonists to potential super-agonists, heeds caution for the use of this isotype in the development of these reagents where pure blocking and antagonism are desired.

STAR★METHODS

Detailed methods are provided in the online version of this paper and include the following:

- KEY RESOURCES TABLE
- RESOURCE AVAILABILITY
  - Lead Contact
  - Materials Availability
  - Data and Code Availability
- EXPERIMENTAL MODEL AND SUBJECT DETAILS
  - Mice
  - Human Samples
  - Cell Lines
- METHOD DETAILS
  - Antibodies and Reagents
  - Generation of CD40 Constructs and Epitope Mapping
  - Confocal Microscopy
  - *In Vitro* Receptor Internalization Assay
  - Surface Plasmon Resonance
  - Western Blot
  - Size Exclusion Chromatography Coupled with Small-Angle X-Ray Scattering (SEC-SAXS)
  - Homology Modelling and Docking
  - NFκB Assay
  - B Cell Activation and Proliferation
  - Human Dendritic Cell Activation and Mixed Leukocyte Reaction
  - *In Vivo* Assessment of Agonistic and Antagonistic Activity
  - Serum Cytokine Analysis
  - Tumor Models and Immunotherapy Treatment
- QUANTIFICATION AND STATISTICAL ANALYSIS

SUPPLEMENTAL INFORMATION

Supplemental Information can be found online at https://doi.org/10.1016/j.ccell.2020.04.013.

ACKNOWLEDGMENTS

We thank the members of the Antibody and Vaccine group for useful discussions and the preclinical unit staff for animal husbandry. We thank Diamond Light Source for beam time on B21 (ref. SM21035-186) and to Nikul Khunti for his support. We thank David Johnston from the Biomedical Imaging Unit, Southampton General Hospital, Southampton, UK, for assistance with Confocal. Funding was provided by Cancer Research UK grants A10834, A20537, A18087, A25139, and A25169, as well as European Union FP7 grant 602262-2.

AUTHOR CONTRIBUTIONS

X.Y. designed and performed the experiments, analyzed and interpreted data, and wrote the manuscript. H.T.C.C., C.A.P., J.K., H.F., T.I., C.I.M., R.R.F., P.J.D., L.R.D., V.E., J.S.V., A.L.W., and I.T. generated or provided key reagents or performed and analyzed the research. M.J.G. designed the study, supervised data collection, discussed and interpreted data, and edited the manuscript. M.S.C. designed the study, supervised data collection, discussed and interpreted data, and wrote the manuscript with X.Y. M.J.G. and M.S.C. were co-senior authors on the study.

DECLARATION OF INTERESTS

M.S.C. acts as a consultant for a number of biotech companies, being retained as a consultant for BioInvent and has received research funding from BioInvent, GSK, UCB, iTeos, and Roche. M.J.G. acts as a consultant to a number of biotech companies and receives institutional payments and royalties from antibody patents and licenses. This work is related to patent Family WO 2015/145360 protecting antibodies containing modified hIgG2 domains which elicit agonist or antagonistic properties.

Received: October 28, 2019
Revised: February 28, 2020
Accepted: April 21, 2020
Published: May 21, 2020

REFERENCES

Ahonen, C., Manning, E., Erickson, L.D., O'Connor, B., Lind, E.F., Pullen, S.S., Kehry, M.R., and Noelle, R.J. (2002). The CD40-TRAF6 axis controls affinity maturation and the generation of long-lived plasma cells. Nat. Immunol. *3*, 451–456.

Ambrosetti, F., Jimenez-Garcia, B., Roel-Touris, J., and Bonvin, A. (2020). Modeling antibody-antigen complexes by information-driven docking. Structure *28*, 119–129.e2.

Anil Kumar, M.S., Papp, K., Tainaka, R., Valluri, U., Wang, X., Zhu, T., and Schwabe, C. (2018). Randomized, controlled study of bleselumab (ASKP1240) pharmacokinetics and safety in patients with moderate-to-severe plaque psoriasis. Biopharm. Drug Dispos. *39*, 245–255.

Austin, C.D., De Maziere, A.M., Pisacane, P.I., van Dijk, S.M., Eigenbrot, C., Sliwkowski, M.X., Klumperman, J., and Scheller, R.H. (2004). Endocytosis and sorting of ErbB2 and the site of action of cancer therapeutics trastuzumab and geldanamycin. Mol. Biol. Cell *15*, 5268–5282.

Banchereau, J., Bazan, F., Blanchard, D., Briere, F., Galizzi, J.P., van Kooten, C., Liu, Y.J., Rousset, F., and Saeland, S. (1994). The CD40 antigen and its ligand. Annu. Rev. Immunol. *12*, 881–922.

Beers, S.A., Glennie, M.J., and White, A.L. (2016). Influence of immunoglobulin isotype on therapeutic antibody function. Blood *127*, 1097–1101.

Bennett, S.R., Carbone, F.R., Karamalis, F., Flavell, R.A., Miller, J.F., and Heath, W.R. (1998). Help for cytotoxic-T-cell responses is mediated by CD40 signalling. Nature *393*, 478–480.

Bevaart, L., Jansen, M.J., van Vugt, M.J., Verbeek, J.S., van de Winkel, J.G., and Leusen, J.H. (2006). The high-affinity IgG receptor, FcgammaRI, plays a central role in antibody therapy of experimental melanoma. Cancer Res. *66*, 1261–1264.

Biburger, M., Aschermann, S., Schwab, I., Lux, A., Albert, H., Danzer, H., Woigk, M., Dudziak, D., and Nimmerjahn, F. (2011). Monocyte subsets responsible for immunoglobulin G-dependent effector functions in vivo. Immunity *35*, 932–944.

Björck, P., Filbert, E., Yang, X., and Trifan, O.C. (2016). APX005M, a humanized anti-CD40 antibody with strong immune-modulatory activities capable of tumor eradication in vivo. [abstract]. Cancer Res. *76* (14 Suppl), 5004.

Boruchov, A.M., Heller, G., Veri, M.C., Bonvini, E., Ravetch, J.V., and Young, J.W. (2005). Activating and inhibitory IgG Fc receptors on human DCs mediate opposing functions. J. Clin. Invest. *115*, 2914–2923.

Bruhns, P., and Jonsson, F. (2015). Mouse and human FcR effector functions. Immunol. Rev. *268*, 25–51.

Buchan, S.L., Rogel, A., and Al-Shamkhani, A. (2018). The immunobiology of CD27 and OX40 and their potential as targets for cancer immunotherapy. Blood *131*, 39–48.

Chatterjee, B., Smed-Sorensen, A., Cohn, L., Chalouni, C., Vandlen, R., Lee, B.C., Widger, J., Keler, T., Delamarre, L., and Mellman, I. (2012). Internalization and endosomal degradation of receptor-bound antigens regulate the efficiency of cross presentation by human dendritic cells. Blood *120*, 2011–2020.

Claus, C., Ferrara, C., Xu, W., Sam, J., Lang, S., Uhlenbrock, F., Albrecht, R., Herter, S., Schlenker, R., Husser, T., et al. (2019). Tumor-targeted 4-1BB agonists for combination with T cell bispecific antibodies as off-the-shelf therapy. Sci. Transl. Med. *11*, https://doi.org/10.1126/scitranslmed.aav5989.

Dahan, R., Barnhart, B.C., Li, F., Yamniuk, A.P., Korman, A.J., and Ravetch, J.V. (2016). Therapeutic activity of agonistic, human anti-CD40 monoclonal antibodies requires selective FcgammaR engagement. Cancer Cell *29*, 820–831.

Dekkers, G., Bentlage, A.E.H., Stegmann, T.C., Howie, H.L., Lissenberg-Thunnissen, S., Zimring, J., Rispens, T., and Vidarsson, G. (2017). Affinity of human IgG subclasses to mouse Fc gamma receptors. MAbs *9*, 767–773.

Dhodapkar, K.M., Banerjee, D., Connolly, J., Kukreja, A., Matayeva, E., Veri, M.C., Ravetch, J.V., Steinman, R.M., and Dhodapkar, M.V. (2007). Selective blockade of the inhibitory Fcgamma receptor (FcgammaRIIB) in human dendritic cells and monocytes induces a type I interferon response program. J. Exp. Med. *204*, 1359–1369.

Dillon, T.M., Ricci, M.S., Vezina, C., Flynn, G.C., Liu, Y.D., Rehder, D.S., Plant, M., Henkle, B., Li, Y., Deechongkit, S., et al. (2008). Structural and functional characterization of disulfide isoforms of the human IgG2 subclass. J. Biol. Chem. *283*, 16206–16215.

Dudek, S., Weissmuller, S., Anzaghe, M., Miller, L., Sterr, S., Hoffmann, K., Hengel, H., and Waibler, Z. (2019). Human Fcgamma receptors compete for TGN1412 binding that determines the antibody's effector function. Eur. J. Immunol. *49*, 1117–1126.

Elgueta, R., Benson, M.J., de Vries, V.C., Wasiuk, A., Guo, Y., and Noelle, R.J. (2009). Molecular mechanism and function of CD40/CD40L engagement in the immune system. Immunol. Rev. *229*, 152–172.

Farkash, E., Naik, A., Tedesco-Silva, H., Nashan, B., Witzke, O., van den Hoogen, M., Berger, S., Cibrik, D.M., Mulgaonkar, S., Leeser, D.B., et al. (2019). CNI-free therapy with iscalimab (anti-CD40 mAb) preserves allograft histology compared to standard of care after kidney transplantation [abstract]. Am. J. Transplant. *19* (*Suppl. 3*), 632.

Fisher, B., Zeher, M., Ng, W.F., Bombardieri, M., Posch, M., Papas, A.S., Farag, A.M., Daikeler, T., Bannert, B., Kivitz, A.J., et al. (2017). The novel anti-CD40 monoclonal antibody CFZ533 shows beneficial effects in patients with primary Sjögren's syndrome: a phase IIa double-blind, placebo-controlled randomized trial [abstract]. Arthritis Rheumatol. *69* (*Suppl. 10*), 1784.

Fransen, M.F., Benonisson, H., van Maren, W.W., Sow, H.S., Breukel, C., Linssen, M.M., Claassens, J.W.C., Brouwers, C., van der Kaa, J., Camps, M., et al. (2018). A restricted role for FcgammaR in the regulation of adaptive immunity. J. Immunol. *200*, 2615–2626.

French, R.R., Chan, H.T., Tutt, A.L., and Glennie, M.J. (1999). CD40 antibody evokes a cytotoxic T-cell response that eradicates lymphoma and bypasses T-cell help. Nat. Med. *5*, 548–553.

den Haan, J.M., Lehar, S.M., and Bevan, M.J. (2000). CD8(+) but not CD8(-) dendritic cells cross-prime cytotoxic T cells in vivo. J. Exp. Med. *192*, 1685–1696.

Harland, R.C., Klintmalm, G., Jensik, S., Yang, H., Bromberg, J., Holman, J., Anil Kumar, M., Santos, V., Larson, T., and Wang, X. (2017). Efficacy and safety of bleselumab in kidney transplant recipients: a phase 2, randomized, open-label study. Am. J. Transplant. *17*, 159–171.

Hoves, S., Ooi, C.H., Wolter, C., Sade, H., Bissinger, S., Schmittnaegel, M., Ast, O., Giusti, A.M., Wartha, K., Runza, V., et al. (2018). Rapid activation of tumor-associated macrophages boosts preexisting tumor immunity. J. Exp. Med. *215*, 859–876.

Imai, A., Suzuki, T., Sugitani, A., Itoh, T., Ueki, S., Aoyagi, T., Yamashita, K., Taniguchi, M., Takahashi, N., Miura, T., et al. (2007). A novel fully human anti-CD40 monoclonal antibody, 4D11, for kidney transplantation in cynomolgus monkeys. Transplantation *84*, 1020–1028.

Irenaeus, S.M.M., Nielsen, D., Ellmark, P., Yachnin, J., Deronic, A., Nilsson, A., Norlen, P., Veitonmaki, N., Wennersten, C.S., and Ullenhag, G.J. (2019). First-in-human study with intratumoral administration of a CD40 agonistic antibody, ADC-1013, in advanced solid malignancies. Int. J. Cancer *145*, 1189–1199.

Johnson, P., Challis, R., Chowdhury, F., Gao, Y., Harvey, M., Geldart, T., Kerr, P., Chan, C., Smith, A., Steven, N., et al. (2015). Clinical and biological effects of an agonist anti-CD40 antibody: a Cancer Research UK phase I study. Clin. Cancer Res. *21*, 1321–1328.

Kahaly, G., Stan, M., Gergely, P., Colin, L., Amer, A., Schuhmann, I., Espie, P., Rush, J., Basson, C., and He, Y. (2019). OR19-6 A novel anti-CD40 monoclonal antibody, iscalimab, successfully treats Graves' hyperthyroidism. J. Endocr. Soc. *3*, https://doi.org/10.1210/js.2019-OR19-6.

Karnell, J.L., Rieder, S.A., Ettinger, R., and Kolbeck, R. (2019). Targeting the CD40-CD40L pathway in autoimmune diseases: humoral immunity and beyond. Adv. Drug Deliv. Rev. *141*, 92–103.

Kasran, A., Boon, L., Wortel, C.H., Hogezand, R.A., Schreiber, S., Goldin, E., Boer, M., Geboes, K., Rutgeerts, P., and Ceuppens, J.L. (2005). Safety and tolerability of antagonist anti-human CD40 Mab ch5D12 in patients with moderate to severe Crohn's disease. Aliment. Pharmacol. Ther. *22*, 111–122.

Kedl, R.M., Jordan, M., Potter, T., Kappler, J., Marrack, P., and Dow, S. (2001). CD40 stimulation accelerates deletion of tumor-specific CD8(+) T cells in the absence of tumor-antigen vaccination. Proc. Natl. Acad. Sci. U S A *98*, 10811–10816.

Kelley, L.A., Mezulis, S., Yates, C.M., Wass, M.N., and Sternberg, M.J. (2015). The Phyre2 web portal for protein modeling, prediction and analysis. Nat. Protoc. *10*, 845–858.

Kenter, G.G., Welters, M.J., Valentijn, A.R., Lowik, M.J., Berends-van der Meer, D.M., Vloon, A.P., Essahsah, F., Fathers, L.M., Offringa, R., Drijfhout, J.W., et al. (2009). Vaccination against HPV-16 oncoproteins for vulvar intraepithelial neoplasia. N. Engl. J. Med. *361*, 1838–1847.

Knight, C.J., and Hub, J.S. (2015). WAXSiS: a web server for the calculation of SAXS/WAXS curves based on explicit-solvent molecular dynamics. Nucleic Acids Res. *43*, W225–W230.

Knorr, D.A., Dahan, R., and Ravetch, J.V. (2018). Toxicity of an Fc-engineered anti-CD40 antibody is abrogated by intratumoral injection and results in durable antitumor immunity. Proc. Natl. Acad. Sci. U S A *115*, 11048–11053.

Kuhn, N.F., Purdon, T.J., van Leeuwen, D.G., Lopez, A.V., Curran, K.J., Daniyan, A.F., and Brentjens, R.J. (2019). CD40 ligand-modified chimeric antigen receptor T cells enhance antitumor function by eliciting an endogenous antitumor response. Cancer Cell *35*, 473.

Lai, J.H., Luo, S.F., and Ho, L.J. (2019). Targeting the CD40-CD154 signaling pathway for treatment of autoimmune arthritis. Cells *8*, 927.

Larsen, C.P., and Pearson, T.C. (1997). The CD40 pathway in allograft rejection, acceptance, and tolerance. Curr. Opin. Immunol. *9*, 641–647.

Li, F., and Ravetch, J.V. (2011). Inhibitory Fcgamma receptor engagement drives adjuvant and anti-tumor activities of agonistic CD40 antibodies. Science *333*, 1030–1034.

Li, F., and Ravetch, J.V. (2013). Antitumor activities of agonistic anti-TNFR antibodies require differential FcgammaRIIB coengagement in vivo. Proc. Natl. Acad. Sci. U S A *110*, 19501–19506.

Lievens, D., Eijgelaar, W.J., Biessen, E.A., Daemen, M.J., and Lutgens, E. (2009). The multi-functionality of CD40L and its receptor CD40 in atherosclerosis. Thromb. Haemost. *102*, 206–214.

Lin, K.Y., Guarnieri, F.G., Staveley-O'Carroll, K.F., Levitsky, H.I., August, J.T., Pardoll, D.M., and Wu, T.C. (1996). Treatment of established tumors with a novel vaccine that enhances major histocompatibility class II presentation of tumor antigen. Cancer Res. *56*, 21–26.

CellPress
OPEN ACCESS

Lux, A., Yu, X., Scanlan, C.N., and Nimmerjahn, F. (2013). Impact of immune complex size and glycosylation on IgG binding to human FcgammaRs. J. Immunol. *190*, 4315–4323.

Lux, A., Seeling, M., Baerenwaldt, A., Lehmann, B., Schwab, I., Repp, R., Meidenbauer, N., Mackensen, A., Hartmann, A., Heidkamp, G., et al. (2014). A humanized mouse identifies the bone marrow as a niche with low therapeutic IgG activity. Cell Rep. *7*, 236–248.

Ma, D.Y., and Clark, E.A. (2009). The role of CD40 and CD154/CD40L in dendritic cells. Semin. Immunol. *21*, 265–272.

Martinez, T., Guo, A., Allen, M.J., Han, M., Pace, D., Jones, J., Gillespie, R., Ketchem, R.R., Zhang, Y., and Balland, A. (2008). Disulfide connectivity of human immunoglobulin G2 structural isoforms. Biochemistry *47*, 7496–7508.

Mata, M., Gerken, C., Nguyen, P., Krenciute, G., Spencer, D.M., and Gottschalk, S. (2017). Inducible activation of MyD88 and CD40 in CAR T cells results in controllable and potent antitumor activity in preclinical solid tumor models. Cancer Discov. *7*, 1306–1319.

Maude, S.L., Laetsch, T.W., Buechner, J., Rives, S., Boyer, M., Bittencourt, H., Bader, P., Verneris, M.R., Stefanski, H.E., Myers, G.D., et al. (2018). Tisagenlecleucel in children and young adults with B-cell lymphoblastic leukemia. N. Engl. J. Med. *378*, 439–448.

van Mierlo, G.J., den Boer, A.T., Medema, J.P., van der Voort, E.I., Fransen, M.F., Offringa, R., Melief, C.J., and Toes, R.E. (2002). CD40 stimulation leads to effective therapy of CD40(-) tumors through induction of strong systemic cytotoxic T lymphocyte immunity. Proc. Natl. Acad. Sci. U S A *99*, 5561–5566.

Minard-Colin, V., Xiu, Y., Poe, J.C., Horikawa, M., Magro, C.M., Hamaguchi, Y., Haas, K.M., and Tedder, T.F. (2008). Lymphoma depletion during CD20 immunotherapy in mice is mediated by macrophage FcgammaRI, FcgammaRIII, and FcgammaRIV. Blood *112*, 1205–1213.

Moore, M.W., Carbone, F.R., and Bevan, M.J. (1988). Introduction of soluble protein into the class I pathway of antigen processing and presentation. Cell *54*, 777–785.

Neelapu, S.S., Locke, F.L., Bartlett, N.L., Lekakis, L.J., Miklos, D.B., Jacobson, C.A., Braunschweig, I., Oluwole, O.O., Siddiqi, T., Lin, Y., et al. (2017). Axicabtagene ciloleucel CAR T-cell therapy in refractory large B-cell lymphoma. N. Engl. J. Med. *377*, 2531–2544.

Ngiow, S.F., Young, A., Blake, S.J., Hill, G.R., Yagita, H., Teng, M.W., Korman, A.J., and Smyth, M.J. (2016). Agonistic CD40 mAb-driven IL12 reverses resistance to anti-PD1 in a T-cell-rich tumor. Cancer Res. *76*, 6266–6277.

Nimmerjahn, F., and Ravetch, J.V. (2006). Fcgamma receptors: old friends and new family members. Immunity *24*, 19–28.

Nimmerjahn, F., and Ravetch, J.V. (2008). Fcgamma receptors as regulators of immune responses. Nat. Rev. Immunol. *8*, 34–47.

Nimmerjahn, F., Lux, A., Albert, H., Woigk, M., Lehmann, C., Dudziak, D., Smith, P., and Ravetch, J.V. (2010). FcgammaRIV deletion reveals its central role for IgG2a and IgG2b activity in vivo. Proc. Natl. Acad. Sci. U S A *107*, 19396–19401.

O'Hara, M.H., O. Reilly, E.M., Rosemarie, M., Varadhachary, G., Wainberg, Z.A., Ko, A., Fisher, G.A., Rahma, O., Lyman, J.P., Cabanski, C.R., et al. (2019). A phase Ib study of CD40 agonistic monoclonal antibody APX005M together with gemcitabine (Gem) and nab-paclitaxel (NP) with or without nivolumab (Nivo) in untreated metastatic ductal pancreatic adenocarcinoma (PDAC) patients [abstract]. Cancer Res. *79* (13 Suppl), Abstract nr CT004.

Oura, T., Yamashita, K., Suzuki, T., Fukumori, D., Watanabe, M., Hirokata, G., Wakayama, K., Taniguchi, M., Shimamura, T., Miura, T., et al. (2012). Long-term hepatic allograft acceptance based on CD40 blockade by ASKP1240 in nonhuman primates. Am. J. Transplant. *12*, 1740–1754.

Pauls, S.D., and Marshall, A.J. (2017). Regulation of immune cell signaling by SHIP1: a phosphatase, scaffold protein, and potential therapeutic target. Eur. J. Immunol. *47*, 932–945.

Perry, C.J., Munoz-Rojas, A.R., Meeth, K.M., Kellman, L.N., Amezquita, R.A., Thakral, D., Du, V.Y., Wang, J.X., Damsky, W., Kuhlmann, A.L., et al. (2018). Myeloid-targeted immunotherapies act in synergy to induce inflammation and antitumor immunity. J. Exp. Med. *215*, 877–893.

Peters, A.L., Stunz, L.L., and Bishop, G.A. (2009). CD40 and autoimmunity: the dark side of a great activator. Semin. Immunol. *21*, 293–300.

Pinelli, D.F., and Ford, M.L. (2015). Novel insights into anti-CD40/CD154 immunotherapy in transplant tolerance. Immunotherapy *7*, 399–410.

Pooley, J.L., Heath, W.R., and Shortman, K. (2001). Cutting edge: intravenous soluble antigen is presented to CD4 T cells by CD8- dendritic cells, but cross-presented to CD8 T cells by CD8+ dendritic cells. J. Immunol. *166*, 5327–5330.

Richman, L.P., and Vonderheide, R.H. (2014). Role of crosslinking for agonistic CD40 monoclonal antibodies as immune therapy of cancer. Cancer Immunol. Res. *2*, 19–26.

Ridge, J.P., Di Rosa, F., and Matzinger, P. (1998). A conditioned dendritic cell can be a temporal bridge between a CD4+ T-helper and a T-killer cell. Nature *393*, 474–478.

Rigamonti, N., Schlegel, A., Barsin, S., Schwestermann, J., Mangold, S., Kaufmann, Y., Zitt, C., Veitonmäki, N., Levitsky, V., and Metz, C. (2019). Fibroblast activation protein (FAP)-selective delivery of CD40 agonistic DARPin®molecule for tumor-localized immune activation [abstract]. Cancer Res. *79* (13 Suppl), Abstract no. 3251.

Roghanian, A., Teige, I., Martensson, L., Cox, K.L., Kovacek, M., Ljungars, A., Mattson, J., Sundberg, A., Vaughan, A.T., Shah, V., et al. (2015). Antagonistic human FcgammaRIIB (CD32B) antibodies have anti-tumor activity and overcome resistance to antibody therapy in vivo. Cancer Cell *27*, 473–488.

Sallusto, F., and Lanzavecchia, A. (1994). Efficient presentation of soluble antigen by cultured human dendritic cells is maintained by granulocyte/macrophage colony-stimulating factor plus interleukin 4 and downregulated by tumor necrosis factor alpha. J. Exp. Med. *179*, 1109–1118.

Schoenberger, S.P., Toes, R.E., van der Voort, E.I., Offringa, R., and Melief, C.J. (1998). T-cell help for cytotoxic T lymphocytes is mediated by CD40-CD40L interactions. Nature *393*, 480–483.

Segal, N.H., Logan, T.F., Hodi, F.S., McDermott, D., Melero, I., Hamid, O., Schmidt, H., Robert, C., Chiarion-Sileni, V., Ascierto, P.A., et al. (2017). Results from an integrated safety analysis of urelumab, an agonist anti-CD137 monoclonal antibody. Clin. Cancer Res. *23*, 1929–1936.

Senhaji, N., Kojok, K., Darif, Y., Fadainia, C., and Zaid, Y. (2015). The contribution of CD40/CD40L axis in inflammatory bowel disease: an update. Front Immunol. *6*, 529.

Thompson, E.A., Liang, F., Lindgren, G., Sandgren, K.J., Quinn, K.M., Darrah, P.A., Koup, R.A., Seder, R.A., Kedl, R.M., and Lore, K. (2015). Human anti-CD40 antibody and poly IC:LC adjuvant combination induces potent T cell responses in the lung of nonhuman primates. J. Immunol. *195*, 1015–1024.

Todryk, S.M., Tutt, A.L., Green, M.H., Smallwood, J.A., Halanek, N., Dalgleish, A.G., and Glennie, M.J. (2001). CD40 ligation for immunotherapy of solid tumours. J. Immunol. Methods *248*, 139–147.

Tutt, A.L., O'Brien, L., Hussain, A., Crowther, G.R., French, R.R., and Glennie, M.J. (2002). T cell immunity to lymphoma following treatment with anti-CD40 monoclonal antibody. J. Immunol. *168*, 2720–2728.

Vafa, O., Gilliland, G.L., Brezski, R.J., Strake, B., Wilkinson, T., Lacy, E.R., Scallon, B., Teplyakov, A., Malia, T.J., and Strohl, W.R. (2014). An engineered Fc variant of an IgG eliminates all immune effector functions via structural perturbations. Methods *65*, 114–126.

Vaughan, A.T., Iriyama, C., Beers, S.A., Chan, C.H., Lim, S.H., Williams, E.L., Shah, V., Roghanian, A., Frendeus, B., Glennie, M.J., and Cragg, M.S. (2014). Inhibitory FcgammaRIIb (CD32b) becomes activated by therapeutic mAb in both *cis* and *trans* and drives internalization according to antibody specificity. Blood *123*, 669–677.

Vidarsson, G., Dekkers, G., and Rispens, T. (2014). IgG subclasses and allotypes: from structure to effector functions. Front Immunol. *5*, 520.

Visvanathan, S., Ramanujam, M., Schoelch, C., Baum, P., Vinisko, R., Thiedmann, R., Müller-Ladner, U., Daniluk, S., Ptaszyński, R., Padula, S., et al. (2016). Treatment with BI 655064 (antagonistic anti-CD40 antibody) modulates clinical and biomarker parameters associated with rheumatoid arthritis (RA) [abstract]. Arthritis Rheumatol. *68* (*Suppl. 10*), 1588.

Vitale, L.A., Thomas, L.J., He, L.Z., O'Neill, T., Widger, J., Crocker, A., Sundarapandiyan, K., Storey, J.R., Forsberg, E.M., Weidlick, J., et al. (2019).

Development of CDX-1140, an agonist CD40 antibody for cancer immunotherapy. Cancer Immunol. Immunother. *68*, 233–245.

Vonderheide, R.H. (2019). CD40 agonist antibodies in cancer immunotherapy. Annu. Rev. Med. *71*, 47–58.

Vonderheide, R.H., and Glennie, M.J. (2013). Agonistic CD40 antibodies and cancer therapy. Clin. Cancer Res. *19*, 1035–1043.

Vonderheide, R.H., Flaherty, K.T., Khalil, M., Stumacher, M.S., Bajor, D.L., Hutnick, N.A., Sullivan, P., Mahany, J.J., Gallagher, M., Kramer, A., et al. (2007). Clinical activity and immune modulation in cancer patients treated with CP-870,893, a novel CD40 agonist monoclonal antibody. J. Clin. Oncol. *25*, 876–883.

Ward-Kavanagh, L.K., Lin, W.W., Sedy, J.R., and Ware, C.F. (2016). The TNF receptor superfamily in co-stimulating and co-inhibitory responses. Immunity *44*, 1005–1019.

Watanabe, M., Yamashita, K., Suzuki, T., Kamachi, H., Kuraya, D., Koshizuka, Y., Ogura, M., Yoshida, T., Aoyagi, T., Fukumori, D., et al. (2013). ASKP1240, a fully human anti-CD40 monoclonal antibody, prolongs pancreatic islet allograft survival in nonhuman primates. Am. J. Transplant. *13*, 1976–1988.

Waterhouse, A., Bertoni, M., Bienert, S., Studer, G., Tauriello, G., Gumienny, R., Heer, F.T., de Beer, T.A.P., Rempfer, C., Bordoli, L., et al. (2018). SWISS-MODEL: homology modelling of protein structures and complexes. Nucleic Acids Res. *46*, W296–W303.

White, A.L., Chan, H.T., Roghanian, A., French, R.R., Mockridge, C.I., Tutt, A.L., Dixon, S.V., Ajona, D., Verbeek, J.S., Al-Shamkhani, A., et al. (2011). Interaction with FcgammaRIIB is critical for the agonistic activity of anti-CD40 monoclonal antibody. J. Immunol. *187*, 1754–1763.

White, A.L., Chan, H.T., French, R.R., Willoughby, J., Mockridge, C.I., Roghanian, A., Penfold, C.A., Booth, S.G., Dodhy, A., Polak, M.E., et al. (2015). Conformation of the human immunoglobulin G2 hinge imparts superagonistic properties to immunostimulatory anticancer antibodies. Cancer Cell *27*, 138–148.

Wiehagen, K.R., Girgis, N.M., Yamada, D.H., Smith, A.A., Chan, S.R., Grewal, I.S., Quigley, M., and Verona, R.I. (2017). Combination of CD40 agonism and CSF-1R blockade reconditions tumor-associated macrophages and drives potent antitumor immunity. Cancer Immunol. Res. *5*, 1109–1121.

Wypych, J., Li, M., Guo, A., Zhang, Z., Martinez, T., Allen, M.J., Fodor, S., Kelner, D.N., Flynn, G.C., Liu, Y.D., et al. (2008). Human IgG2 antibodies display disulfide-mediated structural isoforms. J. Biol. Chem. *283*, 16194–16205.

Ye, S., Cohen, D., Belmar, N.A., Choi, D., Tan, S.S., Sho, M., Akamatsu, Y., Kim, H., Iyer, R., Cabel, J.M., et al. (2019). A bispecific molecule targeting CD40 and tumor antigen mesothelin enhances tumor-specific immunity. Cancer Immunol. Res. *7* (11), 1864–1875.

Yu, X., Chan, H.T.C., Orr, C.M., Dadas, O., Booth, S.G., Dahal, L.N., Penfold, C.A., O'Brien, L., Mockridge, C.I., French, R.R., et al. (2018). Complex interplay between epitope specificity and isotype dictates the biological activity of anti-human CD40 antibodies. Cancer Cell *33*, 664–675.e4.

van Zundert, G.C.P., Rodrigues, J., Trellet, M., Schmitz, C., Kastritis, P.L., Karaca, E., Melquiond, A.S.J., van Dijk, M., de Vries, S.J., and Bonvin, A. (2016). The HADDOCK2.2 web server: user-friendly integrative modeling of biomolecular complexes. J. Mol. Biol. *428*, 720–725.

STAR★METHODS

KEY RESOURCES TABLE

REAGENT or RESOURCE	SOURCE	IDENTIFIER
Antibodies		
Goat F(ab')2 Anti-Human IgG - Fc (PE)	Abcam	Cat# ab98596; RRID:AB_10673825
341G2 and isotype variants	In house	Published patent: US8716451B2
CP870,893 and isotype variants	In house	Published patent: US20090130715A1
ADC1013 and isotype variants	In house	Published patent: WO2016023960A1
APX005M and isotype variants	In house	Published patent: WO2014070934A1
SGN40 and isotype variants	In house	Published patent: WO2007075326A2
HCD122 and isotype variants	In house	Published patent: WO2012075111A1
Abbv-323 and isotype variants	In house	Published patent: WO2016196314A1
Goat Anti-Human IgG Fc (HRP)	Abcam	Cat# ab98624; RRID:AB_10673832
Goat F(ab')2 Anti-Mouse IgG Fc (PE)	Abcam	Cat# ab98649; RRID:AB_10674947
Mouse monoclonal anti-OVA IgG (clone KB4)	In house	N/A
Anti-mouse CD19-APC	Biolegend	Cat# 152410; Clone 1D3; RRID:AB_2629839
Anti-mouse CD45.2-FITC	Biolegend	Cat# 109806; clone 104; RRID:AB_313443
Anti-mouse CD8-APC	eBioscience	Cat# 17-0081-82; Clone 53-6.7; RRID:AB_469335
Anti-mouse CD23-PE	eBioscience	Cat# 12-0232-83; Clone B3B4; RRID:AB_465594
Anti-mouse CD11c-Pacific Blue	Biolegend	Cat# 117322; Clone N418; RRID:AB_755988
Anti-mouse DEC205-PE-Cy7	Biolegend	Cat# 138210; Clone NLDC-145; RRID:AB_10643581
Anti-mouse CD86-APC	Biolegend	Cat# 105012; Clone GL-1; RRID:AB_493342
Anti-mouse CD80-APC/Fire™ 750	Biolegend	Cat# 104740; Clone 16-10A1; RRID:AB_2687095
Anti-human CD86-Alexa Fluor® 488	Biolegend	Cat# 305414; Clone IT2.2; RRID:AB_528881
Anti-human CD70-PE	Biolegend	Cat# 355104; Clone 113-16; AB_2561431
Anti-human CD80-PerCP/Cy5.5	Biolegend	Cat# 305232; Clone 2D10; RRID:AB_2566491
Anti-mouse PD-L1 (B7-H1)	BioXCell	Cat# BE0101; Clone 10F.9G2; RRID:AB_10949073
Anti-mouse CD27 (clone AT124-1, mouse IgG1)	In house	N/A
Anti-mouse CD8-Brilliant Violet 510™	Biolegend	Cat# 100752; Clone 53-6.7; RRID:AB_2563057
Anti-mouse FcγR2B-FITC (clone AT150-2)	In house	N/A
Biological Samples		
Healthy human PBMC	Southampton General Hospital National Blood Service	N/A
Chemicals, Peptides, and Recombinant Proteins		
PE-labeled SIINFEKL/H-2K^b tetramer	In house	N/A
Ovalbumin	Sigma-Aldrich	Cat# A2512
Lymphoprep	Axis-Shield	Cat# 07861
Erytholyse Red Cell Lysis Buffer	AbD Serotec	Cat# BUF04B
N-Glycosidase F (PNGaseF)	Promega	Cat#V483A
DAPI	Life Technologies	Cat# D1306
Recombinant human CD40 and domain truncated variants	In house	N/A
Recombinant human CD40 alanine-scanning mutants	In house	N/A
Tritium thymidine (Methyl-3^H)	PerkinElmer	Cat# NET027250UC
PE-labeled RAHYNIVTF/H-2D^b tetramer	In house	N/A
HPV-16 peptide GQAEPDRAHYNI VTFCCKCDSTLRLCVQSTHVDIR	GL Biochem	Customized

(*Continued on next page*)

Continued

REAGENT or RESOURCE	SOURCE	IDENTIFIER
Recombinant human CD40 ligand	In house	N/A
Critical Commercial Assays		
ELISA assay to detect mouse TNF-α	Biolegend	Cat# 430901
ELISA assay to detect mouse IL-6	Biolegend	Cat# 431301
ELISA assay to detect mouse IFN-γ	Thermofisher	Cat# BMS606TWO
Luminex bioplex to detect human TNF-α, IP-10, MCP-1, IL-12p70 and IFN-γ	Biorad	Customized
Mouse B cell isolation set	Stemcell Technologies	Cat# 19854
Human monocyte isolation set	Miltenyi Biotech	Cat# 130-096-537
Human CD4+ T cell isolation set	Stemcell Technologies	Cat# 19852
Deposited Data		
SAXS data for CD40 - 341G2 F(ab) complex	Small angle scattering biological data bank	Accession Code: SASDHN8
Experimental Models: Cell Lines		
Mouse tumor: EG7	(White et al., 2015)	N/A
Mouse tumor: TC1	(Lin et al., 1996)	N/A
Mouse tumor: MC38	Dr Rienk Offringa (German Cancer Research Center)	N/A
Jurkat	ATCC	TIB-152
Ramos	ATCC	CRL-1596
CHO-k1	ATCC	CCL-61
ExpiCHO	Thermofisher	A29127
FreeStyle 293F	Thermofisher	R79007
Experimental Models: Organisms/Strains		
OT-I TCR transgenic on C57BL/6 background	Charles River Laboratories	Strain code: 642
hCD40Tg on C57BL/6 background	(White et al., 2015)	N/A
hCD40Tg/*Fcer1g*$^{-/-}$ /*FcγR2b*$^{-/-}$ on C57BL/6 background	This paper	N/A
hCD40Tg/m*FcγR2b*$^{-/-}$/h*FcγR2b*$^{+/-}$ on C57BL/6 background	(Roghanian et al., 2015)	N/A
Software and Algorithms		
Prism	GraphPad	N/A
Flowjo	BD Biosciences	N/A
FCS Express	De Novo Software	N/A
Leica Application Suite X	Leica	N/A
ScAtter v3.2h - R. Rambo	Dr. Robert Rambo (Diamond Light Source)	http://www.bioisis.net/

RESOURCE AVAILABILITY

Lead Contact

Further information and requests for resources and reagents should be directed to and will be fulfilled by the Lead Contact, Professor Mark Cragg (msc@soton.ac.uk)

Materials Availability

All unique/stable reagents generated in this study are available from the Lead Contact with a completed Materials Transfer Agreement.

Data and Code Availability

The original SAXS data for CD40 - 341G2 F(ab) complex have been deposited into the small angle scattering biological data bank. SASBDB: SASDHN8

Data supporting the current study are available from the corresponding author upon request.

CellPress
OPEN ACCESS

EXPERIMENTAL MODEL AND SUBJECT DETAILS

Mice

hCD40 transgenic mice (hCD40Tg) were kindly provided by Randolph Noelle (King's College, London) and were described before (Ahonen et al., 2002). *Fcer1g*$^{-/-}$, *Fcgr2b*$^{-/-}$ mice (C57BL/6 background) were generated by Dr J. Sjef Verbeek (Toin University of Yokohama, Japan), and FcγR null mice (*Fcer1g*$^{-/-}$ x *Fcgr2b*$^{-/-}$) were generated through breeding *Fcer1g*$^{-/-}$ and *Fcgr2b*$^{-/-}$ mice to generate homozygous FcγR null mice (Fransen et al., 2018). The hCD40Tg/*Fcgr2b*$^{-/-}$ mice were generated by crossing hCD40Tg mice with *Fcgr2b*$^{-/-}$ mice, and hCD40Tg/FcγR null mice were generated by crossing hCD40Tg mice with homozygous FcγR null mice. The hCD40Tg/m*Fcgr2b*$^{-/-}$/h*Fcgr2b*$^{+/-}$ mice were generated by crossing hCD40Tg mice with m*Fcgr2b*$^{-/-}$/h*Fcgr2b*$^{+/-}$ mice described previously (Roghanian et al., 2015). The presence of hCD40 and lack of FcγRs were confirmed by flow cytometry. The lack of FcγRIIB, the only FcγR known to be expressed by murine B cells, is shown in Figure S7A. OTI TCR transgenic mice were from Charles River Laboratories (Kent, UK). All animals, including wild-type C57BL/6 mice, were maintained and bred in-house. For all experiments, age and sex-matched animals were randomized and assigned to experimental groups. All experiments were conducted under UK Home Office licence numbers PB24EEE31, P4D9C89EA, P540CBA98, and P39FE2AA7 and following approval by local ethical committees, reporting to the Home Office Animal Welfare Ethical Review Board (AWERB) at the University of Southampton.

Human Samples

Human B cells, T cells and monocytes were purified from human PBMCs obtained from healthy donor leukocyte cones through Southampton National Blood Services with prior informed consent and Ethical approval from the East of Scotland Research Ethics Service, Tayside, UK.

Cell Lines

MC38 and EG7 (Moore et al., 1988) (ATCC) cell lines were maintained in a humidified incubator at 37°C and 5% CO_2 and cultured in RPMI supplemented with 10% heat inactivated FBS, 2 mM L-glutamine, 1 mM pyruvate, 100 U/mL penicillin and 100 μg/mL streptomycin. The MC38 cell line was kindly provided by Dr Rienk Offringa (German Cancer Research Center). EG7 cells were cultured in the additional presence of 0.4mg/mL geneticin and 50 μM β-Mercaptoethanol. The TC1 cell line (Lin et al., 1996) transduced with the HPV E6 and E7 oncogenes was originally from Dr Bjarne Bogen (University of Oslo), and cultured in IMDM supplemented with 10% heat inactivated FBS, 2mM L-glutamine, 1mM pyruvate, 100 U/mL penicillin, 100 μg/mL streptomycin, 0.4 mg/mL geneticin and 50 μM β-Mercaptoethanol.

METHOD DETAILS

Antibodies and Reagents

ChiLob 7/4 and Lob 7/6 were generated in-house, their variable domain sequences were amplified by PCR from the hybridoma cells to allow isotype switching. The variable domain sequences of 341G2 (US8716451B2), CP870,893 (US20090130715A1), ADC1013 (WO2016023960A1), APX005M (WO2014070934A1), SGN40 (WO2007075326A2), HCD122 (WO2012075111A1) and Abbv-323 (WO2016196314A1) were derived from published patents and genes containing these sequences were synthesized by GeneArt. DNA fragments encoding the light and heavy chain variable domain sequences were then cloned into the pEE12.4 and pEE6.4 expression vectors (Lonza, UK), respectively at the HindIII (N-terminus end) and EcoRI (C-terminus end) restriction sites. pEE6.4 vectors encoding different IgG isotypes were generated to allow isotype switching. Fc mutagenesis was performed using the QuickChange Site-Directed Mutagenesis Kit (Agilent, UK). Antibodies were produced either from hybridoma cells or by transient expression in CHO cells and subsequently purified on protein A columns (GE healthcare, UK). All antibodies were checked to contain < 1% aggregate determined by HPLC and tested to contain < 5EU endotoxin per 1 mg antibody assessed by the Endosafe-PTS portable test system (Charles River Laboratories, L'Arbresle, France). Antibody deglycosylation was achieved by treatment with PNGase F (Promega, UK) for 48 hours at 37°C, and deglycosylation was confirmed by a heavy chain band shift in reducing SDS-PAGE. Capillary electrophoresis (CE-SDS) was used to distinguish the A and B forms of hIgG2 using a Beckman PA800 Plus analyser. The CE-SDS profiles of 341G2 and CFZ533 are shown in Figure S5.

SIINFEKL peptide was from Peptide Protein Research Ltd. The HPV-16 peptides RAHYNIVTF ($E7_{49-57}$, UniProt numbering) and GQAEPDRAHYNIVTFCCKCDSTLRLCVQSTHVDIR ($E7_{43-77}$, UniProt numbering) were purchased from GL Biochem (Shanghai, China). PE-conjugated SIINFEKL/H-2Kb tetramer and PE-conjugated RAHYNIVTF/H-2Db tetramer were both produced in-house. Chicken ovalbumin was purchased from Sigma. Recombinant trimeric CD40L was produced in house. DNA construct encoding human CD40L (Met113-Leu261) fused with a FLAG tag and GCN4 leucine zipper motif via a (G3S)3 linker at the N-terminus was synthesized by GeneArt and then subcloned into pDSG104 vector (IBA Life Sciences, Germany). The plasmid was transfected into MEXI293E cells (IBA Lifesciences) for 7 days before purification by anti-FLAG Affinity Gel (Sigma, UK).

Flow cytometry experiments were conducted using FACSCalibur or FACSCanto II (both from BD Biosciences)

Generation of CD40 Constructs and Epitope Mapping

DNA sequences encoding the wild-type full-length extracellular (EC) domain of CD40 (i.e. CRD1-4), or truncated variants CRD2-4, CRD3-4 and CRD4 were amplified by PCR and cloned into pcDNA3.1 for recombinant soluble protein expression. For cell surface expression, the same constructs were cloned into the pCIpuro vector (Promega) that contains the CD40 transmembrane domain at the N-terminus for membrane anchoring. The full-length human CD40EC-GFP fusion construct was cloned into pCIpuro for cell surface expression. For recombinant soluble protein expression of truncated CD40 variants, FreeStyle 293F cells (Thermo Fisher Scientific) were transfected with the DNA/PEI complex and cultured for 7 days before supernatant was harvested for purification using Ni-NTA column (GE Healthcare). For cell surface expression, CHOk1 cells were transfected with various CD40 constructs using GenePORTER Transfection reagent (Amsbio, UK) and stable clones were selected using puromycin (Invivogen, UK).

High-resolution epitope mapping of 341G2 and HCD122 was achieved by alanine scanning mutagenesis of CD40 CRD1 and CRD2 domains as described previously (Yu et al., 2018). Briefly, two consecutive residues were mutated to alanines throughout CRD1 and CRD2; each construct was expressed on the CHO-k1 cell surface and stable clones were selected using 10 μg/mL puromycin (Invivogen, USA). For recombinant soluble protein expression of CD40 alanine scanning mutants, DNA was cloned into the pCIpuro vector with a C-terminal His-tag, expressed in ExpiCHO cells and purified using a Ni-NTA column.

Confocal Microscopy

Jurkat cells were transfected with pCIpuro plasmid encoding a human CD40EC-GFP construct using Nucleofector Kit V (Lonza) and stable clones were selected using 1 μg/mL puromycin. To assess the effect of anti-CD40 mAb on CD40 receptor clustering, cells were incubated with 10 μg/mL anti-CD40 mAb for one hour at 37°C and then fixed with methanol before DAPI staining for the nucleus. Confocal images were acquired using a Leica SP8 confocal microscope and analysed using Leica Application Suite X (both from Leica).

In Vitro Receptor Internalization Assay

The level of CD40 internalization was quantified using a fluorescence quenching assay as previously described (Austin et al., 2004). Briefly, anti-CD40 mAb were labelled with AF488 using AF488 Antibody Labeling Kit (Thermo Fisher Scientific) and then added to various purified B cell cultures as indicated for 10, 30, 60, 120 or 180 minutes at 4°C or 37°C. Cells were then washed and half the cells were treated with anti-AF488 antibody (Thermo Fisher Scientific) at 4°C that quenches AF488 fluorescence. The remaining AF488 fluorescence analysed by flow cytometry correlates to internalized CD40. % Total Expression quantifies remaining cell surface-bound CD40 and was calculated as % (unquenched fluorescence – quenched fluorescence)/(unquenched fluorescence).

Surface Plasmon Resonance

The affinity of various anti-CD40 mAbs for hCD40 was analysed using a Biacore T100, all reagents used were from GE Healthcare Life Sciences, UK. hCD40 was immobilized onto a CM5 sensor chip via amine coupling according to manufacturer's recommendations. Anti-CD40 mAbs were injected through the flow cells at 250, 50, 10, 2, 0.4, and 0 nM in HBS-EP+ running buffer at a flow rate of 30 μL/min, allowing 300 seconds for association and 300 seconds for dissociation. The sensorgrams were fitted with 1:1 Binding model, and the ka, kd, and KD were calculated using Biacore Bioevaluation software. The parental 341G2 and its Fc variants bound CD40 with similar affinities (Figure S7B, Table S1). To analyse the affinity of anti-CD40 for hCD40 mutants, His-tagged soluble hCD40 variants were captured for 30 seconds using anti-His mAb immobilized on CM5 chip, and Lob 7/6 and 341G2 (both hIgG1 isotype) were injected at 1000, 333, 111, 37, 12.4, and 4.1 nM using the Biacore T100 instrument. The association and dissociation phases lasted 180 and 300 seconds, respectively.

Western Blot

His-tagged recombinant CD40 proteins were run on 18% polyacrylamide gels and transferred onto nitrocellulose membranes. The membranes were blocked in PBS 5% non-fat dried milk 0.05% Tween 20 and then incubated with various anti-CD40 mAb at 4°C overnight before detection using secondary polyclonal goat anti-human IgG-HRP (Abcam, UK). The membranes were washed with PBS before the addition of ECL and the signals were captured on a UVP Biospectrum Imaging System.

Size Exclusion Chromatography Coupled with Small-Angle X-Ray Scattering (SEC-SAXS)

A F(ab) fragment of 341G2 hIgG1 was generated by digestion with papain-agarose (Thermo Fisher Scientific) followed by incubation with protein A to deplete intact IgG1 and Fc fragments, before further purification by SEC. Purified 341G2 F(ab) and hCD40 EC domain were mixed at a 1:1 molar ratio for 1 hour and the F(ab)-CD40 EC complex was subsequently purified by SEC. SEC-SAXS data were collected at the B21 beamline at Diamond Light Source, UK. 45 μL of F(ab)-CD40 EC complex at 7.7 mg/mL was injected in a Superdex 200 increase 3.2/200 column using PBS as the SEC buffer at a flow rate of 0.075 mL/minute. Measurements were performed at 20°C. Data were recorded from 620 three second continuous exposures using a Pilatus 2M detector. Data were processed and analysed using the Scatter software (V3.2h, R.Rambo, DLS). Frames for analysis were selected using the subtract tab, allowing for identification of buffer frames from the SEC-buffer flow through and scattering frames from the elution peak. The final curve was generated after buffer subtraction. A list of SAXS-derived parameters are shown below:

Sample Details	CD40EC-341G2 Fab
Organism	*Homo sapiens*
Uniprot sequence ID (residues in construct)	–
Extinction coefficient	101760
Protein mass from chemical composition	67637
SEC-SAXS, Superdex 200 increase 3.2/200	
Loading concentration (mg/ml)	7.7
Injection volume	45 μL
Flow rate (mL/min^{-1})	0.0075
SEC-buffer	PBS

SAXS Data-Collection Parameters	
Instrument/data-processing	B21, Diamond Light Source (DLS), Harwell (UK)
Wavelength (Å)	1.0
Beamsize (μm)	1 x 1
Camera length (m)	4.0
q measurement range (Å^{-1})	0.004 – 0.408
Absolute scaling method	Comparison with BSA
Normalisation	To integrated intensity from beam-stop diode
Monitoring for radiation damage	Frame comparison
Exposure time	1860 (620 x 3 s)
Sample temperature (°C)	20

Software Employed for SAXS Data Reduction, Analysis and Interpretation	
SAXS data reduction	*DAWN* pipeline (DLS, Harwell, UK)
Extinction coefficient estimate	*ProtParam*
Basic analyses: Guinier, P(r), Vp	PRIMUSqt from ATSAS 2.8.2
Atomic structure modelling	CRYSOL from PRIMUSqt in ATSAS 2.8.2
Sequence modelling	SWISS-MODEL, Phyre2
Molecular graphics	PyMol 2.3.0

Homology Modelling and Docking

341G2- the sequence of 341G2 Fab was submitted to the SWISS-MODEL server to generate a homology model (Waterhouse et al., 2018). The model was generated based on the 56.a.09 antibody (PDB: 5K9J) with which 341G2 Fab shares 90.4% sequence identity and 0.57 sequence similarity score. The Global model quality estimation (GMQE) score for the resulting model was 0.97 with the QMEAN score being 0.62 indicating good confidence in the quality of the generated model.

CD40- Phyre2 (Kelley et al., 2015) was used to generate a model of CD40EC. The CD40EC sequence (Uniprot: P25942) was submitted for modelling, returning a model based on PDB: 5DMJ that had a confidence score of 100% with 97% coverage indicating a suitable model for further analysis.

Docking of 341G2 F(ab) and CD40 was performed using the HADDOCK 2.2 webserver (van Zundert et al., 2016). Residues on CD40 known to affect binding (identified through alanine scanning mutagenesis experiments), and the CDR loop residues of 341G2 were provided to the docking server as restraints in the docking following the HV-Epi9 protocol detailed in (Ambrosetti et al., 2020). For CD40 these residues were S49, D50, F67, L68, T75, H76, H78 and Q79. For the 341G2 model, the CDR loop residues were 26 to 34, 53 to 59, 104 to 112, 260 to 268, 284 to 288 and 325 to 331. The CDR loops were defined as active residues, while the CD40 residues were defined as active with a 9Å radius of passive residues. All HADDOCK settings were default apart from rigid-body refinement sampling which was increased to 5000 models and the flexible and water refinement sampling were increased to 400 models. Docking produced 10 clusters comprising 40 representative structures. Based on the knowledge that 341G2 and ChiLob 7/4 do not cross-block, a large number of the representative models were able to be excluded due to clashing with the Chilob 7/4 Fab from the crystal structure PDB: 6FAX when aligned on CD40 (Yu et al., 2018). This left a total of 11 models from 3 clusters that were taken

forward for further analysis. The remaining structures were validated against the SAXS data using the WAXSiS server to compare the structures to the experimental SAXS data. The models that best fitted the SAXS data were selected by their $\chi 2$ score. The best fitting model gave a $\chi 2$ score of 2.21 +/- 0.37, with a clear trend seen between better and worse fitting models based on relative binding orientation to CD40. This model also came from the cluster with the best HADDOCK score (-102.0 +/- 10.9). The flowchart for SEC-SAXS and subsequent homology modelling and docking is shown in Figure S8.

NFκB Assay

The Jurkat NF-κB GFP reporter cell line was purchased from System Biosciences, USA and then transfected with the pCIpuro vector encoding the full-length hCD40 using Lipofectamine2000 (Thermo Fisher Scientific). Stable clones were selected using 1 μg/mL puromycin. To study NFκB activation, cells were incubated with various anti-CD40 mAb for 8 hours at 37°C and the level of NFκB activation was measured by GFP production by flow cytometry.

B Cell Activation and Proliferation

The hCD40Tg B cells were purified from hCD40Tg mouse splenocytes, and human B cells were purified from healthy donor PBMC, both by magnetic negative selection kits (StemCell Technologies, UK). For B cell proliferation, $1x10^5$ B cells per well were incubated with different treatments in 96-well round bottom plates in a total of 200 uL media for various periods of time as indicated for individual experiments. Proliferation was assessed by adding 1 μCi of ^{3}H thymidine (PerkinElmer) to each well for the last 18 hours of incubation before cells were harvested and analysed for ^{3}H thymidine incorporation by TopCount. To assess B cell activation, cells were imaged 48 hours after the initial treatment using conventional light microscope (Olympus CKX41), and then stained for surface expression of CD80, CD86 and CD70 using flow cytometry. To assess the ability of 341G2 hIgG1 and 341G2 hIgG4 to block CD40L-mediated B cell activation, B cells were co-incubated with 2 μg/mL CD40L and 5 μg/mL anti-CD40 mAb.

Human Dendritic Cell Activation and Mixed Leukocyte Reaction

Human immature DCs were derived from $CD14^+$ monocytes as described before (Sallusto and Lanzavecchia, 1994). Briefly, $CD14^+$ monocytes were isolated from human PBMC by magnetic negative selection kit (Miltenyi Biotech, UK) and then cultured in the presence of 500 IU/mL IL-4 and 1000 IU/mL GM-CSF (both recombinant, produced in-house) for 6 days before phenotyping for the expression of CD11c and DC-SIGN by flow cytometry. To study the direct effect of anti-CD40 mAb, DCs were treated with 5 μg/mL anti-CD40 mAb for 48 hours and then the surface expression of CD86, CD80 and CD70 were analysed by flow cytometry. Moreover, the levels of TNF-α, IP-10, MCP-1, IL-12p70 and IFN-γ in the cell culture supernatant were quantified by Luminex Bioplex 200 instrument (Biorad, UK). For mixed leukocyte reaction, DCs were pre-treated with various anti-CD40 mAb for 24 hours before being washed and subsequently incubated with purified allogeneic $CD4^+$ T cells (Stemcell Technologies) at various ratios for 5 days. ^{3}H thymidine was added at 1 μCi per well for the last 18 hours to assess T cell proliferation.

In Vivo Assessment of Agonistic and Antagonistic Activity

To assess the antagonistic activity of anti-CD40 mAb *in vivo*, hCD40Tg mice were administered 500 μg OVA and 100 μg anti-CD40 mAb intravenously on day 0, and 100 μg anti-CD40 mAb intraperitoneally on day 3. Mice were bled on day 2 to quantify $CD19^+$ B cells in peripheral blood by flow cytometry and on day 18 to quantify the level anti-OVA IgG in serum. To detect anti-OVA IgG in serum, ELISA plates (Thermo Fisher Scientific) were coated with 5 μg/mL OVA in PBS overnight. The next day, plates were blocked with 1% BSA and then serially diluted serum was added to each well, the mouse anti-OVA IgG mAb KB4 (in-house) was used to create a standard curve. Bound anti-OVA IgG was detected by secondary goat anti-mouse IgG-HPR (Abcam). To assess the agonistic activity of anti-CD40 mAb, $1x10^5$ OTI cells were transferred into hCD40Tg mice via tail vein injection, the next day, 100 μg OVA and 30 μg anti-CD40 mAb were injected intravenously. The level of OTI expansion in peripheral blood was assessed 5 days after mAb treatment by staining for CD8 and SIINFEKL tetramer positive cells by flow cytometry.

To assess the effect of agonistic anti-CD40 mAb on B cells and DCs *in vivo*, hCD40Tg mice were administered 30 μg anti-CD40 mAb intravenously on day 0 and then bled and spleens harvested on day 2 to quantify $CD19^+$ B cells and CD23 expression by flow cytometry. Splenic DCs were gated as $CD11c^+CD8^+DEC205^+$ and analysed for the level of CD80 and CD86 by flow cytometry.

Serum Cytokine Analysis

hCD40Tg mice were administered 30 μg anti-CD40 mAb intravenously and bled 6 hours and 48 hours later to collect serum. Levels of IL-6, TNF-α and IFN-γ were quantified using ELISA MAX Standard Set kits according to manufacturer's protocols (Biolegend).

Tumor Models and Immunotherapy Treatment

All mice were monitored for tumor growth by digital caliper measurements three times a week. Mice were culled when the sum of the tumor length and width reached 30 mm or when the general health of animals fell below the criteria set out in the corresponding Home Office project license. The agonistic anti-CD40 mAb 341G2 hIgG2 was injected at 30 μg per dose. Tumor volume was calculated using the formula: $V = (W^2 \times L)/2$ where W is tumor width and L is tumor Length. All tumor cells were resuspended in PBS and 100 uL was injected on the right flank of mice.

MC38 colon carcinoma model- $5x10^5$ cells were injected subcutaneously and treatment started on day 6 when the sum of tumor length and width reached approximately 10mm. 30 μg anti-CD40 mAb alone or in combination with 100 μg anti-PD-L1 (clone 10F.9G2, BioXcell) or 100 μg anti-CD27 (AT124-1 mouse IgG1, in house) were injected intraperitoneally every 3 days for 2 doses.

EG7 thymoma model- $5x10^5$ cells were injected subcutaneously. On day 7, when the sum of tumor length and width reached ~ 10mm, $1x10^5$ OTI cells resuspended in PBS were adoptively transferred via tail vein injection; the following day, 30 μg anti-CD40 mAb was injected intravenously.

TC1 lung carcinoma model- $1x10^5$ TC1 cells were injected subcutaneously. On day 5, mice were vaccinated with either 150 μg or 3 μg long peptide (GQAEPDRAHYNIVTFCCKCDSTLRLCVQSTHVDIR) in combination with 30 μg anti-CD40 mAb intravenously. For monotherapy, mice were treated with 30 μg anti-CD40 mAb starting on day 5 every 3 days for 3 doses.

QUANTIFICATION AND STATISTICAL ANALYSIS

Flow cytometry data analysis was performed using either FCS Express software Version 3 (De Novo Software) or Flowjo Version 10.6 (BD Biosciences). All other data analysis were performed using GraphPad Prism 7.05 (GraphPad Software). Two-tailed, non-paired Student t test was used for most pairwise comparisons. For assays that investigate the effect of anti-CD40 mAb on human DCs, pairwise comparisons were made using two-tailed, paired Student t test. Statistical comparisons of survival curves were performed by Log-rank test. Throughout $*p < 0.05$, $**p < 0.01$, $***p < 0.001$.

Cancer Cell, Volume *37*

Supplemental Information

Isotype Switching Converts Anti-CD40 Antagonism to Agonism to Elicit Potent Antitumor Activity

Xiaojie Yu, H.T. Claude Chan, Hayden Fisher, Christine A. Penfold, Jinny Kim, Tatyana Inzhelevskaya, C. Ian Mockridge, Ruth R. French, Patrick J. Duriez, Leon R. Douglas, Vikki English, J. Sjef Verbeek, Ann L. White, Ivo Tews, Martin J. Glennie, and Mark S. Cragg

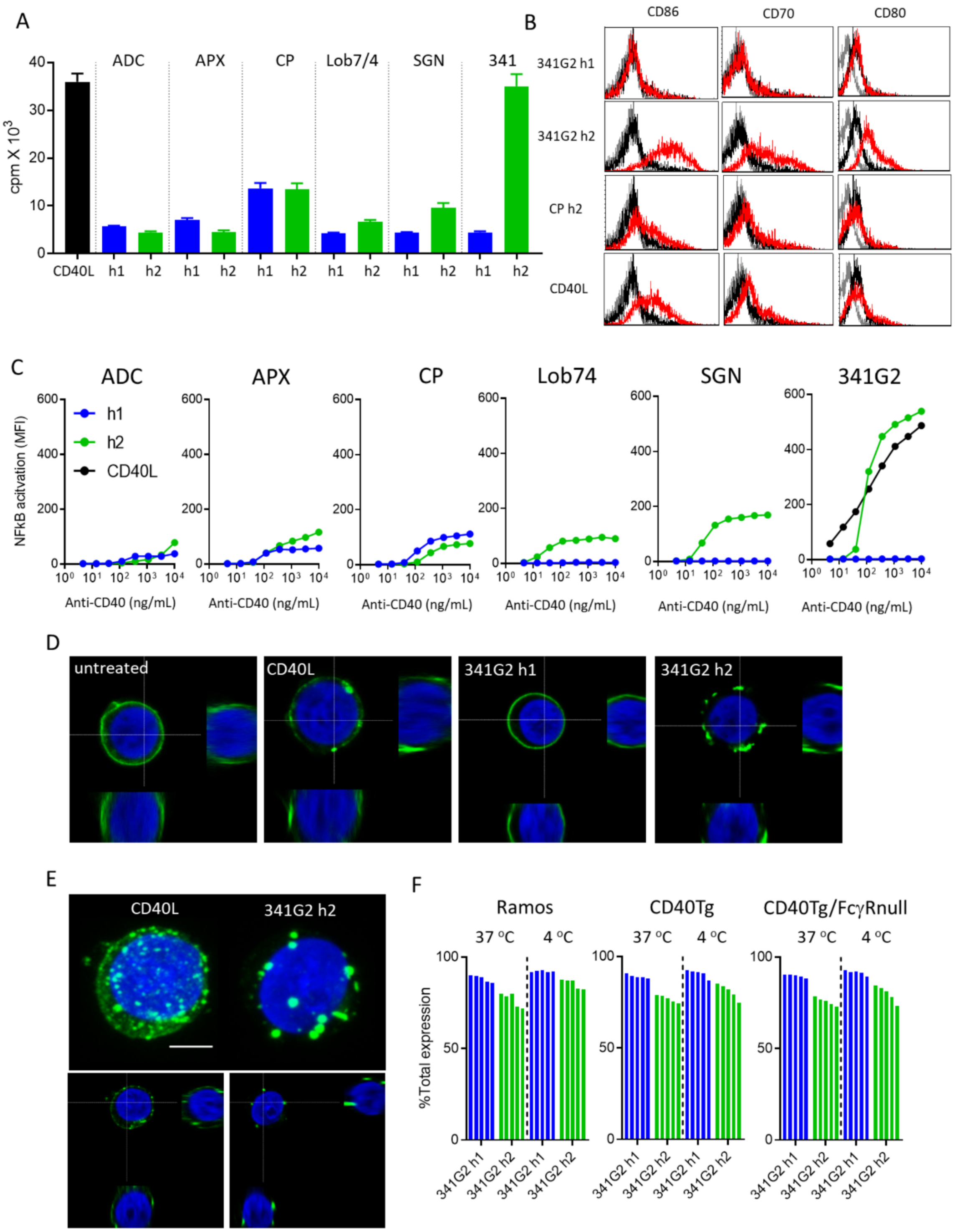

A
cpm X 10^3
ADC
APX
CP
Lob7/4
SGN
341
CD40L
h1
h2
B
CD86
CD70
CD80
341G2 h1
341G2 h2
CP h2
CD40L
C
ADC
APX
CP
Lob74
SGN
341G2
NFkB activation (MFI)
h1
h2
CD40L
Anti-CD40 (ng/mL)
D
untreated
CD40L
341G2 h1
341G2 h2
E
CD40L
341G2 h2
F
Ramos
CD40Tg
CD40Tg/FcγRnull
37 °C
4 °C
%Total expression
341G2 h1
341G2 h2

Figure S1, Related to Figure 3. Comparison of clinically-relevant anti-CD40 mAb in an NFκB activation assay

(A) Purified human B cells were incubated with 2 µg/mL of various clinical anti-CD40 mAb as indicated for 4 days. Proliferation was measured by ^{3}H thymidine incorporation. Means ± SEM, n = 4, data representative of three donors.

(B) Purified human B cells were incubated with 2 µg/mL of various clinical anti-CD40 mAb as indicated for 2 days and then analysed for surface expression of CD80, CD86 and CD70 by flow cytometry. Data representative of three donors.

(C) Jurkat NFκB GFP reporter cells stably transfected with hCD40 were incubated with various concentrations of different clinically-relevant anti-CD40 mAb for 8 hours and the level of NFκB activation was assessed by GFP expression using flow cytometry. Means ± SEM, n = 3, data representative of two experiments.

(D) Jurkat cells stably transfected with hCD40EC-GFP were treated with 10 µg/mL anti-CD40 mAb as indicated for 1 hour or (E) 24 hours at 37°C. Cells were then fixed, nuclear-stained using DAPI and imaged using a Leica SP8 confocal microscope. Z-stack and orthogonal images shown. Blue: nucleus; Green: hCD40EC-GFP. Scale bar: 4 µm. Image representative of at least ten images taken.

(F) Ramos cells and purified splenic B cells from hCD40Tg or hCD40Tg/FcγRnull mice were treated with AF488-labelled anti-CD40 mAb for 10, 30, 60, 120 or 180 minutes (left to right) at 4°C or 37°C as indicated. Cells were then washed and half the cells were treated with anti-AF488 mAb at 4°C to quench cell surface-associated AF488 fluorescence. Remaining cell surface-bound CD40 was expressed as % Total expression. Data representative of 2-3 experiments.

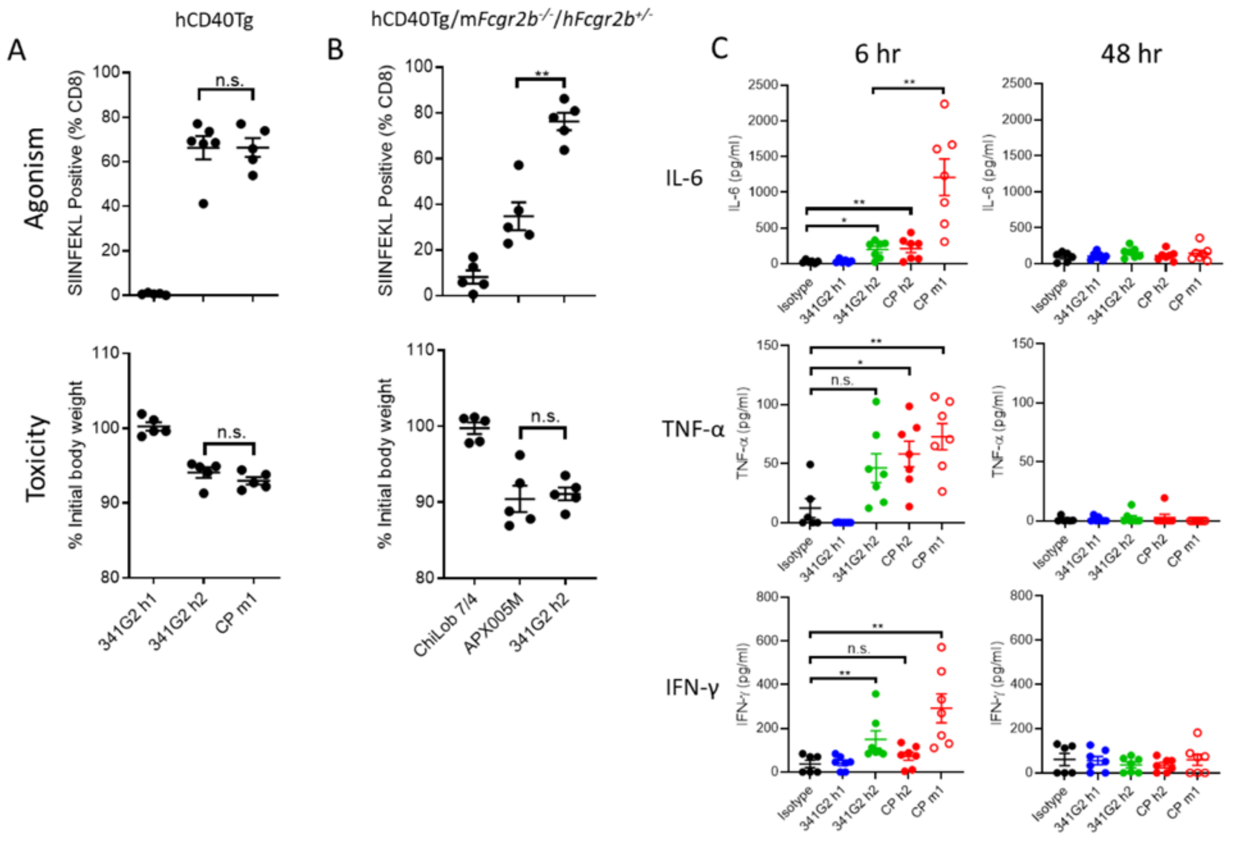

Figure S2, Related to Figure 4. 341G2 h2 induces similar levels of toxicity to other clinically-relevant anti-CD40 agonists

1x10^5 OTI cells were adoptively transferred into (A) hCD40Tg or (B) hCD40Tg/*mFcgr2b*$^{-/-}$/*hFcgr2b*$^{+/-}$ mice 1 day before treatment with 30 µg anti-CD40 mAb as indicated. Mice were bled on day 5 and SIINFEKL$^+$ cells were expressed as a percentage of total CD8$^+$ T cells. Toxicity was assessed by loss of body weight after treatment; mice were weighed before treatment and two days after anti-CD40 mAb treatment when weight loss reaches its maximum before recovery. Means ± SEM, n = 5-6. Each dot represents one mouse. Two-tailed, non-paired Student t test, *p < 0.05, **p < 0.01, ***p < 0.001. n.s. not significant.

(C) hCD40Tg mice were administered 30 µg anti-CD40 mAb intravenously and bled 6 hours and 48 hours post-injection. Serum levels of IL-6, TNF-α and IFN-γ were quantified by ELISA. Means ± SEM, n = 6-7. Each dot represents one mouse. Two-tailed, non-paired Student t test, *p < 0.05, **p < 0.01, ***p < 0.001. n.s. not significant.

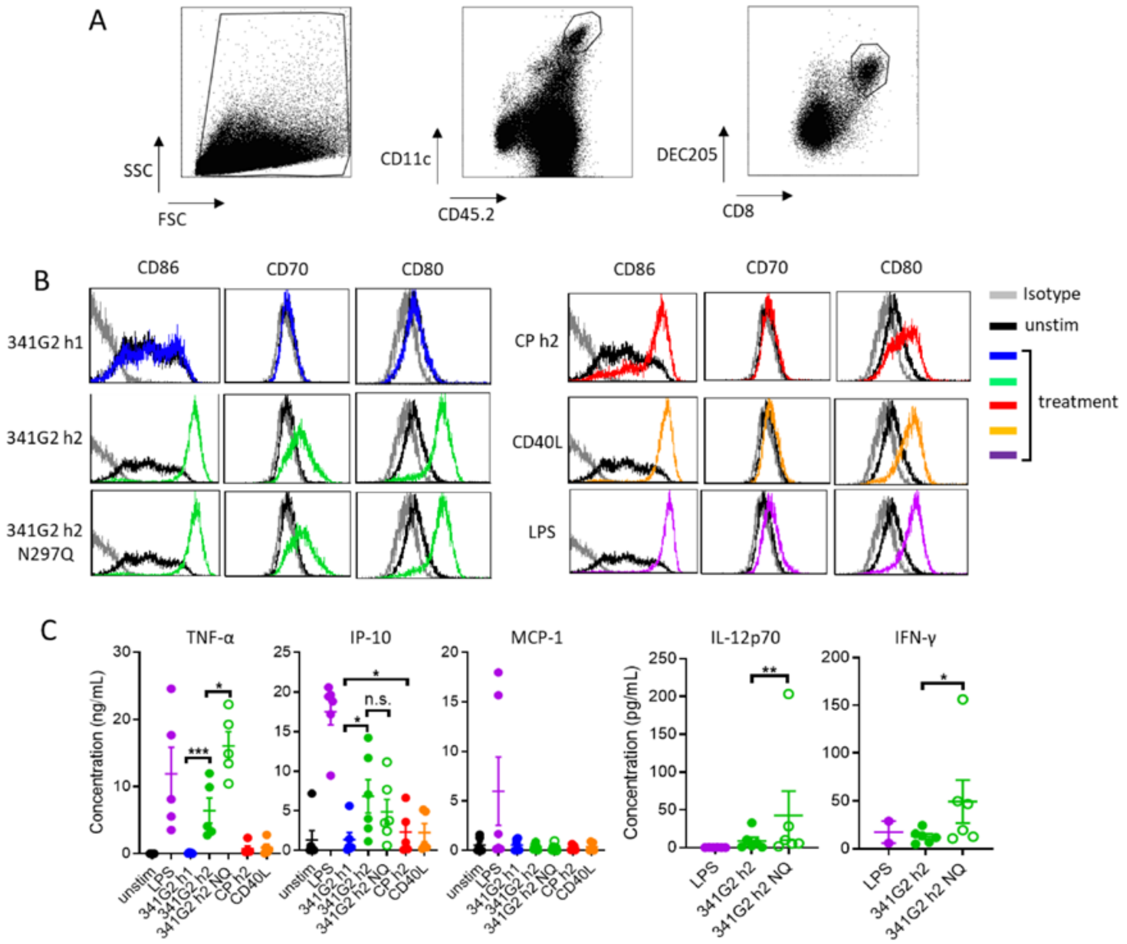

Figure S3, Related to Figure 5. 341G2 h2 activates dendritic cells

(A) Gating strategy for hCD40Tg and hCD40Tg/FcγRnull splenic CD11c^{+}CD8^{+}DEC205^{+} DC subset.

(B) Human monocyte-derived DCs were stimulated with various anti-CD40 mAb, CD40L or LPS as indicated for 48 hours and then stained for CD86, CD70 and CD80. Data representative of six donors.

(C) Experiment the same as (B). Cell culture supernatant was harvested after 48 hours and analysed for the level of TNF-α, IP-10, MCP-1, IL-12p70 and IFN-γ using Luminex. Means ± SEM, data pooled from five-six donors. Each dot represents one donor. Two-tailed, paired Student t test, *$p < 0.05$, **$p < 0.01$, ***$p < 0.001$. n.s. not significant.

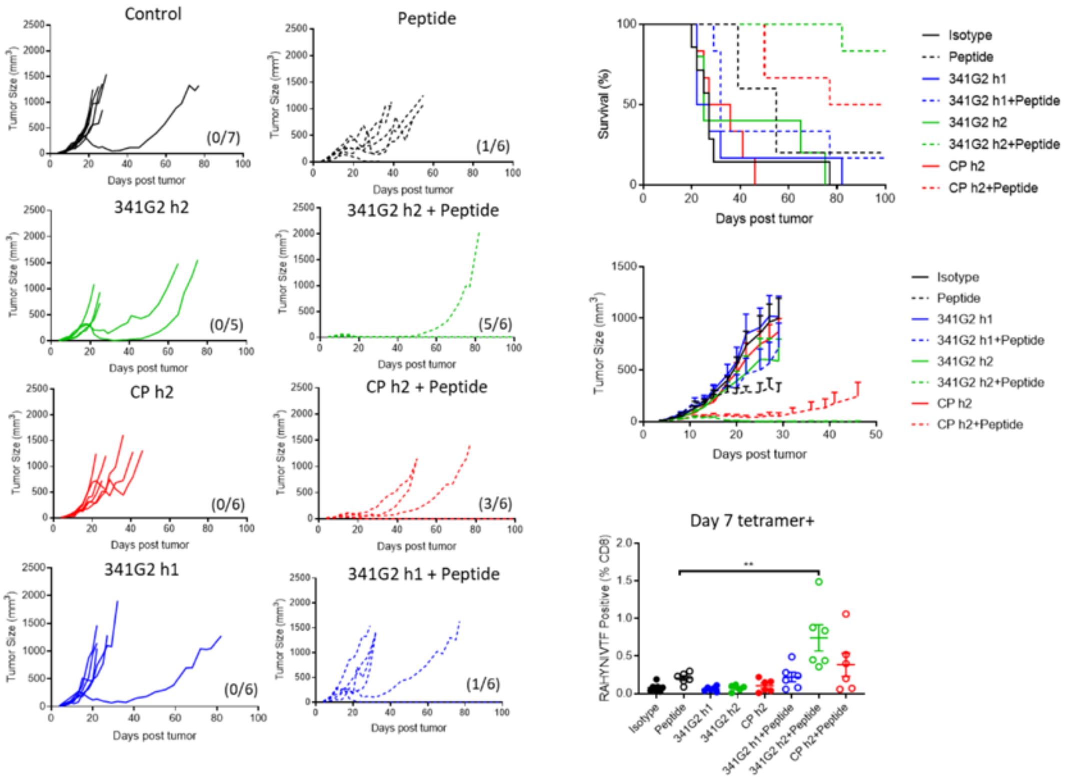

Figure S4, Related to Figure 7. Antitumor efficacy of anti-CD40 mAb in combination with peptide vaccination

hCD40Tg mice were inoculated with $1x10^5$ TC1 tumor cells subcutaneously on day 0 and then were treated with 3 µg peptide in combination with 30 µg anti-CD40 mAb on day 5, or treated with 30 µg anti-CD40 mAb alone on day 5, 8 and 11. n = 5-7, each dot represents one mouse. Two-tailed, non-paired Student t test. The fractions in parenthesis indicate the number of tumor-free mice (numerator) out of total mice (denominator) in that group at the end of the study. Survival curves were compared by Log-rank test. *$p < 0.05$, **$p < 0.01$, ***$p < 0.001$.

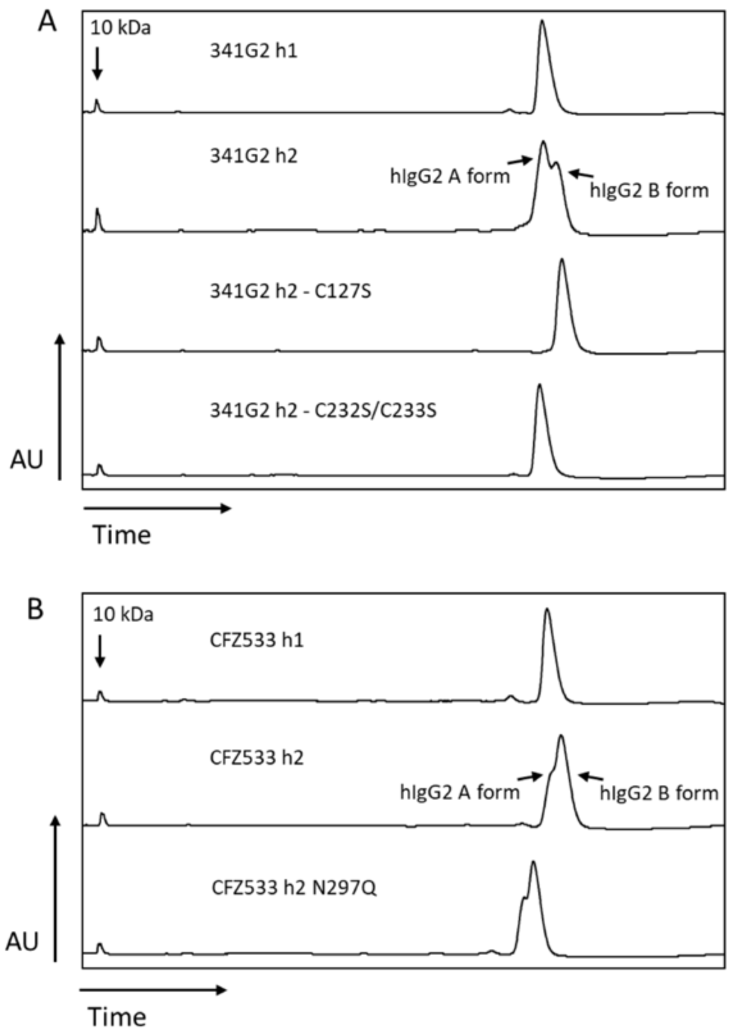

Figure S5, Related to Figure 8. CE-SDS profiles of anti-CD40 mAb

Non-reducing CE-SDS was carried out for (A) 341G2 Fc variants and (B) CFZ533 Fc variants. Data representative of at least three experiments.

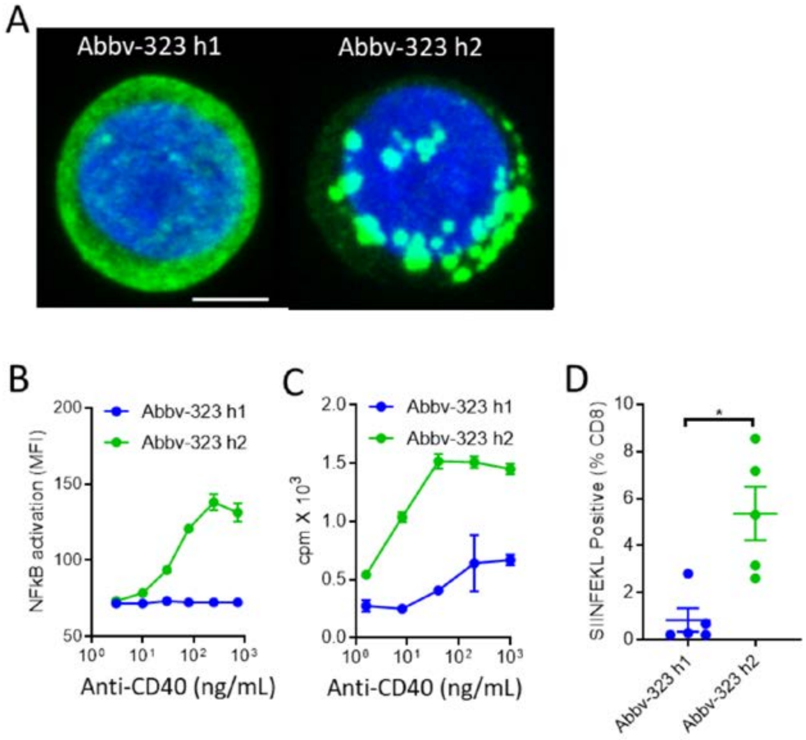

Figure S6, Related to Figure 8. Antagonist Abbv-323 is converted into an agonist by isotype switching to hIgG2

(A) Jurkat cells stably transfected with human CD40EC-GFP were treated with 10 µg/mL Abbv-323 h1 or Abbv-323 h2 for one hour at 37°C. Cells were then fixed, nuclear-stained using DAPI and imaged using a Leica SP8 confocal microscope. Z-stack images shown. Blue: nucleus; Green: human CD40-GFP. Scale bar: 4 µm. Image representative of at least ten images taken.

(B) Jurkat NFκB GFP reporter cells stably transfected with hCD40 were incubated with various concentrations of anti-CD40 mAb for 8 hours and the level of NFκB activation assessed by GFP expression using flow cytometry. n = 3, data representative of 2 experiments.

(C) Purified splenic B cells from hCD40Tg mice were incubated with various concentrations of anti-CD40 mAb for 4 days. Proliferation was measured by ^{3}H thymidine incorporation. Means ± SEM, n = 3, data representative of 3 experiments.

(D) 1x10^{5} OTI cells were adoptively transferred into hCD40Tg mice 1 day before treatment with 100 µg anti-CD40 mAb as indicated. Mice were bled on day 5 and SIINFEKL^{+} cells were expressed as the percentage of total CD8^{+} T cells. n = 5, each dot represents one mouse, data representative of two experiments. Two-tailed, non-paired Student t test, *p < 0.05, **p < 0.01, ***p < 0.001.

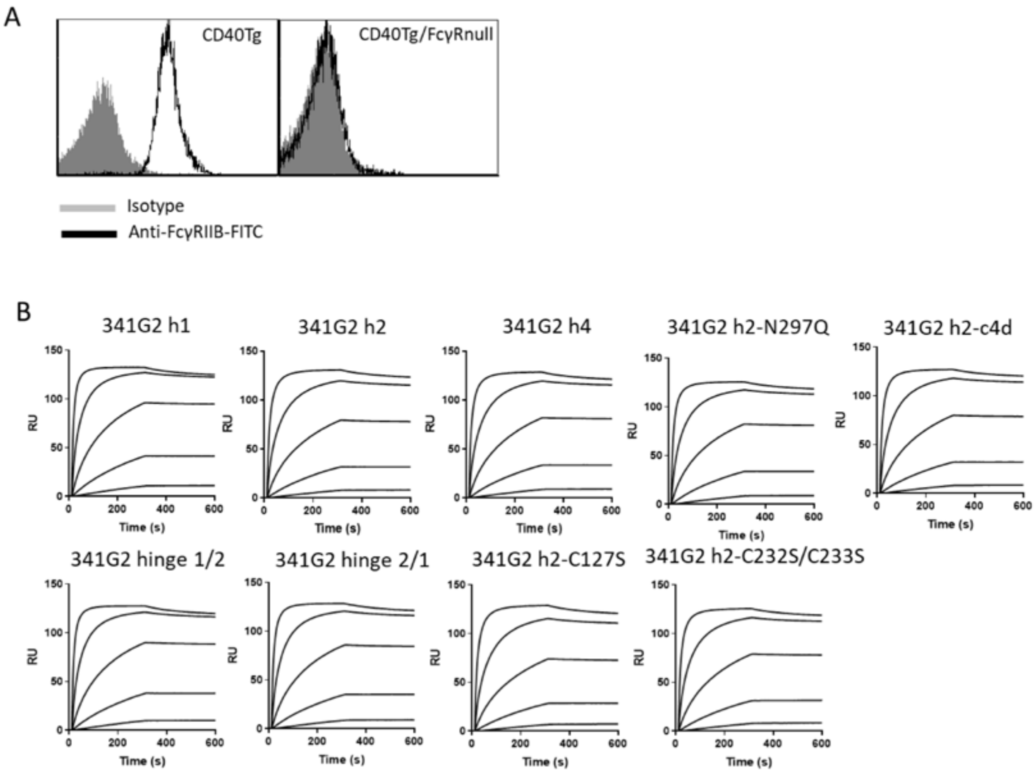

Figure S7, Related to STAR Methods. Binding analysis of 341G2 variants to hCD40 by SPR

(A) Expression of FcγRIIB on B cells from peripheral blood of hCD40Tg or hCD40Tg/FcγRnull mice analysed by anti-CD19-APC and anti-FcγRIIB-FITC, demonstrating the absence of FcγRIIB on the hCD40Tg/FcγRnull B cells.

(B) hCD40 was immobilized onto CM5 chip and various 341G2 variants were injected at 250, 50, 10, 2, 0.4, and 0 nM using Biacore T100 instrument. The association and dissociation phases lasted 300 seconds, respectively.

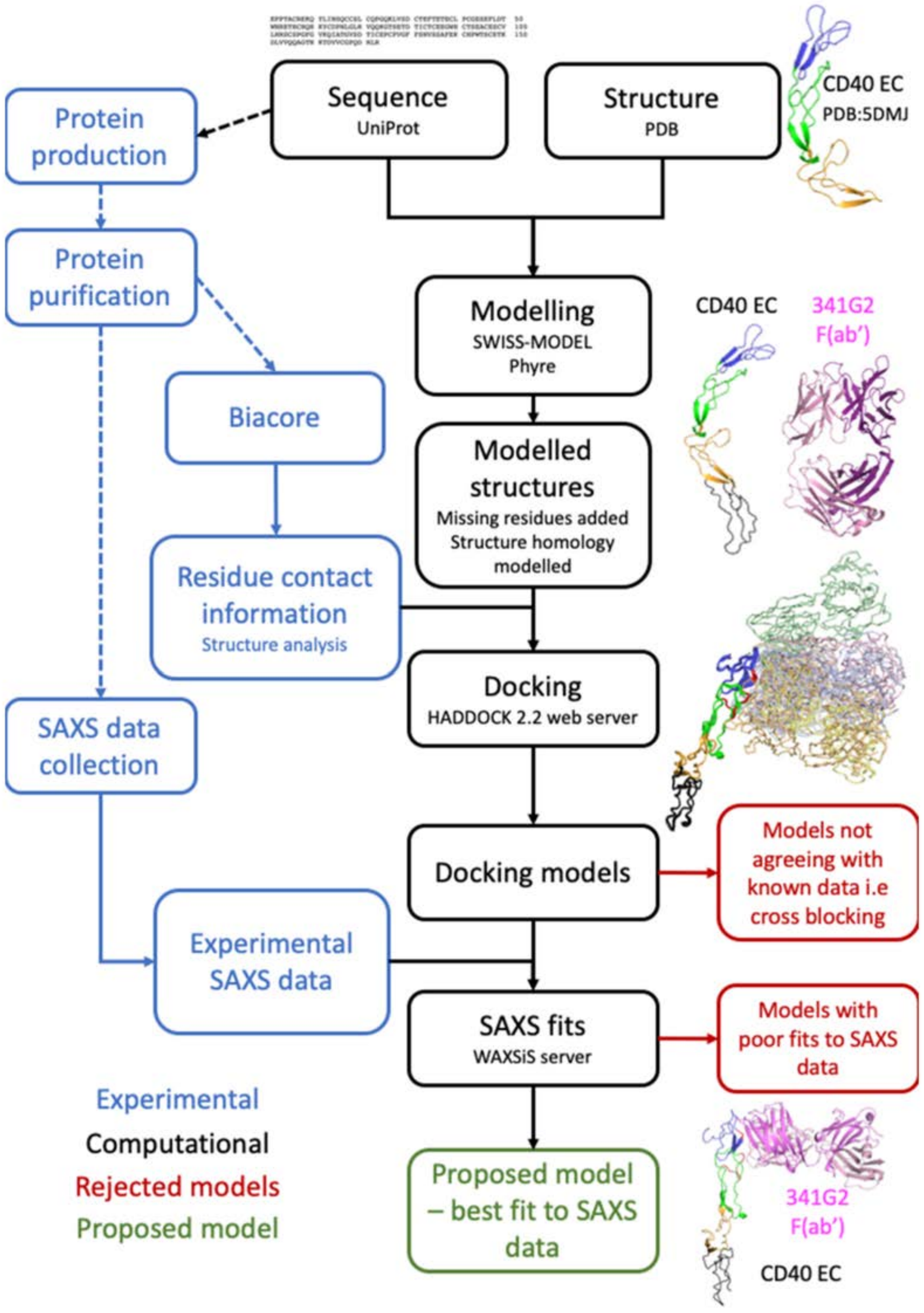
Sequence
UniProt
Structure
PDB
CD40 EC
PDB:5DMJ
Protein production
Protein purification
Modelling
SWISS-MODEL
Phyre
CD40 EC
341G2 F(ab')
Biacore
Modelled structures
Missing residues added
Structure homology modelled
Residue contact information
Structure analysis
Docking
HADDOCK 2.2 web server
SAXS data collection
Docking models
Models not agreeing with known data i.e cross blocking
Experimental SAXS data
SAXS fits
WAXSiS server
Models with poor fits to SAXS data
Experimental
Computational
Rejected models
Proposed model
Proposed model – best fit to SAXS data
341G2 F(ab')
CD40 EC

Figure S8, Related to STAR Methods. Generation of proposed binding model of 341G2 F(ab) and CD40 EC domain.

The workflow details the steps taken to generate the proposed binding model between CD40 EC and 341G2 F(ab). Initially, sequence information was examined and that used for the modelling compared with that used to generate the protein for experimental data collection, to ensure reliable SAXS fitting. Available structures within the PDB database were then examined to identify structures containing residues to cover the sequences of interest. Where no structure was available (341G2), the sequence was submitted to the SWISS-MODEL server to generate a homology model. The model was generated based on the 56.a.09 antibody (PDB: 5K9J) with which 341G2 Fab shares 90.4% sequence identity and 0.57 sequence similarity score. For CD40 - Phyre2 was used to generate a model of the CD40EC. The CD40EC sequence (Uniprot: P25942) was submitted for modelling, returning a model based on PDB: 5DMJ that had a confidence score of 100% with 97% coverage indicating a suitable model for further analysis. Docking of 341G2 F(ab) and CD40 was performed using the HADDOCK 2.2 webserver. Residues on CD40 known to affect binding (identified through experimental data collection using alanine scanning mutagenesis experiments and Biacore), and the CDR loop residues of 341G2 were provided to the docking server as restraints in the docking following the HV-Epi9 protocol. For CD40 these residues were S49, D50, F67, L68, T75, H76, H78 and Q79. For the 341G2 model, the CDR loop residues were 26 to 34, 53 to 59, 104 to 112, 260 to 268, 284 to 288 and 325 to 331. The CDR loops were defined as active residues, while the CD40 residues were defined as active with a 9Å radius of passive residues. Docking produced 10 clusters comprising 40 representative structures. Based on the knowledge that 341G2 and ChiLob 7/4 do not cross-block, a large number of the representative models were able to be excluded due to clashing with the Chilob 7/4 Fab from the crystal structure PDB: 6FAX when aligned on CD40. This left a total of 11 models from 3 clusters that were taken forward for further analysis. The remaining structures were validated against the SAXS data using the WAXSiS server to compare the structures to the experimental SAXS data. The models that best fitted the SAXS data were selected by their $\chi 2$ score. A clear trend was observed between better and worse fitting models when looking at the SAXS fits ($\chi 2$ range 2.21±0.37 to 4.50±0.47), which was characterised by a movement of the F(ab) around the CD40 surface when comparing multiple models. It is also known that 341G2 cannot bind when CD40L is present, allowing comparison with PDB: 3QD6, to ensure that either the CD40 binding interface for the binding models and CD40L are shared or steric clashes occur between CD40L and the F(ab). In this case all models showed clashes with CD40L. The model showing the best fit to the SAXS data ($\chi 2$ = 2.21±0.37) was therefore selected as the proposed binding model, as it showed no cross blocking with ChiLob7/4, clashed with CD40L and made contact with residues shown to be critical for binding from the biacore mutagenesis data.

Table S1, Related to STAR Methods. Affinity of 341G2 variants for hCD40

	k_{on} (s^{-1} M^{-1})	k_{off} (s^{-1})	KD (M)	Standard Deviation
**341G2 hg1**	6.17×10^{-5}	2.38×10^{-4}	3.87×10^{-10}	1.78×10^{-10}
**341G2 hg2**	4.39×10^{-5}	2.47×10^{-4}	5.58×10^{-10}	2.24×10^{-10}
**341G2 hg4**	4.77×10^{-5}	2.21×10^{-4}	4.60×10^{-10}	1.90×10^{-10}
**341G2 hg2-NQ**	4.95×10^{-5}	2.37×10^{-4}	4.77×10^{-10}	2.17×10^{-10}
**341G2 hg2-c4d**	4.66×10^{-5}	2.33×10^{-4}	5.04×10^{-10}	2.43×10^{-10}
**341G2 hinge 1/2**	6.34×10^{-5}	2.47×10^{-4}	3.80×10^{-10}	1.25×10^{-10}
**341G2 hinge 2/1**	5.37×10^{-5}	2.45×10^{-4}	4.47×10^{-10}	1.79×10^{-10}
**341G2 h2-C127S**	4.18×10^{-5}	2.57×10^{-4}	6.17×10^{-10}	2.14×10^{-10}
**341G2 h2-C232S/C233S**	4.58×10^{-5}	2.21×10^{-4}	4.77×10^{-10}	1.87×10^{-10}
